# The Inhibitory Response to PI3K/AKT Pathway Inhibitors MK-2206 and Buparlisib Is Related to Genetic Differences in Pancreatic Ductal Adenocarcinoma Cell Lines

**DOI:** 10.3390/ijms23084295

**Published:** 2022-04-13

**Authors:** Yixuan Ma, Sina Sender, Anett Sekora, Weibo Kong, Peter Bauer, Najim Ameziane, Ruslan Al-Ali, Susann Krake, Mandy Radefeldt, Frank Ulrich Weiss, Markus M. Lerch, Alisha Parveen, Dietmar Zechner, Christian Junghanss, Hugo Murua Escobar

**Affiliations:** 1 Department of Medicine Clinic III, Hematology, Oncology and Palliative Medicine, Rostock University Medical Center, Ernst-Heydemann-Str. 6, 18057 Rostock, Germany; yixuan.ma@med.uni-rostock.de (Y.M.); sina.sender@med.uni-rostock.de (S.S.); anett.sekora@med.uni-rostock.de (A.S.); kong@fbn-dummerstorf.de (W.K.); peter.bauer@centogene.com (P.B.); christian.junghanss@med.uni-rostock.de (C.J.); 2 Institute of Muscle Biology and Growth, Research Institute for Farm Animal Biology (FBN), 18196 Dummerstorf, Germany; 3 CENTOGENE GmbH, 18057 Rostock, Germany; najim.ameziane@arcensus-diagnostics.com (N.A.); ruslan.al-ali@centogene.com (R.A.-A.); susann.krake@centogene.com (S.K.); mandy.radefeldt@centogene.com (M.R.); 4 Arcensus GmbH, 18055 Rostock, Germany; 5 Department of Medicine A, University Medicine, University of Greifswald, 17475 Greifswald, Germany; ulrich.weiss@med.uni-greifswald.de (F.U.W.); markus.lerch@med.uni-muenchen.de (M.M.L.); 6 LMU Munich University Hospital, 81377 Munich, Germany; 7 Institute for Experimental Surgery, University of Rostock, 18057 Rostock, Germany; alisha.parveen@med.uni-rostock.de (A.P.); dietmar.zechner@uni-rostock.de (D.Z.)

**Keywords:** PI3K/AKT pathway, pancreatic ductal adenocarcinoma, *KRAS*, *TP53*

## Abstract

The aberrant activation of the phosphoinositide 3-kinase (PI3K)/ protein kinase B (AKT) pathway is common in pancreatic ductal adenocarcinomas (PDAC). The application of inhibitors against PI3K and AKT has been considered as a therapeutic option. We investigated PDAC cell lines exposed to increasing concentrations of MK-2206 (an AKT1/2/3 inhibitor) and Buparlisib (a pan-PI3K inhibitor). Cell proliferation, metabolic activity, biomass, and apoptosis/necrosis were evaluated. Further, whole-exome sequencing (WES) and RNA sequencing (RNA-seq) were performed to analyze the recurrent aberrations and expression profiles of the inhibitor target genes and the genes frequently mutated in PDAC (Kirsten rat sarcoma virus (*KRAS*), Tumor protein p53 (*TP53*)). MK-2206 and Buparlisib demonstrated pronounced cytotoxic effects and limited cell-line-specific effects in cell death induction. WES revealed two sequence variants within the direct target genes (*PIK3CA* c.1143C > G in Colo357 and *PIK3CD* c.2480C > G in Capan-1), but a direct link to the Buparlisib response was not observed. RNA-seq demonstrated that the expression level of the inhibitor target genes did not affect the efficacy of the corresponding inhibitors. Moreover, increased resistance to MK-2206 was observed in the analyzed cell lines carrying a *KRAS* variant. Further, increased resistance to both inhibitors was observed in SU.86.86 carrying two *TP53* missense variants. Additionally, the presence of the *PIK3CA* c.1143C > G in *KRAS*-variant-carrying cell lines was observed to correlate with increased sensitivity to Buparlisib. In conclusion, the present study reveals the distinct antitumor effects of PI3K/AKT pathway inhibitors against PDAC cell lines. Aberrations in specific target genes, as well as *KRAS* and *TP53*, individually or together, affect the efficacy of the two PI3K/AKT pathway inhibitors.

## 1. Introduction

Pancreatic ductal adenocarcinoma (PDAC) is one of the most aggressive human cancer types and is currently the fourth leading cause of cancer-related deaths in both men and women [1]. Due to the difficulty of early diagnosis, the lack of effective treatments, the prevalence of tumor metastasis and relapse, and chemoresistance, the cure rate for pancreatic cancer is only 9% [2]. Furthermore, PDAC is expected to become the third most fatal cancer within decades [3]. Without treatment, the median survival time of patients with metastatic pancreatic cancer is only 3 months [2,4,5,6,7]. Although extensive research has been carried out in recent years, there have been only slight improvements in disease prognosis; the median survival is still less than 12 months, and the overall 5-year survival rate recently increased to only 10% [1].

The phosphoinositide 3-kinase (PI3K)/protein kinase B (AKT) pathway is an intracellular signaling pathway important in regulating the cell cycle. PI3Ks have been reported to be involved in several cell functions, such as cell growth, proliferation, differentiation and intracellular trafficking, which in turn contribute to cancer development [8]. Additionally, studies indicate that PI3Ks play important roles in cancer metastasis in several types of cancers, including colon cancers, breast cancers, and pancreatic cancers [9,10,11]. PI3Ks can be activated by growth factor stimulation, which results in the activation of AKTs. The activated AKTs affect cellular proliferation or survival through several downstream signaling pathways, such as activating the pathway for the nuclear factor kappa-light-chain-enhancer of activated B cells (NF-κB), or suppressing the p53 pathway [12]. Therefore, the PI3K/AKT pathway is directly related to cellular quiescence, proliferation, malignancy, and longevity. The activation of the PI3K/AKT pathway is implicated in human cancer and is perhaps the most commonly activated signaling pathway [13]. It is estimated that 60% of all PDAC patients have deregulation of the PI3K/AKT signaling pathway [14]. Increased activation of the PI3K/AKT pathway has been noted in more than 40% of PDAC cases and has been associated with a poorer prognosis [15,16]. Furthermore, several studies indicate that the PI3K/AKT pathway contributed to the chemoresistance of cancer cells by activating NF-κB [17,18].

Since the PI3K/AKT pathway plays a critical role in the development and prognosis of PDAC, inhibiting the activation of the PI3K/AKT pathway has become a focus for PDAC therapy. Furthermore, the inhibition of the PI3K/AKT pathway also enhances the chemosensitivity of PDAC cell lines in vitro and in vivo [19]. Key proteins such as PI3Ks and AKTs are considered therapeutic targets. A number of studies have shown that, whether used alone or in combination, PI3K and AKT inhibitors are reported to achieve promising effects in PDAC treatment [14]. Ihle et al. reported that the pan-PI3K inhibitor PX-866 displayed good antitumor activity against Kirsten rat sarcoma virus (*KRAS*) wild-type PDAC cell line BxPC-3 in vivo model, while PX-866 showed a slight effect against *KRAS* mutant PDAC cell lines Panc-1 and MIA Paca-2 [20]. Another study reported that the use of the AKT1/2/3 inhibitor GSK690693 to inhibit AKTs has also observed satisfactory anti-proliferative effects in PDAC cell lines [21]. Therefore, several PI3K inhibitors (e.g., the pan-PI3K inhibitors XL147, PX-866, Buparlisib, and GDC-0941, as well as the PI3Kδ-specific inhibitor CAL101) and AKT inhibitors (e.g., the ATP-competitive AKT inhibitor AZD5363 and the Allosteric AKT inhibitor MK-2206) have entered clinical trials, and some of them have achieved an acceptable response [22,23,24].

Due to the promising results shown by PI3K/AKT inhibition in PDAC experiments and clinical trials, we investigated the cytostatic/cytotoxic- and apoptosis/necrosis-inducing effects of the AKT1/2/3 inhibitor (MK-2206) and the pan-PI3K inhibitor (Buparlisib) in ten PDAC cell lines (AsPc-1, BxPc-3, Capan-1, Panc-1, PaTu8902, PaTu8988T, PaTu8988S, SU.86.86, T3M4, and Colo357). In addition, all cell lines were characterized by whole-exome sequencing (WES) and RNA-seq transcriptome analysis. *KRAS* and *TP53* are the two most important and most frequently mutated genes among all PDAC hotspot genes, and the mutation rates in PDAC are approximately 92% and 70%, respectively [25,26]. Both of them are not only involved in the tumorigenesis and development of PDAC but also play an important role in tumor resistance and relapse [25,27]. Moreover, *KRAS* and *TP53* also interact to increase the malignancy of tumors, including immune evasion, which results in poor patient prognosis [28]. Here, we explore how these genes affect the response of PDAC cell lines to PI3K/AKT inhibitors. Further, we integrated these genetic data and the inhibitor response to explore their relationship.

## 2. Results

### 2.1. Analysis of the Cytotoxic Effects of MK-2206 and Buparlisib in PDAC Cell Lines

**Figure 1:**
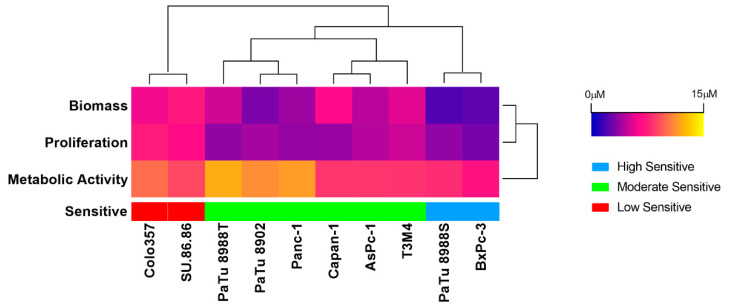
IC50 values when assessing proliferation, metabolic activity, and cell biomass after 72 h MK-2206 exposure in ten PDAC cell lines, as well as the classification of these cell lines by k-means++ (unsupervised machine learning algorithm) into low (red), moderate (green), and high (blue) groups.

When treating the PDAC cell lines with the AKT1/2/3 inhibitor MK-2206 for 72 h, the cell proliferation and biomass of PDAC were significantly inhibited, starting at a concentration of 1 μM (Supplementary Figure S1 and Supplementary Table S1). However, the inhibition of cell metabolic activities was less pronounced than the inhibition of cell proliferation and biomass. The half-maximum inhibitory concentration (IC50) values ranged from 2.943 μM to 7.508 μM (proliferation), 7.233 μM to 12.15 μM (metabolic activity), and 2.024 μM to 7.340 μM (biomass) (Figure 1 and Figure S2 and Supplementary Table S2).

These IC50 values were clustered by unsupervised machine learning into three sensitivity groups: low (Colo357 and SU.86.86), moderate (PaTu8988T, PaTu8902, Panc-1, Capan-1, AsPc-1, and T3M4), and high (PaTu8988S and BxPc-3) sensitivity groups (Figure 1 and Figure S2).

**Figure 2:**
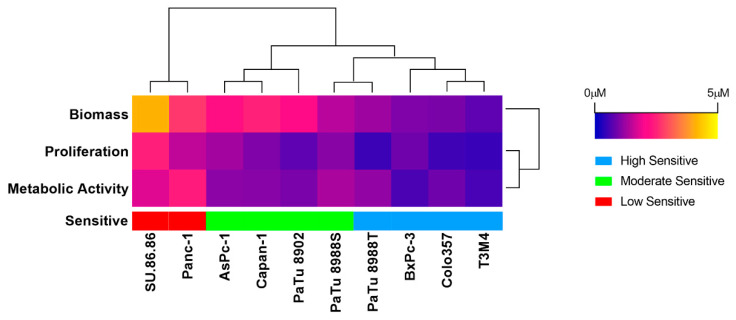
IC50 values when assessing proliferation, metabolic activity, and cell biomass after 72 h Buparlisib exposure in ten PDAC cell lines, as well as the classification of these cell lines by k-means++ (unsupervised machine learning algorithm) into low (red), moderate (green), and high sensitivity (blue) groups.

When treating the cell lines with the Pan-PI3K inhibitor Buparlisib for 72 h, it significantly inhibited cell proliferation, metabolic activity, and cell biomass at a concentration of 0.5 μM (Supplementary Figure S3 and Supplementary Table S3). In the three viability assays, Buparlisib demonstrated a similarly efficient inhibition of cell proliferation and metabolic activity. The IC50 values ranged from 0.4741 μM to 2.469 μM (proliferation), 0.7471 μM to 4.098 μM (metabolic activity), and 0.5916 μM to 2.419 μM (biomass) (Figure 2 and Figure S4 and Supplementary Table S4).

Based on the same method described above, ten PDAC cell lines were separated into three groups: low (Panc-1 and SU.86.86), moderate (AsPc-1, Capan-1, PaTu8902, and PaTu8988S), and high (BxPc-3, Colo357, PaTu8988T, and T3M4) sensitivity groups (Figure 2 and Figure S4).

### 2.2. Analysis of MK-2206 and Buparlisib in Inducing Apoptosis/Necrosis of PDAC Cell Lines

MK-2206 induced a significant increase in cell death only in AsPc-1 (10 μM), BxPc-3 (1 μM), and Colo357 (10 μM). In addition, in all cell lines, even in AsPc-1, BxPc-3, and Colo357, the observed percentage of dead cells was less than 20% at all tested concentrations (Supplementary Figure S5 and Supplementary Table S5). Compared to the DMSO control group, the percentages of dead cells were decreased in all exposure groups of PaTu8988S.

Buparlisib induced apoptosis/necrosis in all tested PDAC cell lines. Compared with the DMSO control group, a significant induction effect was observed, starting at 1 μM. When Buparlisib concentrations reached 5 μM, more than 50% of AsPc-1, BxPc-3, and T3M4 cells were dead. However, although we observed a significant induction of cell death in Panc-1, SU.86.86, and PaTu8988T, the percentage of apoptotic/necrotic cells was still less than 20% even at the highest tested concentration (10 μM) (Supplementary Figure S6 and Supplementary Table S6).

### 2.3. Gene Expression and Genetic Variants of MK-2206 or Buparlisib Target Genes

**Figure 3:**
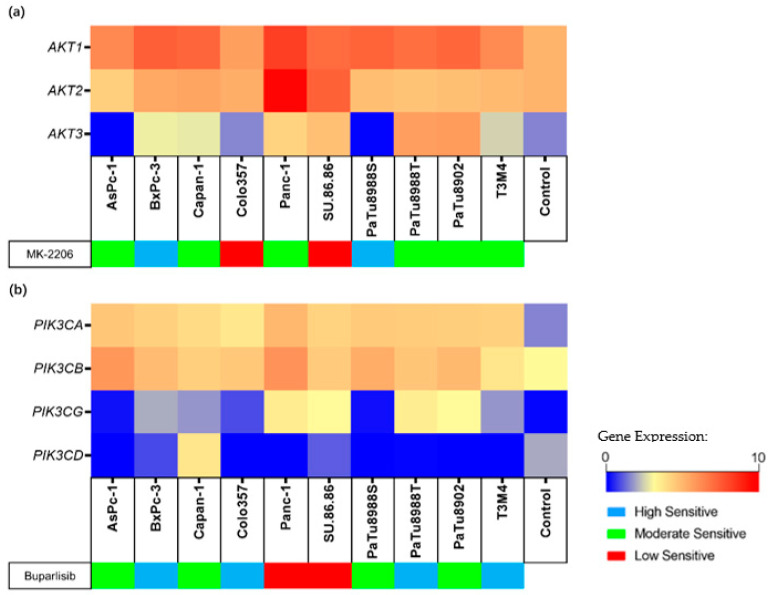
Gene expression levels of inhibitor target genes in cell lines and control. The different sensitivities to MK-2206 (**a**) and Buparlisib (**b**) are indicated for each cell line. Gene expression levels are displayed as Log_2_ (TPM + 1). Control: non-neoplastic pancreatic tissue. Gene expression in normal pancreatic tissue comes from GTEx and TCGA databases.

The transcriptional activity of the target genes for each inhibitor (for MK-2206: *AKT1, AKT2, AKT3*; for Buparlisib: *PIK3CA, PIK3CB, PIK3CG, PIK3CD*) was evaluated in all cell lines by RNA-seq. The expression level was displayed as Log_2_ (TPM + 1) (Figure 3). Specifically, *AKT2*, *AKT3*, *PIK3CG*, and *PIK3CD* demonstrated a lower expression than non-neoplastic control (Supplementary Table S7). These low-expressed genes and cell lines were as follows (expression minimum-maximum vs. control): *AKT2* in AsPc-1, PaTu8988S, PaTu8988T, PaTu8902, and T3M4 (4.32–4.91 vs. 5.13); *AKT3* in AsPC-1 and PaTu8988S (0.00–0.07 vs. 1.52); *PIK3CG* in AsPc-1, Colo357, Panc-1, PaTu8988T, PaTu8988S, PaTu8902, and T3M4 (0.00–0.10 vs. 0.12); and *PIK3CD* in AsPc-1, Colo357, and PaTu8988S (0.24–0.89 vs. 1.08). In addition, the expression of these target genes was higher in other PDAC cell lines than in the control. In particular, the expression of *AKT1* (6.66–8.96 vs. 5.13), *PIK3CA* (3.68–4.97 vs. 1.52), and *PIK3CB* (3.73–6.00 vs. 3.10) was higher than the control in all cell lines.

The identical target genes for MK-2206 (*AKT1, AKT2, AKT3*) and Buparlisib (*PIK3CA, PIK3CB, PIK3CG, PIK3CD*) were selected to analyze transcript variants by WES.

When focusing on MK-2206 target genes, initially a total of nine variants, including four *AKT1* variants, two *AKT2* variants, and three *AKT3* variants, were identified in ten PDAC cell lines (Supplementary Table S8). Of these nine variants, one was identified in BxPc-3, Panc-1, PaTu8988T, and PaTu8902; two were identified in SU.86.86; and three were identified in PaTu8988S. Variant filtering according to Method 4.8 classified none of the identified variants as potentially affecting the protein-coding sequence leading to aberrant protein function.

**Figure 4:**
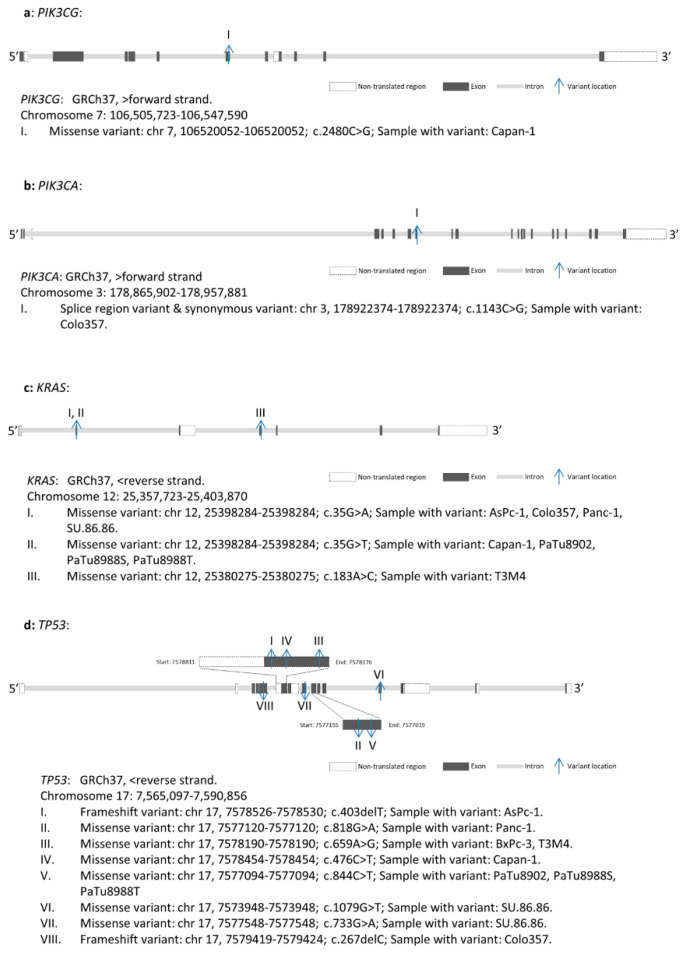
Gene maps indicating the variant positions of *PIK3CG* (**a**), *PIK3CA* (**b**), *KRAS* (**c**), and *TP53* (**d**) in different PDAC cell lines. GRCh37: Genome Reference Consortium Human Build 37, Chr: chromosome.

When focusing on Buparlisib target genes, a total of 17 variants, including six *PIK3CA* variants, eight *PIK3CB* variants, one *PIK3CG* variant, and two *PIK3CD* variants, were identified (Supplementary Table S9). Of these seventeen variants, one was identified in Panc-1, PaTu8988T, PaTu8902, SU.86.86, and T3M4; two were identified in AsPc-1 and Capan-1; and eight were identified in Colo357. Variant filtering according to Method 4.8 classified that the missense variant *PIK3CG* c.2480C > G in Capan-1 and the splice region variant and synonymous variant *PIK3CA* c.1143C > G in Colo357 influenced the primary structure of the respective proteins; therefore, they were classified for further analysis (Figure 4a,b).

### 2.4. KRAS and TP53 Gene Variants Were Observed in PDAC Cell Lines

#### 2.4.1. KRAS Variants and Expression in PDAC Cell Lines

WES demonstrated *KRAS* variants in nine of the ten tested PDAC cell lines (Figure 4c, Supplementary Table S10). Three different *KRAS* variants were identified, and all of them were missense variants. *KRAS* c.35G > A (p.Gly12Asp) was identified in AsPc-1 (Variant allele frequency (VAF): 100), Colo357 (VAF: 23.8), Panc-1 (VAF: 62.1), and SU.86.86 (VAF: 83.7). *KRAS* c.35G > T (p.Gly12Val) was identified in Capan-1 (VAF: 97.1), PaTu8902 (VAF: 100), PaTu8988S (VAF: 96.9), and PaTu8988T (VAF: 98). *KRAS* c.183A > C (p.Gln61His) was identified in T3M4 (VAF: 32.6).

**Figure 5:**
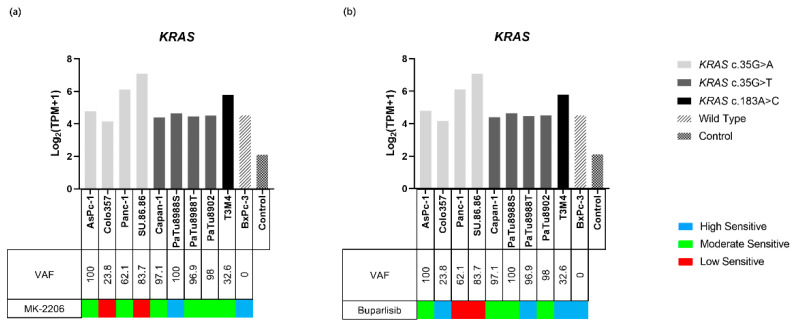
Gene expression of *KRAS* in ten PDAC cell lines and the control. The sensitivity to MK-2206 (**a**), Buparlisib (**b**), and the variants of *KRAS* are indicated for each cell line. Gene expressions are displayed as Log_2_ (TPM + 1). Compared with the control group, expression levels of *KRAS* were increased in all cell lines.

The expression of *KRAS* in all PDAC cell lines was higher than in the control (4.16–7.09 vs. 2.14) (Figure 5a,b). Both the lowest and highest *KRAS* expressions were observed in the *KRAS* c.35G > A variant; they were identified in Colo357 (4.61) and SU.86.86 (7.09), respectively. The expression of all *KRAS* c.35G > T mutations, which were identified in Capan-1, PaTu8988S, PaTu8988T, and PaTu8902, was similar to wild type BxPc-3 (4.40, 4.65, 4.46, 4.51 vs. 4.53, respectively), and the expressions of *KRAS* c183A > C in T3M4 and *KRAS* c.35G > A in AsPc-1, Panc-1, and SU.86.86 were higher than wild type BxPc-3 (4.79–7.09 vs. 4.53).

#### 2.4.2. KRAS and Inhibitor Response

A comprehensive comparison of the sensitivity and the *KRAS* status of all cell lines revealed that *KRAS* variants alone have no major influence on the inhibitory effect of Buparlisib, since cell lines harboring a *KRAS* mutation were classified into all sensitivity groups (Figure 5b). Moreover, the four cell lines in the highly sensitive group contained all three *KRAS* mutant and wild-type cell lines. For MK-2206, the results were different. The highly sensitive group contained only wild-type and one *KRAS* mutant cell line, while the rest of the *KRAS*-mutant-carrying cell lines were distributed in the moderate or low sensitivity groups, indicating that PDAC cell lines carrying the *KRAS* variant were less sensitive to MK-2206 (Figure 5a). *KRAS* gene expression and VAF did not affect the efficacy of the two inhibitors.

#### 2.4.3. TP53 Variants and Expression in PDAC Cell Lines

Two different types of variants, including frameshift (fs) variants and missense variants of *TP53*, were identified in the PDAC cell lines (Figure 4d, Supplementary Table S11). The fs variants *TP53* c.403delT (p.Cys135fs) and *TP53* c.267delC (p.Ser90fs) were identified in AsPc-1 (VAF: 96.4) and Colo357 (VAF: 100), respectively. The missense variants *TP53* c.476C > T (p.Ala159Val) and *TP53* c.818G > A (p.Arg273His) were identified in Capan-1 (VAF: 100) and Panc-1 (VAF: 98.8), respectively. Double missense mutations, including *TP53* c.733G > A (p.Gly245Ser) and *TP53* c.1079G > T (p.Gly360Val), were identified in SU.86.86 (VAF: 100, 100, respectively). *TP53* c.659A > G (p.Tyr220Cys) was identified in BxPC-3 (VAF: 99) and T3M4 (VAF: 100). *TP53* c.844C > T (p.Arg282Trp) was identified in PaTu8902 (VAF: 100), PaTu8988S (VAF: 100), and PaTu8988T (VAF: 100).

**Figure 6:**
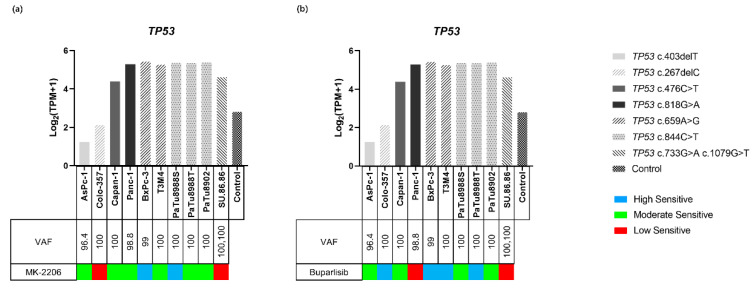
Gene expression of *TP53* in ten PDAC cell lines and the control. The sensitivity to MK-2206 (**a**), Buparlisib (**b**), and the variants of *TP53* are indicated for each cell line. Gene expressions are displayed as Log_2_ (TPM + 1). Missense variants were related to overexpression, while frameshift variants were related to the inhibition of gene expression.

The expression levels of *TP53* with fs variants were lower than that of missense variants (1.24–2.13 vs. 4.39–5.42) and normal controls (2.83) (Figure 6a,b).

#### 2.4.4. TP53 and Inhibitor Response

A comprehensive comparison of the sensitivity to both inhibitors and the *TP53* status of all cell lines revealed no obvious relationship between the status of this tumor suppressor gene and the efficacy of the inhibitors. Interestingly, SU.86.86, which carries two missense variants in *TP53*, was classified in the low-response group for both inhibitors (Figure 6a,b). Further, *TP53* gene expression and VAF did not affect the efficacy of the two inhibitors.

## 3. Discussion

Our study demonstrated that the proliferation, metabolic activity, and cell biomass of all PDAC cell lines decreased in a dose-dependent manner after Buparlisib exposure. It is reported that Buparlisib is a potent and highly specific oral pan-class I PI3K inhibitor in low concentrations: the IC50s of Buparlisib inhibit p110α/β/δ/γ with values of 52 nM/166 nM/116 nM/262 nM in cell-free assays, respectively [29]. In addition, at high concentrations (>5 μM), it might cause cell death by binding to tubulin, thus inhibiting tubulin polymerization [30]. However, in our study, significant inhibition mostly occurred at a concentration of 1 μM. In addition, the IC50 values of all cell viability assays were below 5 μM. These results suggest that Buparlisib can exert cytotoxic effects in PDAC cell lines by inhibiting PI3Ks. Furthermore, a comprehensive analysis of WES and RNA-seq transcriptome analysis revealed that the *PIK3CG* c.2480C > G variant was correlated with gene overexpression in the corresponding cell line, whereas *PIK3CA* c.1143C > G was associated with a corresponding decrease in gene expression in tumor cell lines, but at a level still higher than non-neoplastic controls (Figure 3b). However, the sensitivity grouping demonstrated that the cell lines carrying these two gene aberrations did not display a specific response to the inhibitory effect of Buparlisib. Therefore, these results suggest that the presence of mutations in these two genes alone does not affect the inhibitory effect of Buparlisib.

This study also confirmed that MK-2206 inhibited cell proliferation, metabolic activity, and biomass in a dose-dependent manner. However, the effects of apoptosis/necrosis induction were not distinct, and the percentage of dead cells was less than 20% at all tested concentrations in all cell lines. These results indicate that the efficacy of MK-2206 at inhibiting PDAC cell lines is not mainly caused by the induction of apoptosis/necrosis. Moreover, our experiments have also revealed that the anti-proliferative and cytotoxic effects of MK-2206 are similar to, but nevertheless differ from, the observed metabolic effects, especially in Panc-1, PaTu8902, and PaTu8988T. It has been reported that some inhibitors induce cellular stress that alters cellular metabolic activity, and we observed similar properties with MK-2206 [31,32]. This result suggests that conclusions based on metabolic activity assays (e.g., WST-1, CCK8, etc.) need to be validated with other assays when MK-2206 is used. In addition, we did not find any amino acid substitution of *AKT*s in PDAC. At the same time, transcriptomic analysis did not support the hypothesis that the expression level of *AKT*s affects the efficacy of MK-2206. However, *AKT2* expression seems to affect the efficacy of Buparlisib. Two cell lines with high *AKT2* expression, Panc-1 and Su.86.86, have low sensitivity to Buparlisib. As reported, not only does the overexpression of *AKT2* represent a biological indicator of PDAC aggressiveness, but also *AKT2* plays a critical role in the inhibitor resistance of PDAC [16,33,34]. Our data indicate that high expression of *AKT2* is related to reducing the efficacy of Buparlisib. However, further functional experiments are still needed to verify the relationship between high *AKT2* expression and Buparlisib resistance. Moreover, according to cBioPortal, although *AKT2* aberration occurred in only 3.99% (49/1228) of patients with PDAC, in 87.76% (43/49) of them, the overexpression of the genetic modulation of *AKT2* was observed [35]. An analysis of the functional relationship between AKT2 aberrations and Buparlisib efficacy remains to be completed.

We identified three different amino acid substitution variants of *KRAS* in nine of ten PDAC cell lines, including *KRAS* p.12Gly > Asp (c.35G > A), *KRAS* p.12Gly > Val (c.35G > T), and *KRAS* p.Gln61His (c.183A > C). In addition, it has been reported that *KRAS* mutations can be found in approximately 92% of pancreatic cancers, and patients with *KRAS* mutations showed a bad response to first-line gemcitabine-based therapy and presented a poor prognosis [36,37]. However, relevant studies on *KRAS* variants and PDAC cell lines and on patients’ responses to PI3K/AKT pathway inhibitors are currently lacking. A comprehensive analysis of the Buparlisib sensitivity groups and *KRAS* variants did not demonstrate any relationship. This is obvious, especially in the high sensitivity group, which included not only cell lines carrying *KRAS* variants but also a wild-type *KRAS*. These results suggest that the *KRAS* status alone does not influence the sensitivity to Buparlisib in PDAC cell lines. On the other hand, analysis of MK-2206 demonstrated that carrying the *KRAS* variant appeared to cause a decrease in the sensitivity of PDAC cell lines to this inhibitor. Consistent with these data, one study demonstrated that, in cell lines of colorectal cancer, lung cancer, breast cancer, and melanoma, *KRAS* mutations were associated with significant resistance to AKT1/2 inhibition [38]. This resistance is achieved through the activation of MEK/ERK by KRAS, which bypasses PI3K/AKT and directly activates 4E-BP1 [38]. The present study suggests that this mechanism also exists in PDAC cell lines. Therefore, it might be important to consider *KRAS* status before using MK-2206 to treat patients with PDAC.

We identified two *PI3K* variants (*PIK3CA* c.1143C > G and *PIK3CG* c.2480C > G) in PDAC cell lines. We further analyzed the response of cell lines carrying *PI3K* and *KRAS* double mutations and a *KRAS* single mutation to Buparlisib. In four cell lines carrying the *KRAS* c.35G > A mutation (AsPc-1, Colo357, Panc-1, and SU.86.86), we identified that Colo357 also carries the *PIK3CA* c.1143C > G variant. Interestingly, Colo357 was highly sensitive to Buparlisib, while the other three cell lines were less sensitive. This might indicate that there are unknown interactions between the *PIK3CA* c.1143C > G variant and the *KRAS* c.35G > A variant. This *PIK3CA* variant could reduce the negative effects of *KRAS* on the sensitivity to Buparlisib. However, we did not observe any interaction when analyzing another PI3K mutation (*PIK3CG* c.2480C > G) in cell lines bearing the *KRAS* c35G > T variant (Capan-1, PaTu8902, PaTu8988S, and PaTu8988T) when using either inhibitor. However, cBioPortal demonstrated that only 2.5% (31/1228) of patients with PDAC harbor *PIK3CA* and *KRAS* double aberrations, and 1.95% (24/1228) of patients harbor *PIK3CG* and *KRAS* double aberrations [35]. Moreover, no patients were found to carry the same specific *PIK3CA* and *KRAS* mutation in the cell line. For patients with the same gene aberration, further experiments are still needed to verify the efficacy of the inhibitor.

We also identified that in the tested ten PDAC cell lines, all carry only one *TP53* variant that can cause amino acid or RNA structure changes, except SU.86.86, which carries two *TP53* variants. It has been reported that patients with advanced PDAC who have two *TP53* mutations and who were treated with the EGFR-inhibitor Erlotinib demonstrated rapid disease progression, which suggests that multiple *TP53* mutations reduce the efficacy of specific inhibitors against PDAC [39]. In our study, a comprehensive analysis of the cell viability assays and the number of *TP53* variants revealed that SU.86.86 is in the low-sensitivity group when testing both inhibitors, suggesting that two *TP53* mutations are related to reducing the efficacy of PI3K/AKT pathway inhibitors (Figure 6). Therefore, when multiple *TP53* mutations are identified, the combination of inhibitors and drugs should be considered.

## 4. Materials and Methods

### 4.1. Kinase Inhibitors

The kinase inhibitors Buparlisib (Pan-PI3K inhibitor) and MK-2206 (AKT1/2/3 inhibitor) were purchased from Selleck Chemicals (Absource Diagnostics GmbH, Munich, Germany). According to the manufacturer’s instructions, Buparlisib and MK-2206 were separately dissolved in dimethyl sulfoxide (DMSO) (Sigma-Aldrich Chemie GmbH, Steinheim, Germany) as a stock solution at a final concentration of 10 mM. The stock solutions were stored at −80 °C and diluted into corresponding working concentrations before each experiment.

### 4.2. Cell Lines and Cell Culture

PDAC cell lines AsPc-1, BxPc-3, Capan-1, Colo357, Panc-1, PaTu8902, PaTu8988T, PaTu8988S, SU.86.86, and T3M4 were kindly provided by the University of Greifswald. AsPc-1, BxPc-3, Colo357, Panc-1, SU.86.86, and T3M4 were cultured in RPMI1640 medium (PAN-Biotech, Aidenbach, Germany) supplemented with 10% heat-inactivated fetal calf serum (FCS) (PAN-Biotech, Aidenbach, Germany) and 1% Penicillin-Streptomycin (P/S) solution (10,000 U/mL Penicillin, 10 mg/mL Streptomycin) (PAN-Biotech, Aidenbach, Germany). PaTu8902, PaTu8988T, and PaTu8988S were cultured in DMEM/F12 medium (PAN-Biotech, Aidenbach, Germany) supplemented with 10% heated-inactivated FCS and 1% P/S solution. Capan-1 was cultured in RPMI1640 medium supplemented with 15% heat-inactivated FCS and 1% P/S solution. After verifying that all cell lines were not contaminated by mycoplasma, these PDAC cell lines were maintained in a 5% CO_2_ humidified atmosphere incubator at 37 °C.

For all assays, the PDAC cell lines were seeded at a density of 3.3 × 10^4^ cells per milliliter in 6-well plates (totaling 4.5 mL per well), 24-well plates (totaling 1.5 mL per well), and 96-well plates (totaling 150 μL per well). After 24 h, the supernatant was discarded, and media containing increasing concentrations (range from 1 μM–10 μM for MK-2206 and 0.5 μM–10 μM for Buparlisib) of the inhibitors or vehicle (DMSO, as control) were added to the corresponding PDAC cell lines. The treated cells were incubated for up to 72 h at 37 °C with 5% CO_2_. At the indicated time points, cell proliferation, metabolic activities, cell biomass, and apoptosis/necrosis were evaluated in at least three biologically independent replicates.

### 4.3. Cell Viability Assays

#### 4.3.1. Proliferation

Cell proliferation was evaluated by absolute cell counting and trypan blue (Sigma-Aldrich Chemie GmbH, Steinheim, Germany) staining. After inhibitor exposure in 24-well plates, the cells were harvested and washed with 1× PBS (PAN-Biotech, Aidenbach, Germany). In the following step, the cells were stained with trypan blue, and the numbers of viable cells were determined by counting with a hemocytometer. Proliferation was expressed as the percentage of viable cells treated with the inhibitor compared to the 100% DMSO control.

#### 4.3.2. Metabolic Activity

Metabolic activity was tested by Water Soluble Tetrazolium—1 (WST-1) (TaKaRa Bio Inc., Kusatsu, Japan). After exposure to the corresponding inhibitor, the cells were incubated with 15 μL WST-1 for up to 2 h in 96-well plates. Absorbances at 450 nm and the reference wavelength of 620 nm were measured by Promega GloMax^®^-Multi Microplate Multimode Reader (Promega, Madison, WI, USA). The metabolic activity was calculated as recommended by the manufacturer. Metabolic activity is expressed as a percentage of the inhibitor-treated group compared to vehicle-treated controls (control = 100%).

#### 4.3.3. Biomass Quantification

Biomass quantification was carried out by Crystal Violet (CV) (Sigma-Aldrich GmbH, Steinheim, Germany) staining. After exposure to the corresponding inhibitor in 96-well plates, the cells were washed once with PBS and stained with 50 μL of 0.2% CV solution on a shaker at room temperature for 10 min. Thereafter, the plates were washed twice with PBS. To elute bound CV, 100 μL 1% sodium dodecyl sulfate (SDS) (SERVA Electrophoresis GmbH, Heidelberg, Germany) was added to each well and incubated on a shaker at room temperature for 10 min. Finally, absorbances at 570 nm and a reference wavelength at 620 nm were measured by Promega GloMax^®^-Multi Microplate Multimode Reader. For background normalization, the absorbance of each group was subtracted from the absorbance of pure culture media. The amount of CV directly correlates to the cell biomass. The result is expressed as a percentage of the inhibitor-treated group compared to vehicle-treated controls (control = 100%).

### 4.4. Identification of IC50

IC50 values were calculated independently based on cell proliferation, metabolic activity, or biomass after 72 h of inhibitor exposure. GraphPad Prism Version 8.0.2 (GraphPad Software Inc., San Diego, CA, USA) was used to evaluate IC50. Briefly, after transforming concentrations and normalizing the results for the three vitality assays, a nonlinear regression model (dose-response-inhibition vs. normalized response—variable slope) was used to evaluate the IC50 values. We calculated the IC50 corresponding to the three vitality assays and applied these results to a response-based clustering analysis in order to evaluate the sensitivity of the cell lines to inhibitors.

### 4.5. Apoptosis and Necrosis Analyses

Apoptosis and necrosis were evaluated by YO-PRO-1 (Invitrogen, Darmstadt, Germany) and Propidium iodide (PI) (Sigma-Aldrich GmbH, Steinheim, Germany) double staining by flow cytometry. After exposure to the corresponding inhibitor, supernatants were collected, and cells were harvested and washed twice with cold PBS. Following that, cells were resuspended in 200 μL YO-PRO-1 (final concentration: 0.2 μM) solution. After incubating at room temperature for 20 min in the dark, cells were washed twice in cold PBS and resuspended in 400 μL cold PBS. Cells were then stained with PI (final concentration: 100 μg/mL) immediately before measurement. Unstained and single-stained cells were used as controls and measured in every single experiment. YO-PRO-1^−^/PI^−^ cells are considered viable cells, YO-PRO-1^+^/PI^−^ cells are considered apoptotic cells, and PI^+^ cells are considered necrotic cells. Flow cytometry measurements were performed on FACSVerse (Becton, Dickinson and Company, Heidelberg, Germany), and data were analyzed by BD FlowJo software (Becton, Dickinson and Company, Heidelberg, Germany).

### 4.6. Nucleic Acid Extraction

Genomic DNA was extracted by NucleoSpin^®^ Tissue Kit (MACHEREY-NAGEL GmbH, Dueren, Germany) according to the manufacturers’ instructions. In brief, 5 × 10^6^ cells were harvested from each continuous cultural cell line and washed twice with cold sterile PBS. Cell pellets were lysed, and then the lysis that contained genomic DNA was extracted and purified by a silica membrane of NucleoSpin column. Lastly, genomic DNA was eluted with 30 μM of nuclease-free water.

Total RNAs were extracted by miRNeasy Mini Kit (QIAGEN GmbH, Hilden, Germany) according to the manufacturers’ instructions. In brief, 5 × 10^6^ cells were harvested from each continuous cultural cell line and washed twice with cold sterile PBS. Cell pellets were resuspended in 700 μL QIAzol Lysis Reagent (QIAGEN GmbH, Hilden, Germany), and then the aqueous phase that contains the total RNA of the lysed cells was extracted and purified by a silica membrane of RNeasy Mini spin columns. Finally, total RNA was eluted in 30 μL of nuclease-free water.

After extraction, nucleic acid concentrations, as well as OD260/280 and OD260/230 ratios, were measured with a NanoDrop 1000 Spectrophotometer (Thermo Fisher Scientific Inc., Waltham, MA, USA).

### 4.7. Whole-Exome Sequencing

Barcoded sequencing libraries were generated after enrichment with the SureSelect Human All Exon kit (Agilent, Santa Clara, CA, USA), pooled and sequenced on a HiSeq4000 (Illumina Inc., San Diego, CA, USA) instrument using a 150 paired-end protocol to yield at least 20× coverage for >98% of the target region and an overall average depth of coverage above 100×. An in-house bioinformatics pipeline was used, including read alignment to human genome reference hg 19, variant calling (single nucleotide substitutions and small deletions/insertions), and variant annotation with publicly available databases.

### 4.8. Variant Calling Filtering Strategy

**Figure 7:**
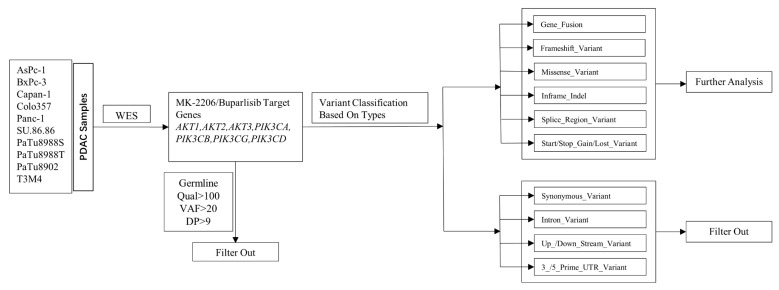
Filtering strategy of MK-2206 and Buparlisib target genes.

After WES, the sequencing data from ten PDAC cell lines were obtained and filtered in order to select variants with the expected highest impact on gene function. Briefly, variants were filtered based on quality (qual), variant allele frequency (VAF), depth of coverage (DP), and variant type. In order to exclude false positive variants, only variants with qual > 100, VAF > 20, and DP > 9 were included in our analysis. Germline mutations were excluded by comparison with COSMIC and dbSNP databases. Then, variant types were excluded if they were unable to cause amino acid substitution, RNA structure change, or base insertions/deletions (indels). These variant types include synonymous variants, intron variants, upstream or downstream variants, and 3 prime or 5 prime untranslated region (UTR) variants. After this filtering procedure, missense variants, splice region variants, inframe indels, frameshift variants, gene fusion, and start/stop gain or lost were chosen for further analysis (Figure 7).

### 4.9. Gene Expression Analyses

Barcoded sequencing libraries were prepared with the TruSeq Stranded mRNA kit (Illumina Inc., San Diego, CA, USA), pooled and sequenced on a NextSeq 500 System (Illumina Inc., San Diego, CA, USA) using the 75 bp paired-end protocol. At least 30 million reads were obtained for each sample. The reads were aligned to reference genome GRCh37/Release 38 with STAR V.2.7.6a using the two-pass mode [40]. Transcript abundance and transcript per million estimates were calculated by counting the reads using featureCounts/subread V.2.0.1 [41].

The expression data of non-neoplastic pancreatic tissue from The Genotype-Tissue Expression (GTEx) and the Cancer Genome Atlas Program (TCGA) were chosen as controls.

### 4.10. Response-Based Clustering Strategy

The classification of cell lines into distinct sensitivity levels was performed by k-means++ clustering based on an unsupervised machine learning algorithm [42]. Briefly, cell proliferation, metabolic activity, and biomass were analyzed after treating the cells with various inhibitor concentrations and calculating the IC50 values. Then, all IC50 values were collected and applied to the Sci-kit learn package using the Python programming language to predict optimal clusters. The Silhouette score was used to detect the clustering density and the separation between the clusters. Ten cell lines were set to be divided into several clusters, and the cluster grouping was iterated a maximum of 100 times to test for the robustness of the classification. Finally, the ten cell lines were divided into different clusters identified as high, moderate, and low sensitivity groups based on their biological characteristics.

### 4.11. Statistical Analyses

Data have been replicated with at least three biologically independent experiments. GraphPad Prism Version 8.0.2 was used for statistical analysis. The results of proliferation, metabolic activity, biomass quantification, and apoptosis/necrosis analysis were expressed as mean ± standard deviation (SD). Statistical significance was determined by one-way ANOVA (after proving that the data within each group conformed to the Gaussian distribution) or the Kruskal-Willas-Test (for the data within each group that conformed to a non-Gaussian distribution) and displayed as *: *p* < 0.033, **: *p* < 0.002, ***: *p* < 0.001 versus the control group.

## 5. Conclusions

Our present study reveals distinct antitumor effects against PDAC cell lines when inhibiting the PI3K/AKT pathway. Exploring the inhibitor response and the corresponding target gene aberrations shows that neither *PIK3CA* nor *PIK3CG* aberration alone affect the inhibitor response of PDAC cell lines to Buparlisib or MK-2206. Moreover, in the relationship between the observed inhibitor response and aberrations of *KRAS* and *TP53*, *KRAS* point mutations (c.35C > T, c.35C > A, and c.183A > C) alone are not able to determine the level of sensitivity to Buparlisib, but they do appear to be related to the level of sensitivity to MK-2206. Cell line carrying a specific *PIK3CA* variant is associated with enhanced Buparlisib inhibition in *KRAS*-mutated cell lines. In addition, carrying two *TP53* missense variants appears to be associated with reduced sensitivity to PI3K/AKT pathway inhibitors. Thus, our study suggests that blocking the PI3K/AKT pathway is an optional strategy for the treatment of patients with PDAC but that it is still necessary to choose inhibitors based on genetic background.

## Data Availability

The data supporting the reported results can be found on the website listed in the article.

## Supplementary Materials

The following are available online at https://www.mdpi.com/article/10.3390/ijms23084295/s1.

## Abbreviations

Abbreviation	Meaning
AKT, AKT	Protein kinase B
Ala	Alanine
Arg	Arginine
Asp	Aspartate
CV	Crystal violet
Cys	Cysteine
DMSO	Dimethyl sulfoxide
DP	Reading depth
FCS	Fetal calf serum
fs	Frameshift
Gln	Glutamine
Gly	Glycine
GTEx	The Genotype-Tissue Expression
His	Histidine
IC50	Half maximal inhibitory concentration
indel	Insertion/deletion
*KRAS*, KRAS	Kirsten rat sarcoma viral oncogene homolog
mTOR	Mammalian target of rapamycin
NF-κB	Nuclear factor kappa-light-chain-enhancer of activated B cells
OD	Optical Density
PBS	Phosphate buffer saline
PDAC	Pancreatic ductal adenocarcinoma
PI	Propidium iodide
PI3K	Phosphatidylinositol-4,5-bisphosphate 3-kinase
P/S	Penicillin/streptomycin
qual	Variant confidence
RNA-seq	RNA sequencing
SD	Standard deviation
Ser	Serine
TCGA	The Cancer Genome Atlas Program
Thr	Threonine
*TP53*, p53	Tumor protein p53
TPM	Transcript per kilobase million
Trp	Tryptophan
Tyr	Tyrosine
VAF	Variant allele frequency
Val	Valine
UTR	Untranslated region
WES	Whole exome sequencing
WST-1	Water soluble tetrazolium—1
