# Supplementary material for: The Inhibitory Response to PI3K/AKT Pathway Inhibitors MK-2206 and Buparlisib Is Related to Genetic Differences in Pancreatic Ductal Adenocarcinoma Cell Lines
Source: Int J Mol Sci. 2022 Apr 13;23(8):4295. doi: 10.3390/ijms23084295 (PMC9029322; doi:10.3390/ijms23084295)
Supplement: Supplementary file 1 [file ijms-23-04295-s001.zip › Supplementary Figures.pdf]

Supplementary Figure S1

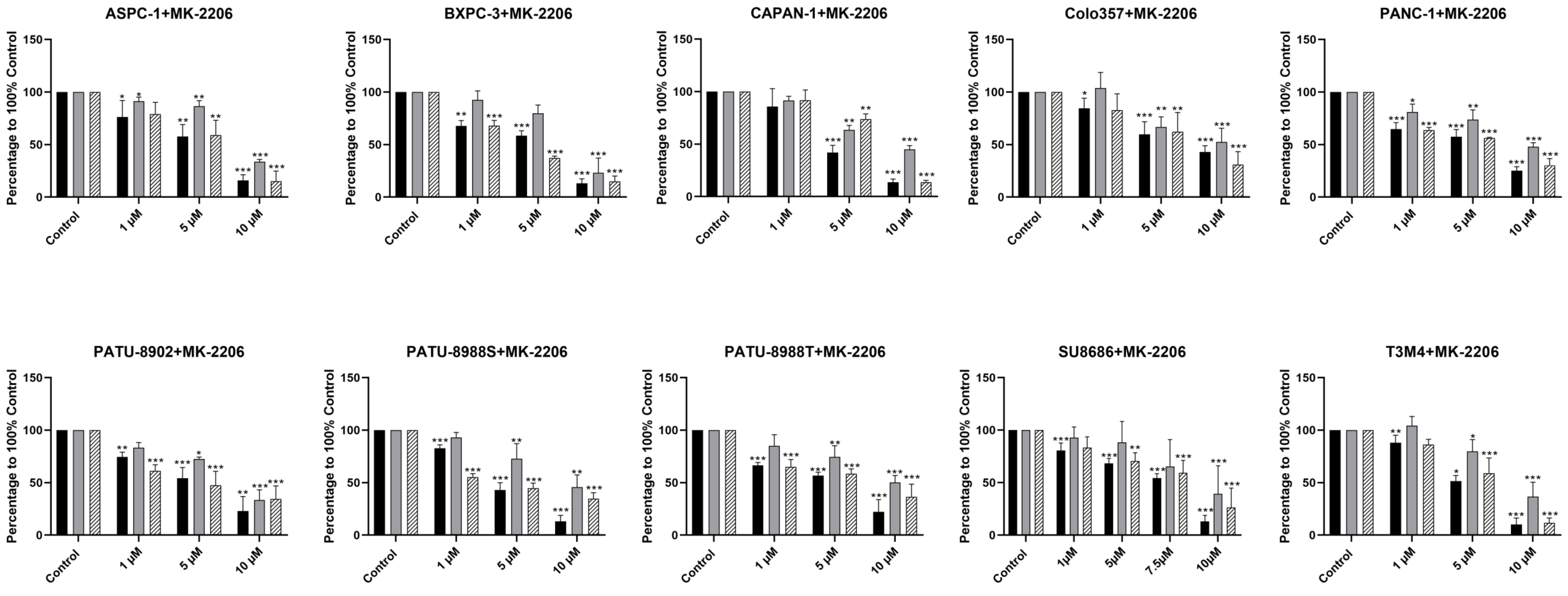

Figure S1. Cell viability after 72 hours MK-2206 exposure in ten PDAC cell lines. Data are presented as Mean ± SD. Significance of a treatment effect (which showed in bar charts) compared to the DMSO control was determined by one-way ANOVA or Kruskal-Wallis-Test and displayed as *: $P< 0.033$, **: $P< 0.002$, ***: $P<0.001$ (n ≥ 3).

Supplementary Figure S2

a:

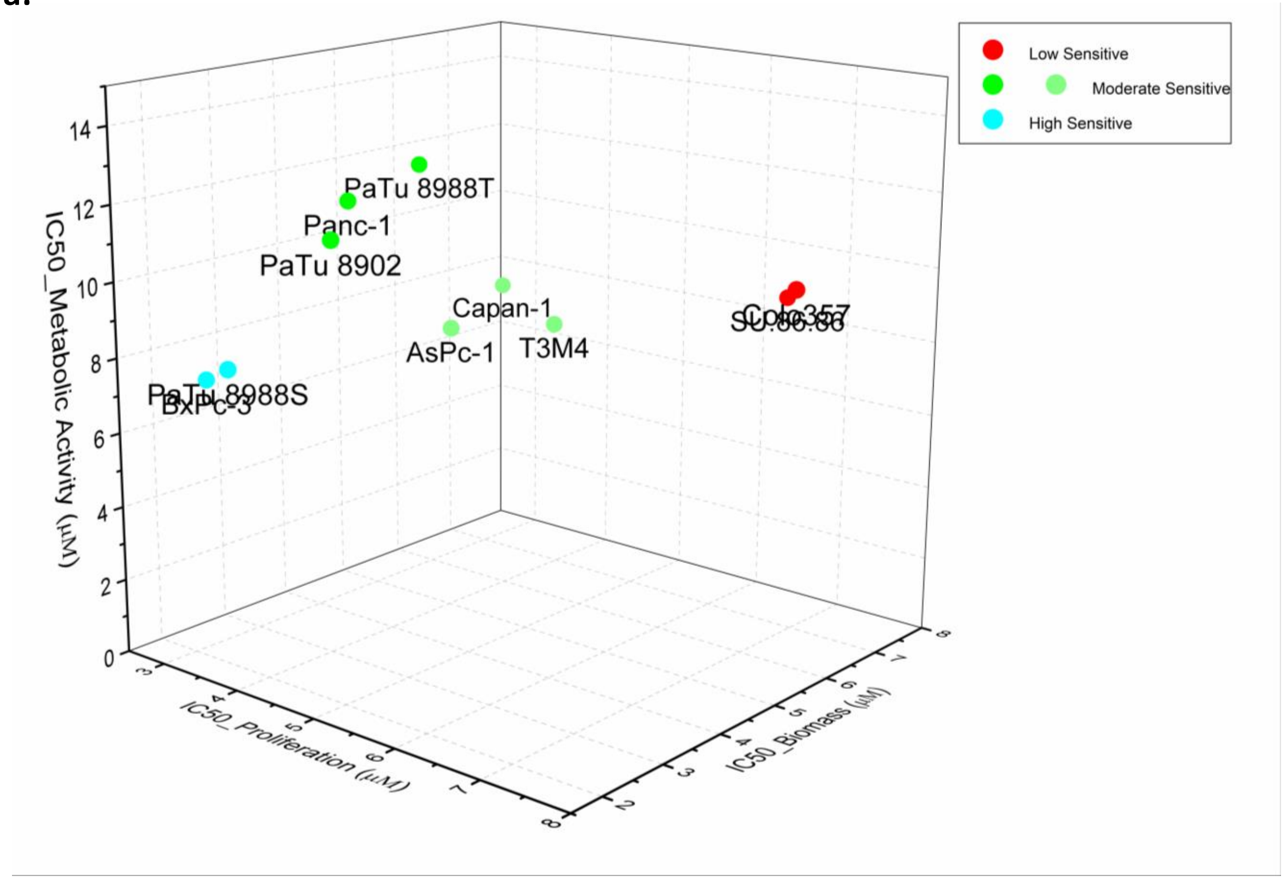

b:

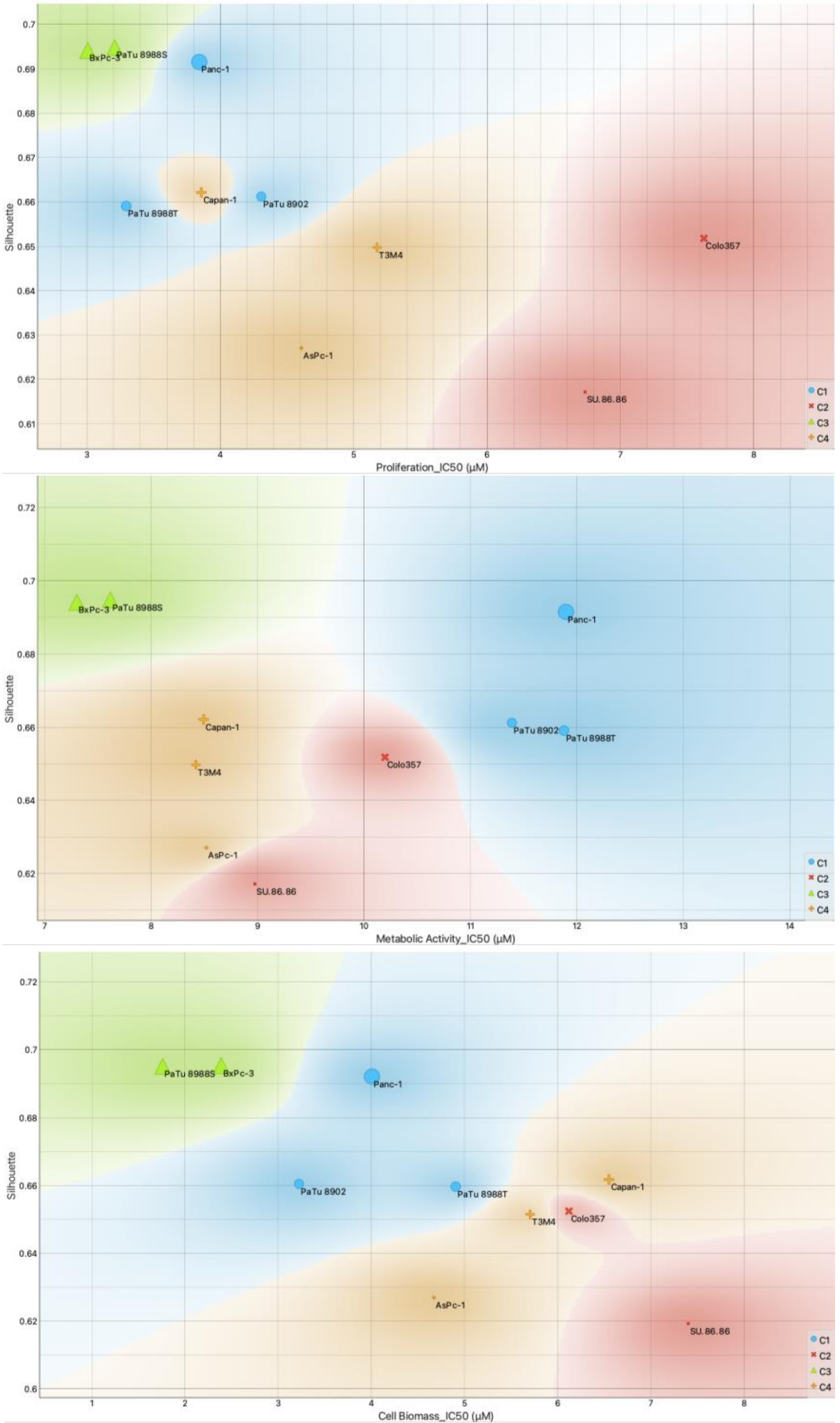

Figure S2. 3d(a) and 2d(b) clustering results after 72 hours of exposure to MK-2206 in ten PDAC cell lines. In figure (a), k-means++ (unsupervised machine learning algorithm) was used to determine the low sensitivity (red), moderate sensitivity (green), and high sensitivity (blue) groups. In figure (b), from top to bottom are the results of cell proliferation, metabolic activity, and biomass. The X axis represents the IC50 calculated according to cell proliferation, metabolic activity, and biomass, respectively. The Y axis represents the silhouette score generated from k-means++. The different colors in figure (b) only represent the results of clustering. In the end, the ten cell lines were clustered into four groups. However, comprehensive 3d and 2d cluster analysis, the biological characteristics of the C1 and C4 groups are the closest, so they are grouped into one, finally three sensitivity groups are determined.

Supplementary Figure S3

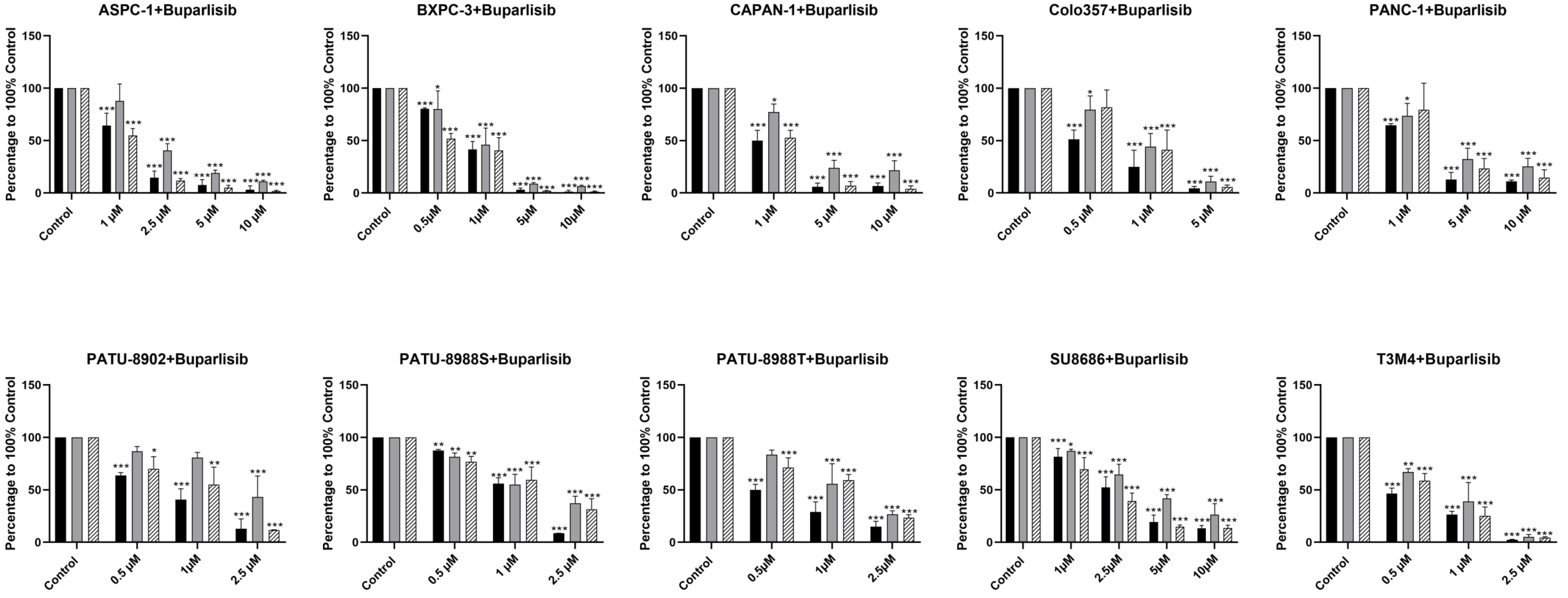

Figure S3. Cell viability after 72 hours Buparlisib exposure in ten PDAC cell lines. Data are presented as Mean $\pm$ SD. Significance of a treatment effect (which showed in bar charts) compared to the DMSO control was determined by one-way ANOVA or Kruskal-Wallis-Test and displayed as *: P< 0.033, **: P< 0.002, ***: P<0.001 (n ≥ 3).

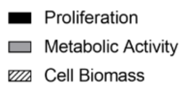

Supplementary Figure S4

a:

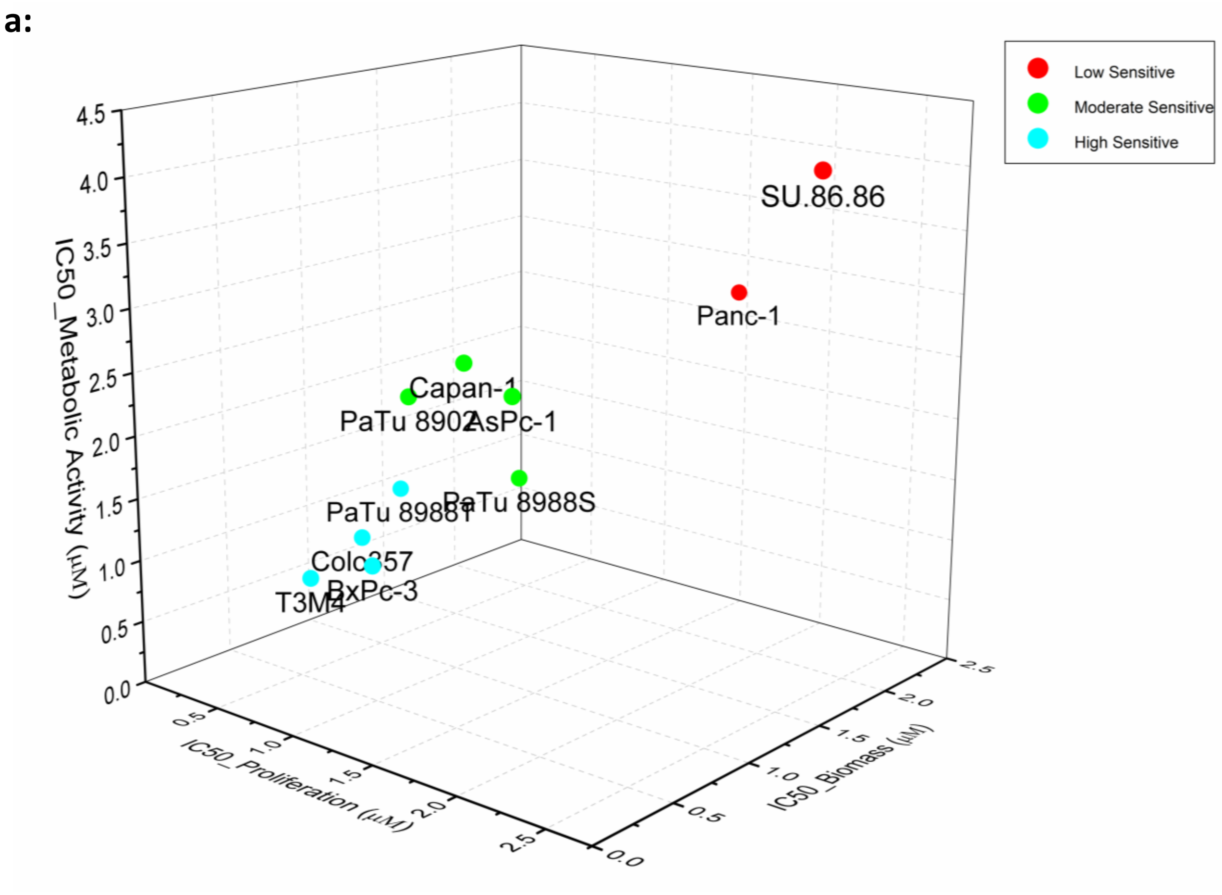

b:

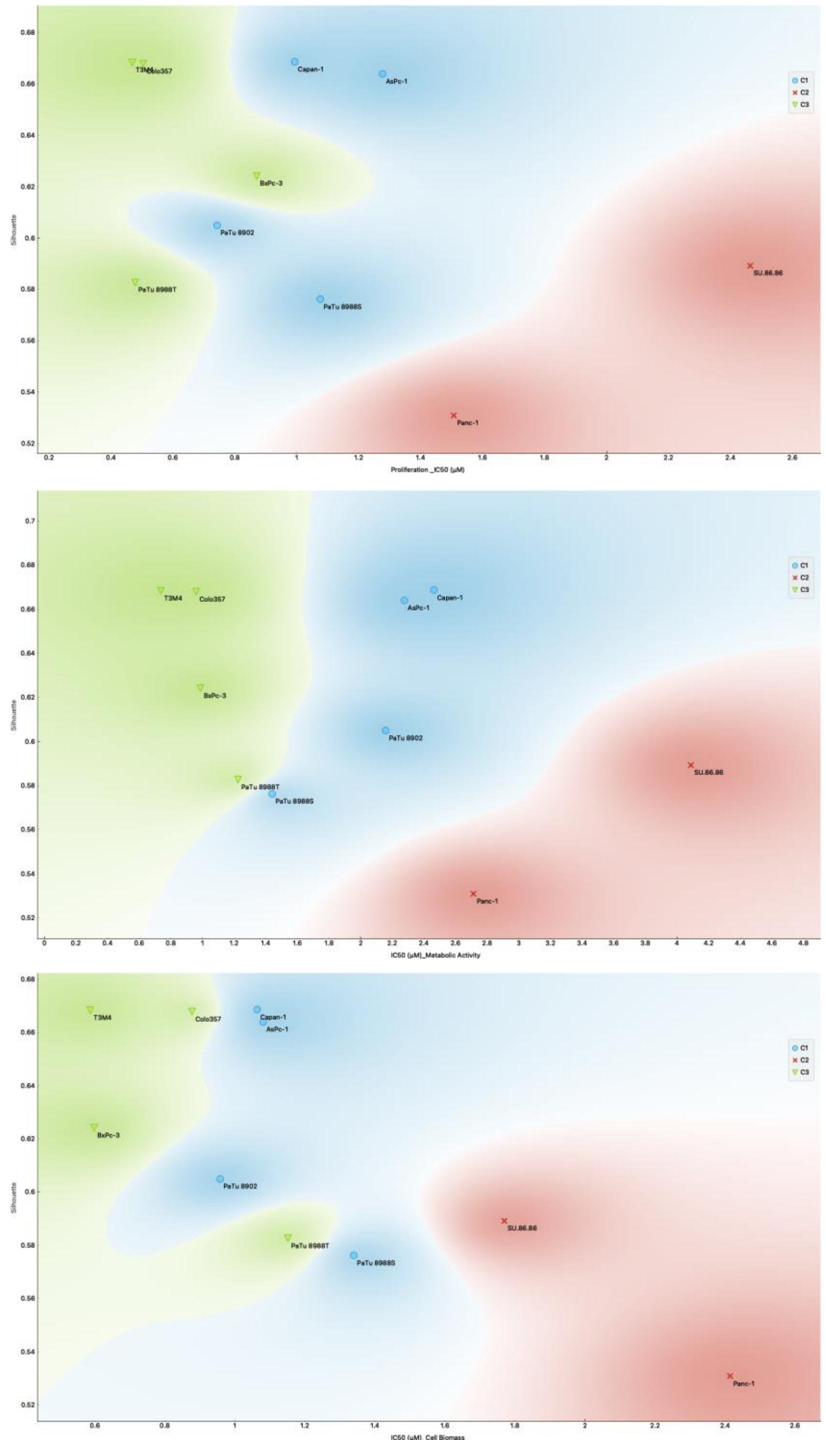

Figure S4. 3d(a) and 2d(b) clustering results after 72 hours of exposure to Buparlisib in 10 PDAC cell lines. In figure (a), k-means++ (unsupervised machine learning algorithm) was used to determine the low sensitivity (red), moderate sensitivity (green), and high sensitivity (blue) groups. In figure (b), from top to bottom are the results of cell proliferation, metabolic activity, and biomass clustering. The X axis represents the IC50 calculated according to cell proliferation, metabolic activity, and biomass, respectively. The Y axis represents the silhouette score generated from k-means++. The different colors in figure (b) only represent the results of k-mean++ clustering. The ten PDAC cell lines were clustered into 3 groups. Comprehensive 3d and 2d cluster analysis finally determined three sensitivity groups.

Supplementary Figure S5

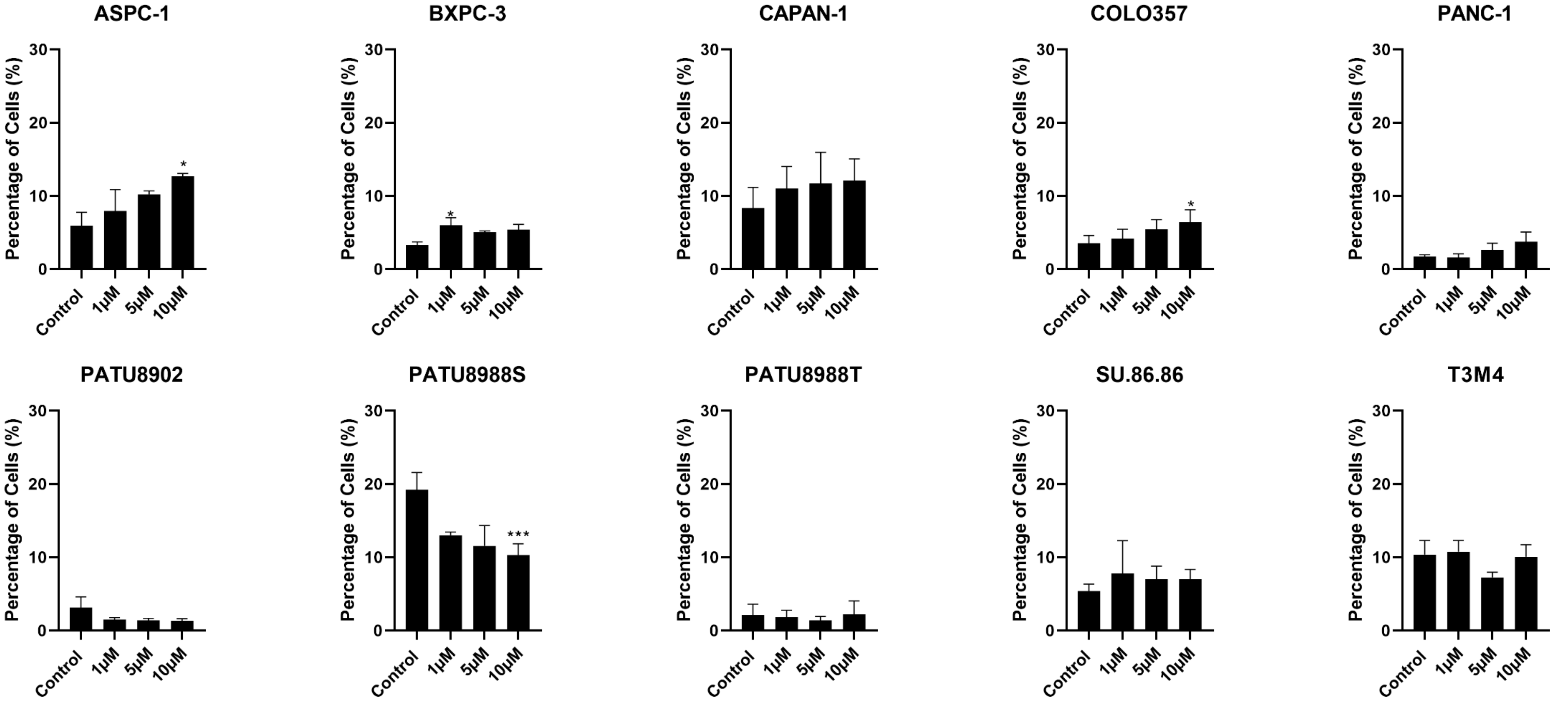

Figure S5. Apoptosis/necrosis induction in ten PDAC cell lines after 72h MK-2206 exposure. Induction of apoptosis/necrosis was determined by flow cytometry after YO-PRO-1 FITC and propidium iodide (PI) staining . As a reference, DMSO treated cells were analyzed. Percentages of apoptotic (YO-PRO-1+, PI-) and necrotic (PI+) cells were determined and displayed as the Mean ± SD of more than three independent measurements. Significance of a treatment effect compared to the DMSO control was determined by one-way ANOVA or Kruskal-Wallis-Test and displayed as *: P< 0.033, **: P< 0.002, ***: P<0.001 (n ≥ 3).

Supplementary Figure S6

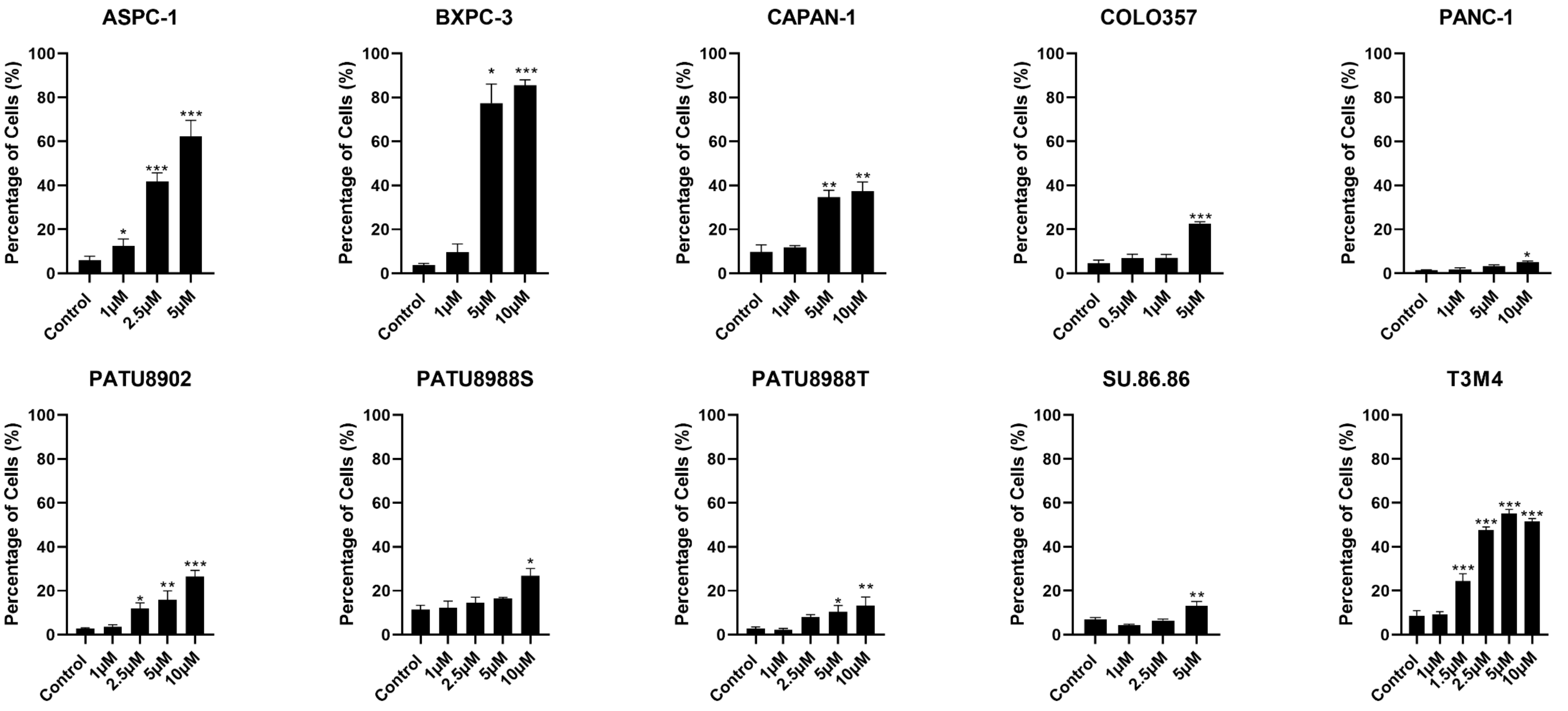

Figure S6. Apoptosis/necrosis induction in ten PDAC cell lines after 72h Buparlisib exposure. Induction of apoptosis/necrosis was determined by flow cytometry after YO-PRO-1 FITC and propidium iodide (PI) staining . As a reference, DMSO treated cells were analyzed. Percentages of apoptotic (YO-PRO-1+, PI-) and necrotic (PI+) cells were determined and displayed as the Mean ± SD of more than three independent measurements. Significance of a treatment effect compared to the DMSO control was determined by one-way ANOVA or Kruskal-Wallis-Test and displayed as *: P< 0.033, **: P< 0.002, ***: P<0.001 (n ≥ 3).

Supplementary Figure S7 – Apoptosis/Necrosis Dot Plot – MK-2206 (1)

ASPC-1

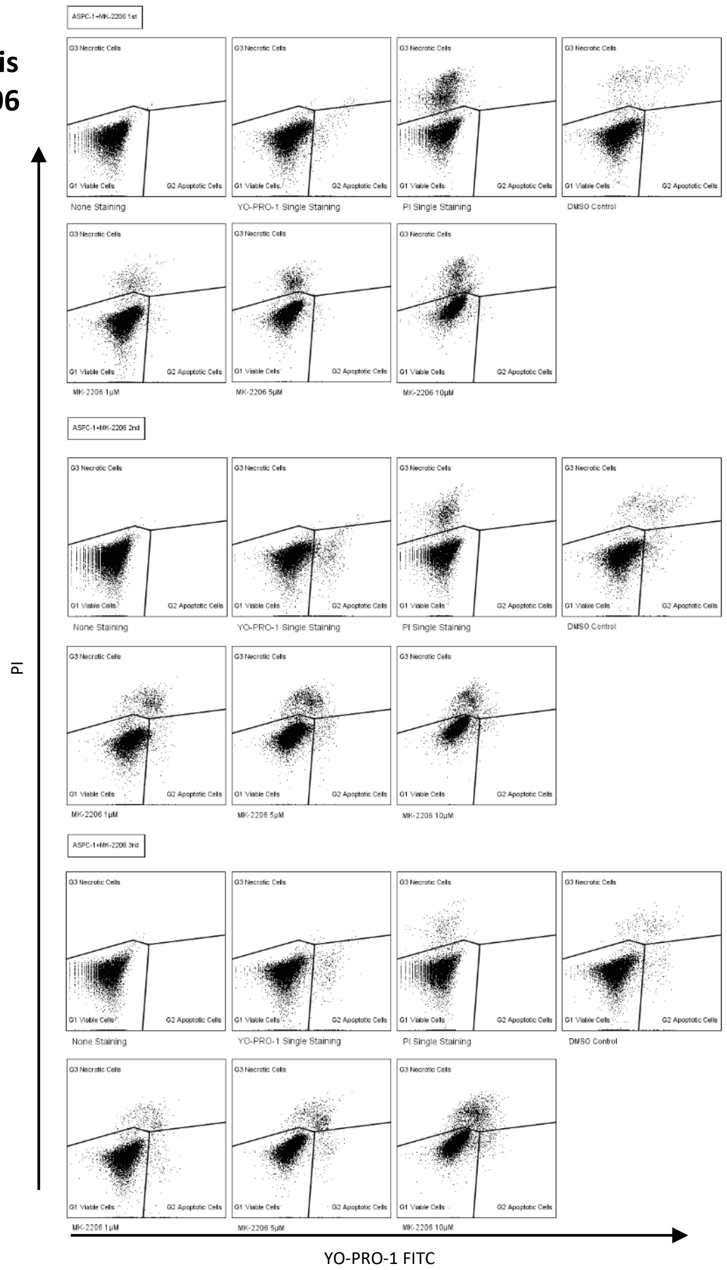

	1st		2nd		3rd		Mean	SD	P-Value (vs. Control)
Concentration	G2(YP⁺/PI⁻)	G3(PI⁺)	G2(YP⁺/PI⁻)	G3(PI⁺)	G2(YP⁺/PI⁻)	G3(PI⁺)	G2+G3		
Control	0.63	4.30	3.45	5.06	1.89	2.49	5.94	1.83	
1μM	0.73	5.17	3.15	8.91	2.47	3.38	7.94	2.92	>0.99
2.5μM	0.41	9.49	1.62	9.27	2.03	7.85	10.22	0.47	0.42
5μM	0.11	12.6	1.01	11.2	1.73	11.4	12.68	0.38	0.01

BXPC-3

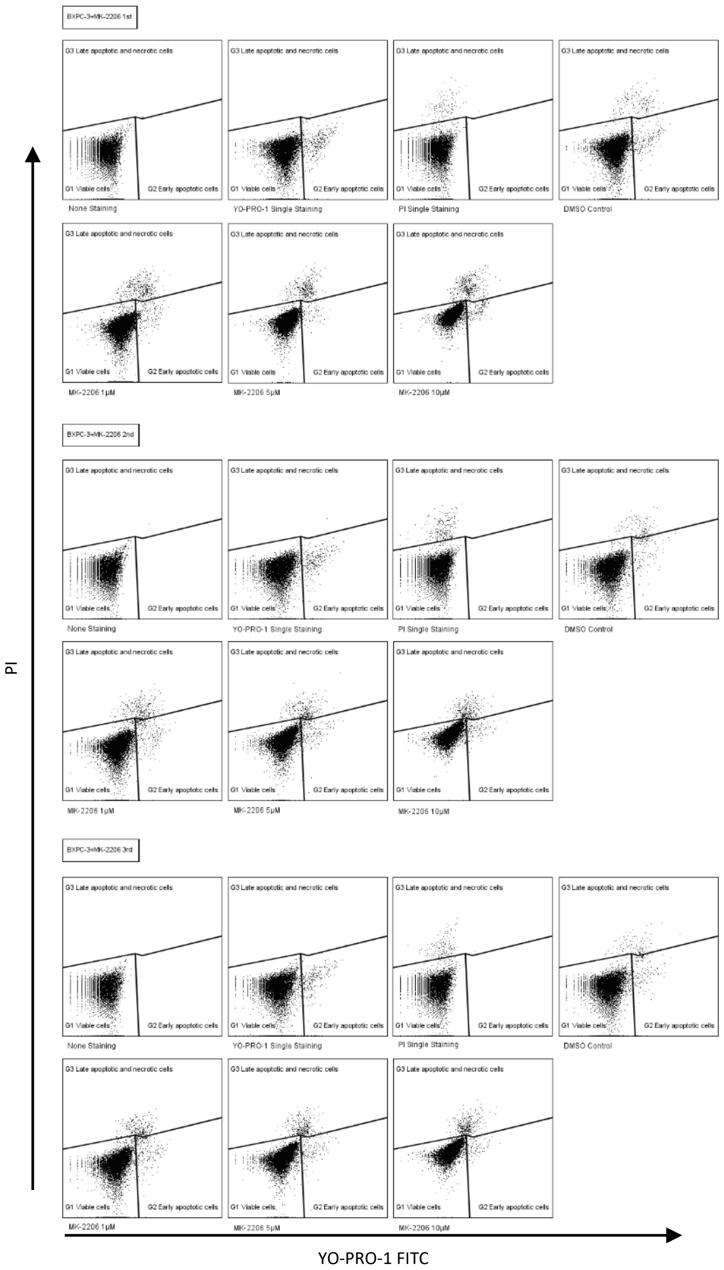

	1st		2nd		3rd		Mean	SD	P-Value (vs. Control)
Concentration	G2(YP⁺/PI⁻)	G3(PI⁺)	G2(YP⁺/PI⁻)	G3(PI⁺)	G2(YP⁺/PI⁻)	G3(PI⁺)	G2+G3		G2+G3
Control	2.25	1.66	1.12	1.82	1.41	1.55	3.27	0.45	
1μM	2.87	4.61	1.72	3.60	1.68	3.40	5.96	1.08	0.02
5μM	1.02	3.81	1.61	3.66	1.34	3.60	5.01	0.19	0.34
10μM	1.45	4.95	1.05	3.70	0.94	4.05	5.38	0.73	0.21

Supplementary Figure S7 – Apoptosis/Necrosis Dot Plot – MK-2206 (2)

CAPAN-1

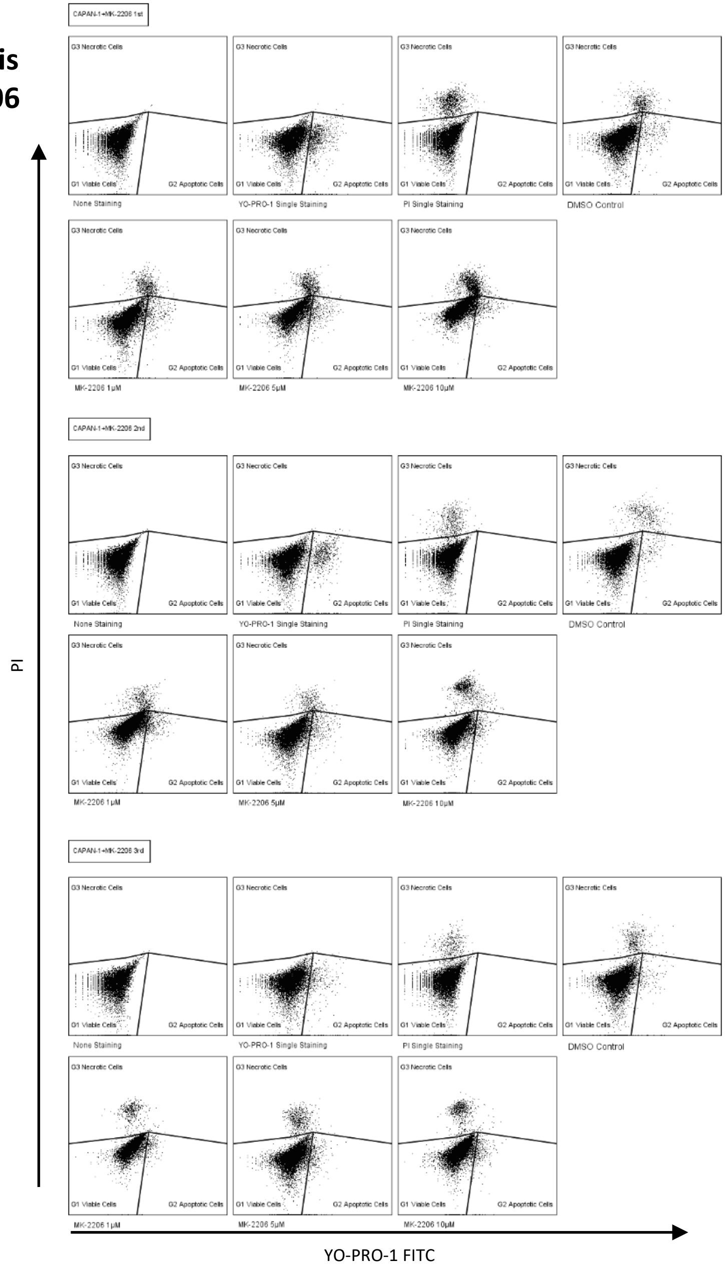

	1st		2nd		3rd		Mean	SD	P-Value (vs. Control)
Concentration	G2(YP+/PI-)	G3(PI+)	G2(YP+/PI-)	G3(PI+)	G2(YP+/PI-)	G3(PI+)	G2+G3		G2+G3
Control	6.60	5.70	1.75	4.94	1.68	4.34	8.34	2.82	
1μM	7.55	7.42	3.88	3.90	3.61	6.70	11.02	2.98	0.76
5μM	7.62	9.46	2.54	4.05	3.72	7.64	11.68	4.29	0.64
10μM	4.51	11.3	2.52	9.40	2.24	6.27	12.08	2.98	0.56

Supplementary Figure S7 – Apoptosis/Necrosis Dot Plot – MK-2206 (3)

COLO357

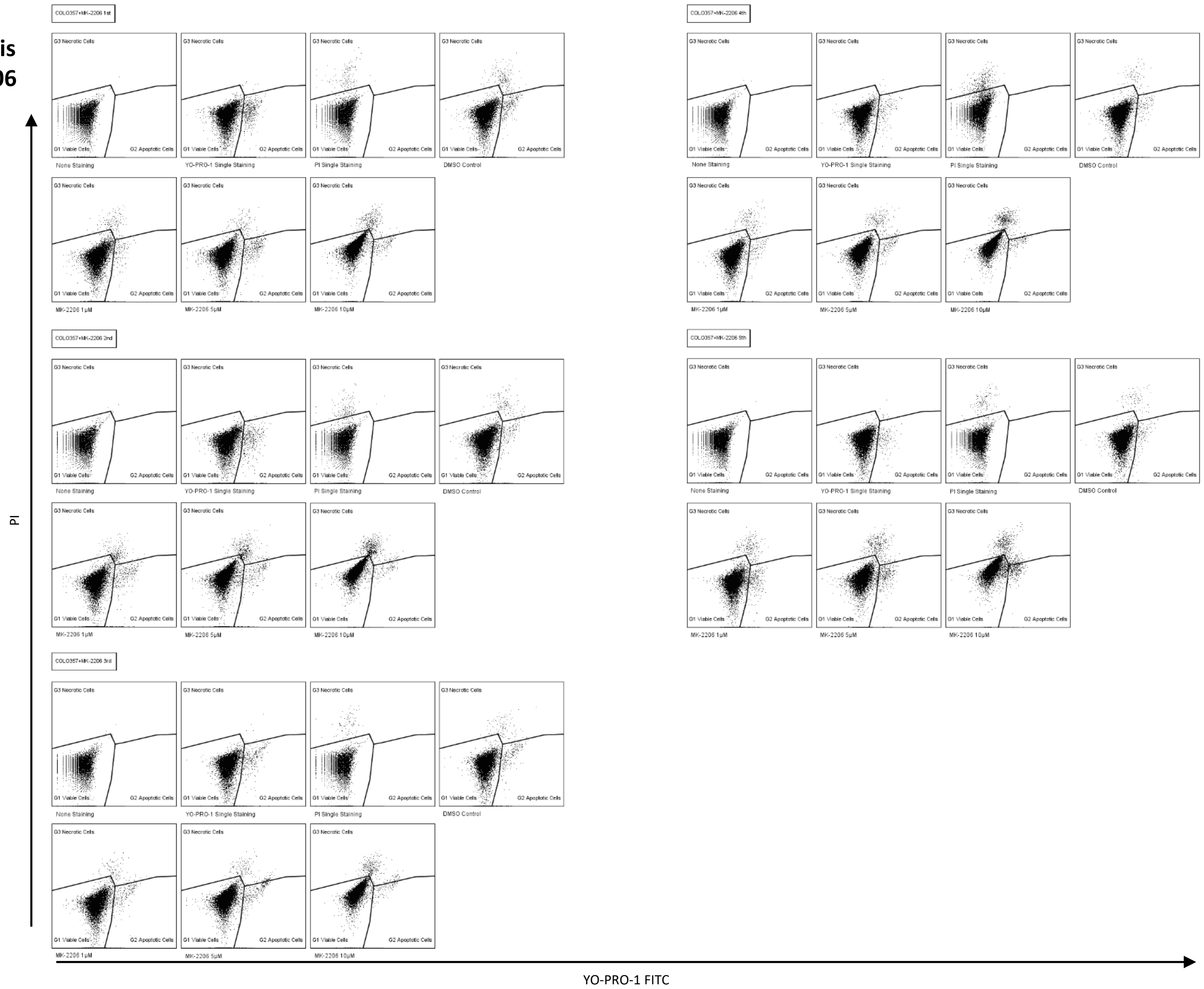

	1st		2nd		3rd		4th		5th		Mean	SD	P-Value (vs. Control)
Concentration	G2(YP⁺/PI⁻)	G3(PI⁺)	G2(YP⁺/PI⁻)	G3(PI⁺)	G2(YP⁺/PI⁻)	G3(PI⁺)	G2(YP⁺/PI⁻)	G3(PI⁺)	G2(YP⁺/PI⁻)	G3(PI⁺)	G2+G3		
Control	3.05	2.17	2.13	1.42	2.87	1.11	1.24	0.90	1.70	1.00	3.52	1.07	
1μM	1.58	1.6	3.46	2.45	2.35	1.01	1.22	1.69	3.31	2.17	4.17	1.26	0.84
5μM	3.81	1.16	2.72	3.84	3.21	1.39	1.67	2.05	3.69	3.65	5.44	1.32	0.15
10μM	1.73	3.32	1.44	5.91	1.66	2.82	1.30	4.77	4.09	5.10	6.43	1.69	0.02

Supplementary Figure S7 – Apoptosis/Necrosis Dot Plot – MK-2206 (4)

PANC-1

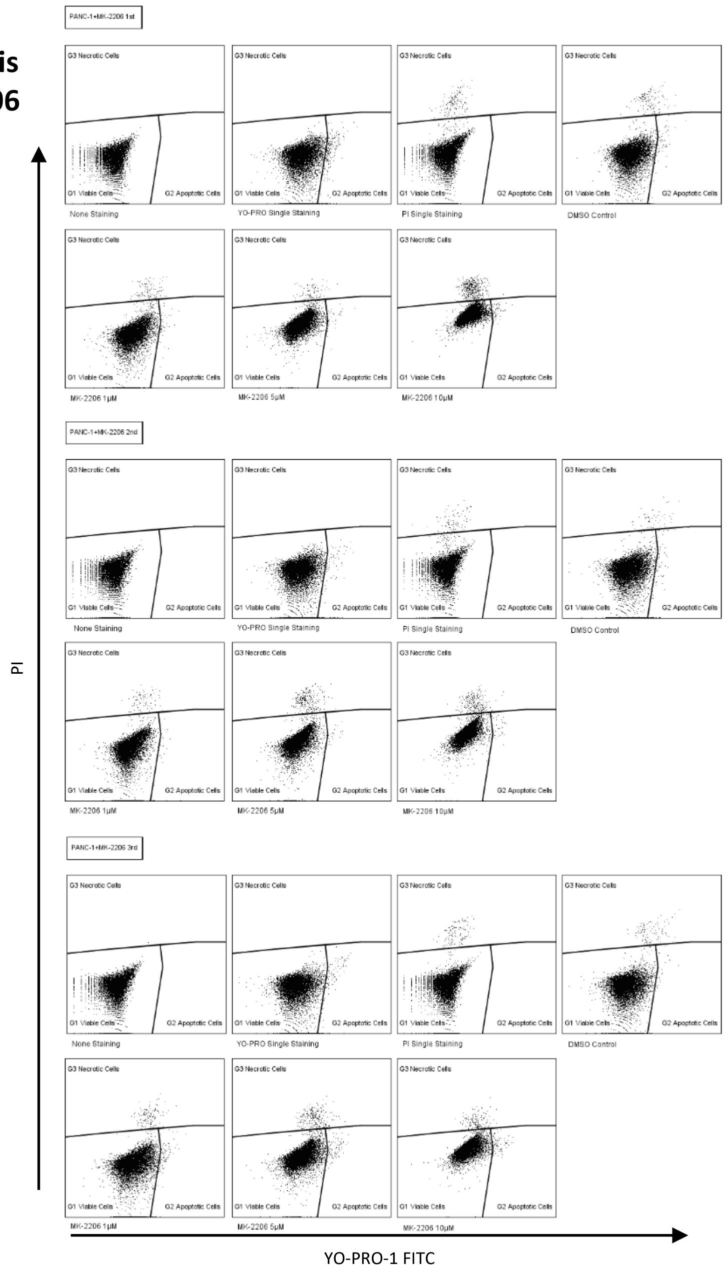

	1st		2nd		3rd		Mean	SD	P-Value (vs. Control)
Concentration	G2(YP+/PI-)	G3(PI+)	G2(YP+/PI-)	G3(PI+)	G2(YP+/PI-)	G3(PI+)	G2+G3		G2+G3
Control	0.76	1.27	1.1	0.55	0.34	1.12	1.71	0.24	
1μM	0.35	1.03	0.27	0.86	0.92	1.35	1.59	0.49	>0.99
5μM	0.36	0.99	0.36	2.31	0.98	2.73	2.58	0.97	0.65
10μM	0.58	4.97	0.42	2.78	0.83	1.59	3.72	1.33	0.11

PATU8902

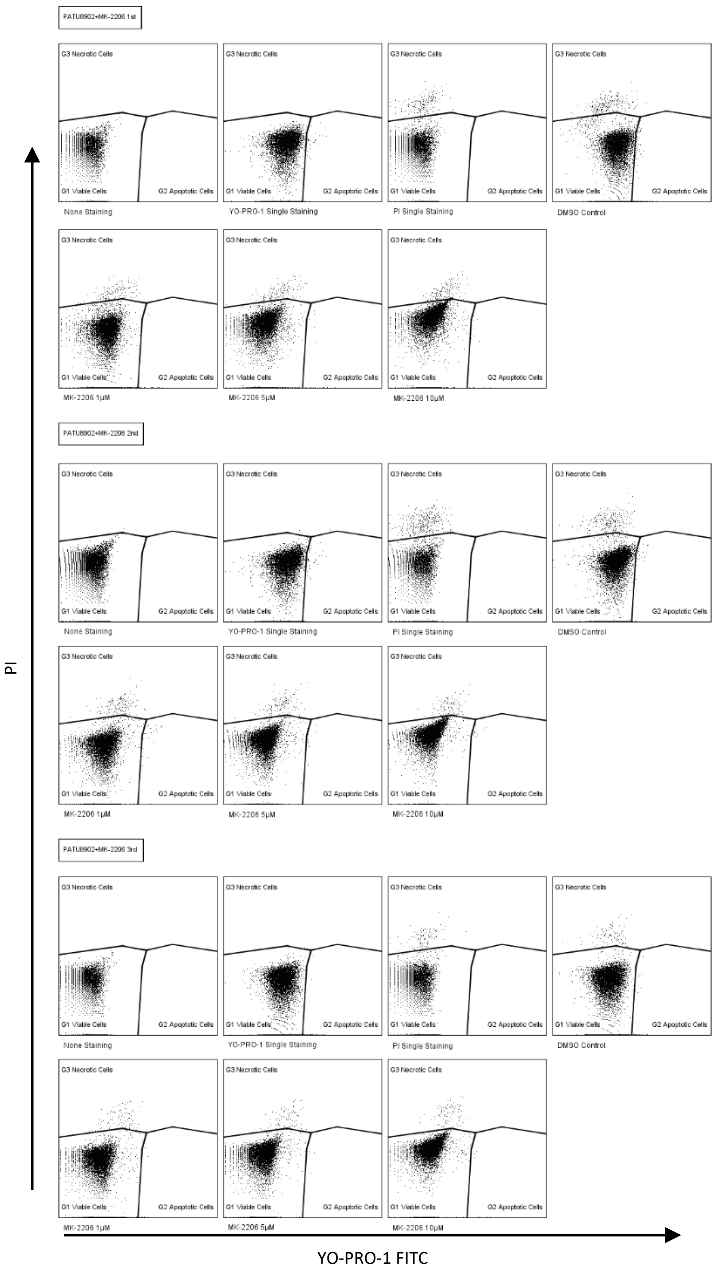

	1st		2nd		3rd		Mean	SD	P-Value (vs. Control)
Concentration	G2(YP+/PI-)	G3(PI+)	G2(YP+/PI-)	G3(PI+)	G2(YP+/PI-)	G3(PI+)	G2+G3		G2+G3
Control	2.42	2.41	0.48	2.77	0.37	0.90	3.12	1.46	
1μM	0.22	1.31	0.29	1.52	0.21	0.91	1.49	0.28	0.15
5μM	0.18	1.49	0.12	1.33	0.12	0.88	1.37	0.28	0.12
10μM	0.18	1.54	0.11	1.11	0.087	0.92	1.32	0.30	0.11

Supplementary Figure S7 – Apoptosis/Necrosis Dot Plot – MK-2206 (5)

PATU8988S

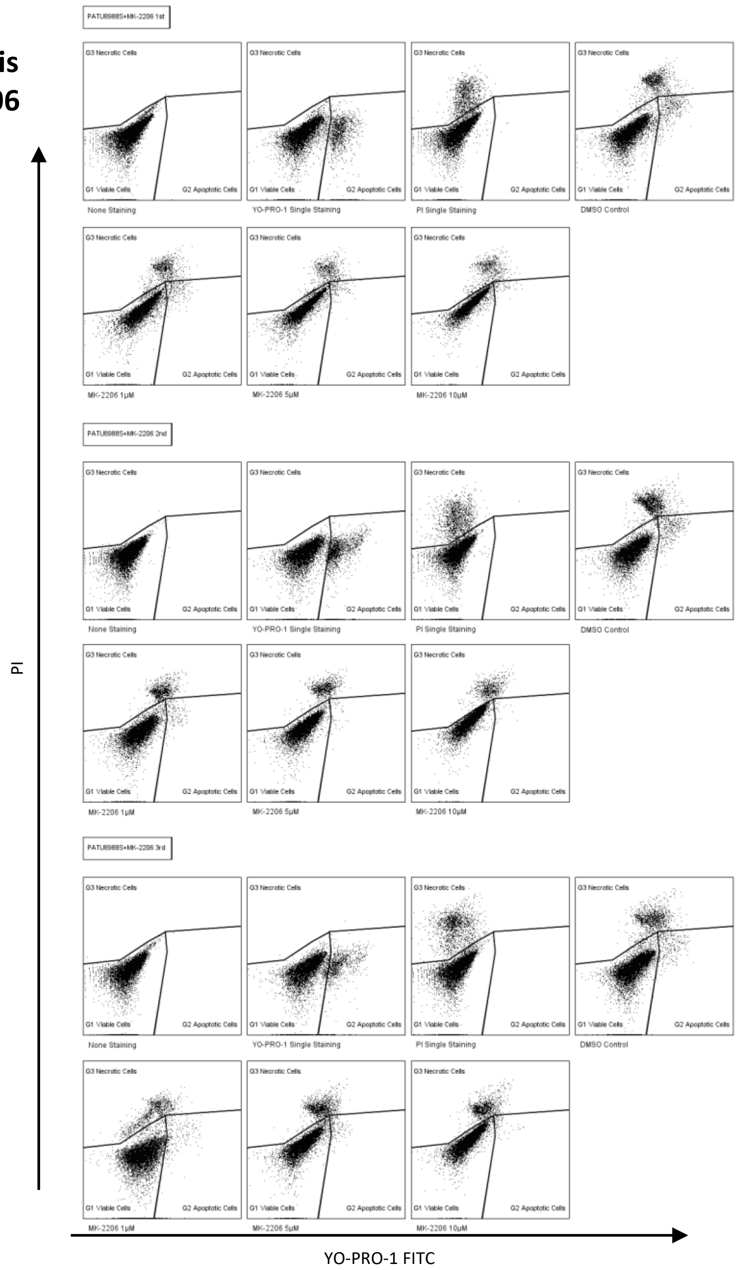

	1st		2nd		3rd		Mean	SD	P-Value (vs. Control)
Concentration	G2(YP⁺/PI⁻)	G3(PI⁺)	G2(YP⁺/PI⁻)	G3(PI⁺)	G2(YP⁺/PI⁻)	G3(PI⁺)	G2+G3		G2+G3
Control	5.01	12.7	4.82	17.7	3.4	13.9	19.18	2.37	
1μM	3.41	9.78	1.90	10.5	2.73	10.7	13.01	0.44	0.42
5μM	1.86	6.84	0.52	10.1	1.63	13.7	11.55	2.79	0.09
10μM	1.13	8.14	0.46	8.75	0.97	11.5	10.32	1.52	0.04

PATU8988T

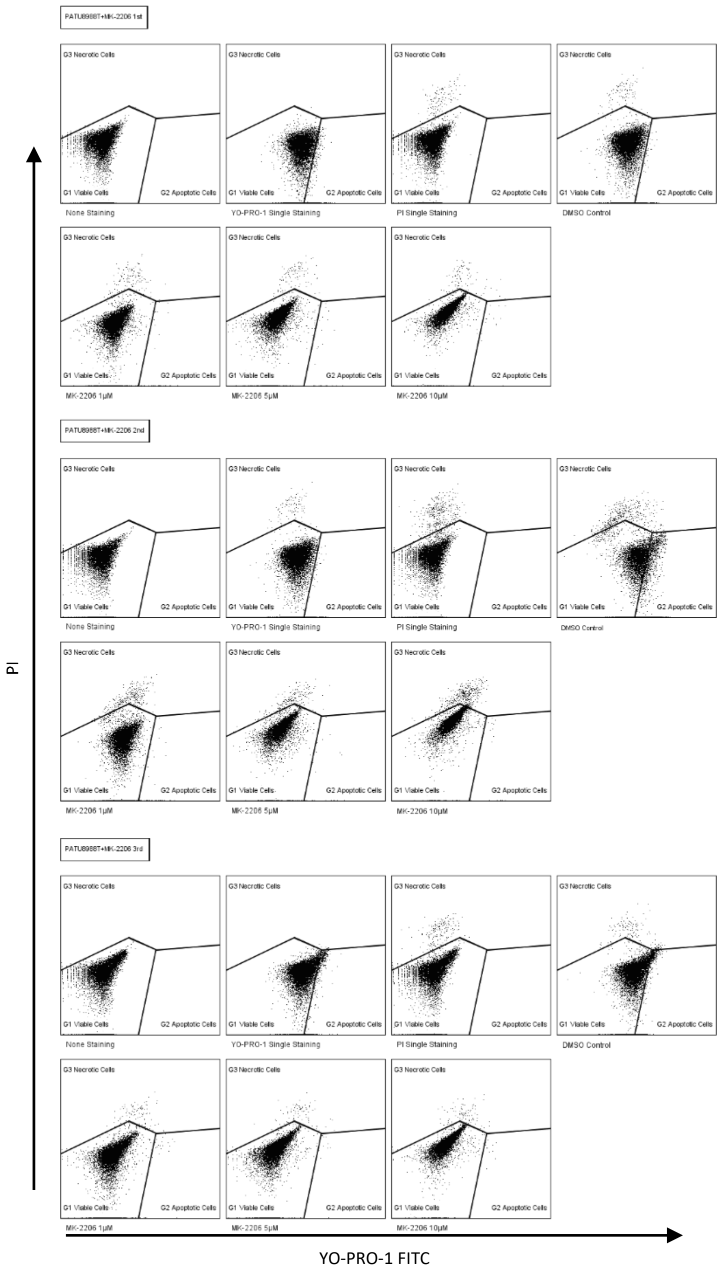

	1st		2nd		3rd		Mean	SD	P-Value (vs. Control)
Concentration	G2(YP⁺/PI⁻)	G3(PI⁺)	G2(YP⁺/PI⁻)	G3(PI⁺)	G2(YP⁺/PI⁻)	G3(PI⁺)	G2+G3		G2+G3
Control	0.10	0.94	0.260	3.92	0.010	1.19	2.14	1.45	
1μM	0.11	1.24	0.086	3.03	0.005	0.96	1.81	0.94	>0.99
5μM	0.07	1.14	0.029	2.06	0.005	0.78	1.36	0.54	>0.99
10μM	0.02	0.98	0.048	4.73	0.000	0.94	2.24	1.80	>0.99

Supplementary Figure S7 – Apoptosis/Necrosis Dot Plot – MK-2206 (6)

SU.86.86

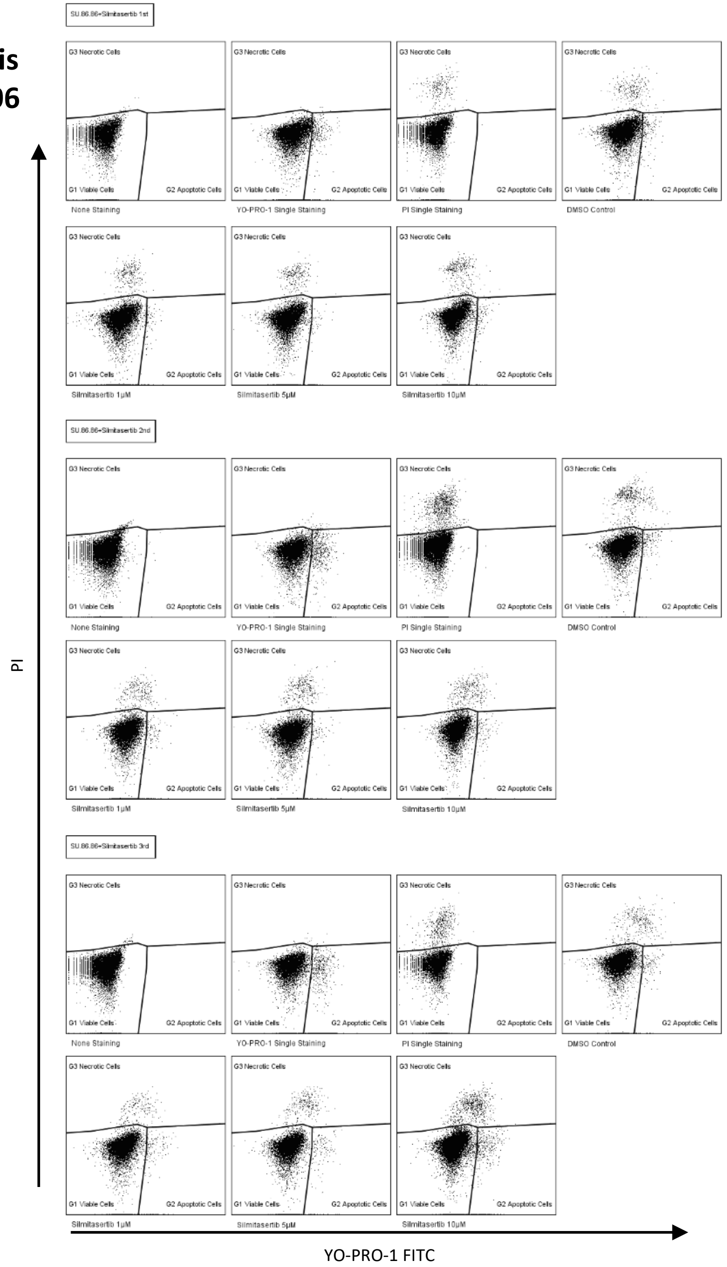

	1st		2nd		3rd		Mean	SD	P-Value (vs. Control)
Concentration	G2(YP$^+$/PI$^-$)	G3(PI$^+$)	G2(YP$^+$/PI$^-$)	G3(PI$^+$)	G2(YP$^+$/PI$^-$)	G3(PI$^+$)	G2+G3		G2+G3
Control	1.40	2.73	1.98	4.49	1.47	4.11	3.78	0.76	
1μM	0.41	3.3	1.42	4.19	3.22	10.80	6.10	3.35	0.68
5μM	0.31	4.14	1.87	6.29	1.99	6.37	5.60	1.03	0.87
10μM	0.68	5.24	1.71	7.18	1.29	4.92	5.78	1.00	0.86

T3M4

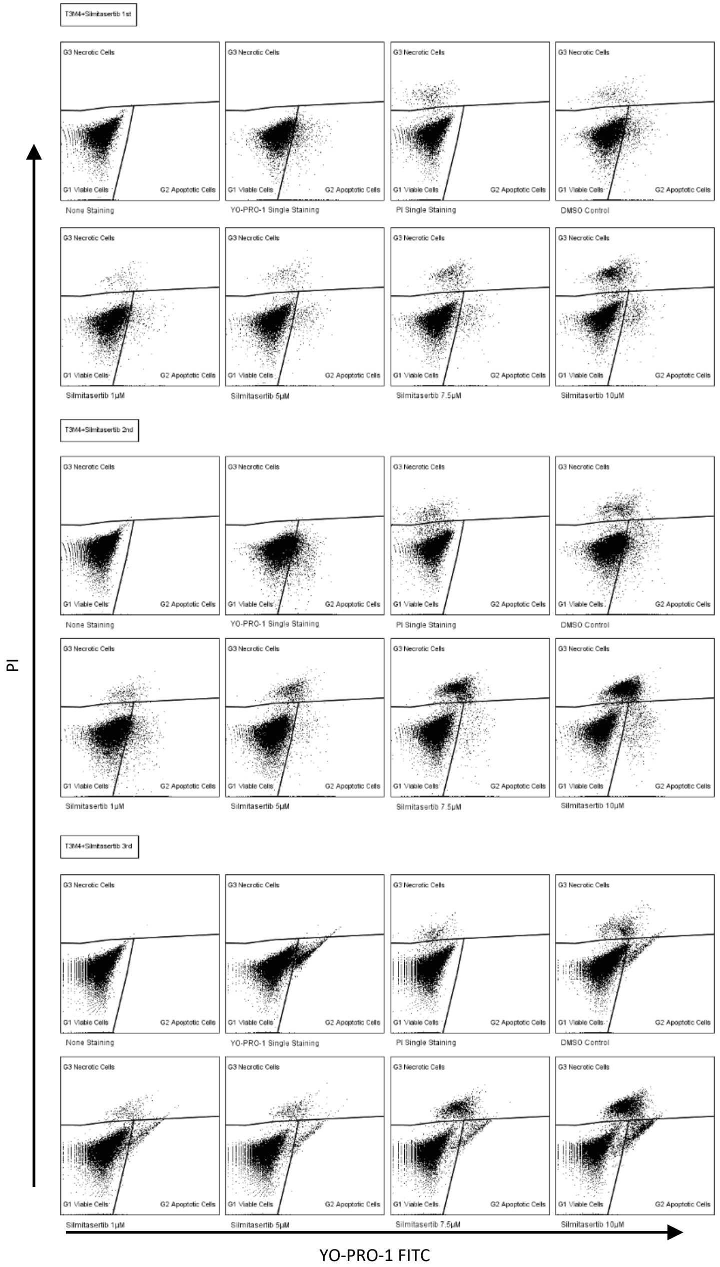

	1st		2nd		3rd		Mean	SD	P-Value (vs. Control)
Concentration	G2(YP$^+$/PI$^-$)	G3(PI$^+$)	G2(YP$^+$/PI$^-$)	G3(PI$^+$)	G2(YP$^+$/PI$^-$)	G3(PI$^+$)	G2+G3		G2+G3
Control	8.02	2.66	6.00	1.85	8.58	3.96	10.36	1.93	
1μM	7.21	3.62	6.80	2.03	7.55	5.05	10.75	1.54	0.99
5μM	4.30	3.89	4.26	2.80	3.21	3.28	7.25	0.71	0.18
10μM	4.04	7.97	2.66	7.57	2.91	5.01	10.05	1.67	>0.99

Figure S7. Apoptosis/necrosis dot plot in ten PDAC cell lines after 72h MK-2206 exposure. The cell population strategy is based on the manufacturer's recommendation with minor improvements. G1: Viable cells; G2: Apoptotic cells; G3: Necrotic cells.
Source: https://www.thermofisher.com/ (accessed on 14. Oct. 2021)

Supplementary Figure S8 – Apoptosis/Necrosis Dot Plot – Buparlisib (1)

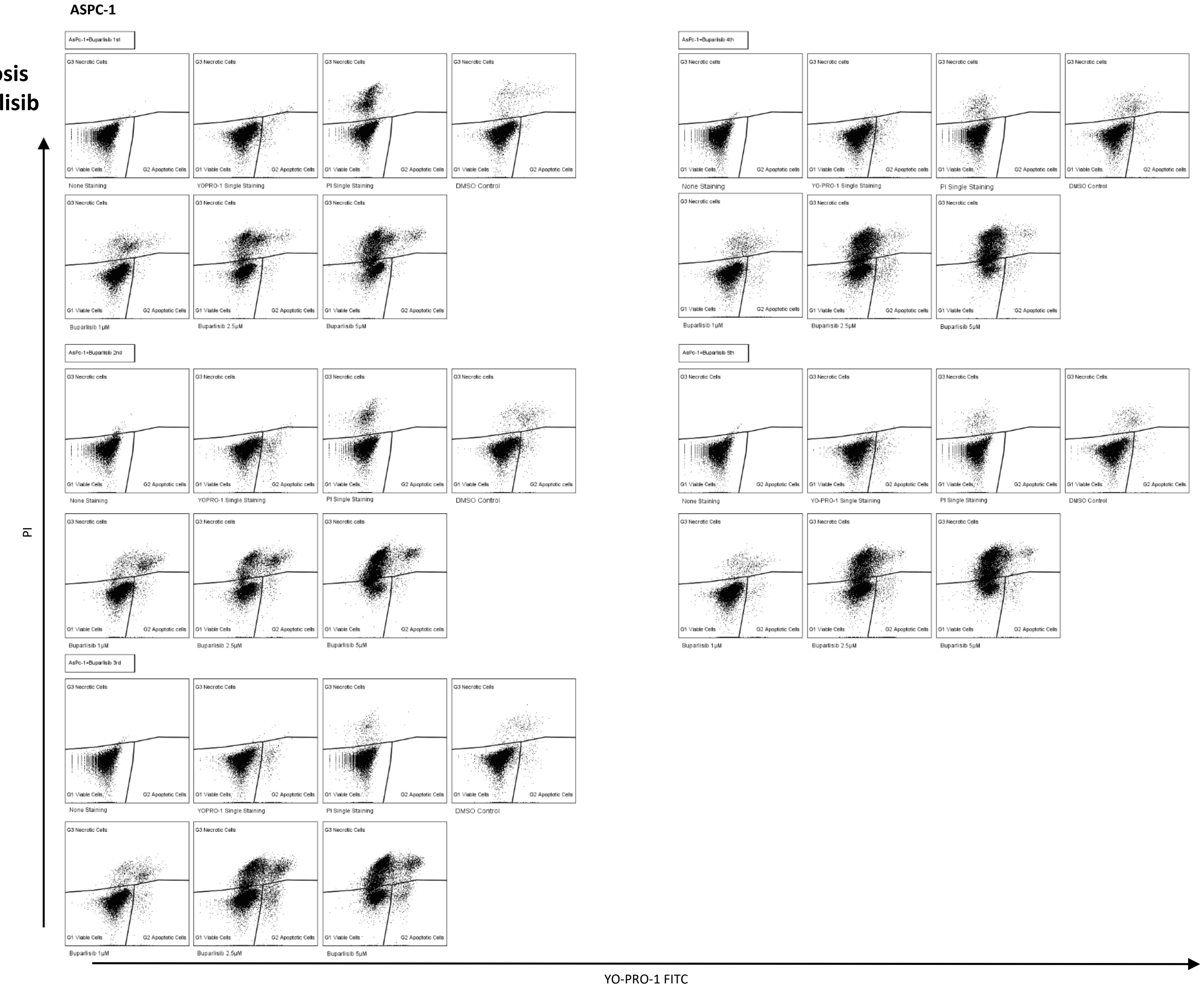

	1st		2nd		3rd		4th		5th		Mean	SD	P-Value (vs. Control)
Concentration	G2(YP⁺/PI⁻)	G3(PI⁺)	G2(YP⁺/PI⁻)	G3(PI⁺)	G2(YP⁺/PI⁻)	G3(PI⁺)	G2(YP⁺/PI⁻)	G3(PI⁺)	G2(YP⁺/PI⁻)	G3(PI⁺)	G2+G3		
Control	0.51	4.53	3.26	5.64	1.60	3.05	1.63	5.47	1.08	3.63	6.08	1.67	
1µM	0.66	11.5	3.13	14.9	2.66	9.9	2.72	8.60	2.23	6.35	12.53	3.08	0.15
2.5µM	1.13	37.8	2.39	36.7	6.20	32.3	2.77	46.4	2.94	39.8	41.69	4.04	<0.001
5µM	0.42	48.8	0.86	67.0	4.53	54.8	1.87	66.8	1.79	64.7	62.31	7.33	<0.001

Supplementary Figure S8 – Apoptosis/Necrosis Dot Plot – Buparlisib (2)

BXPC-3

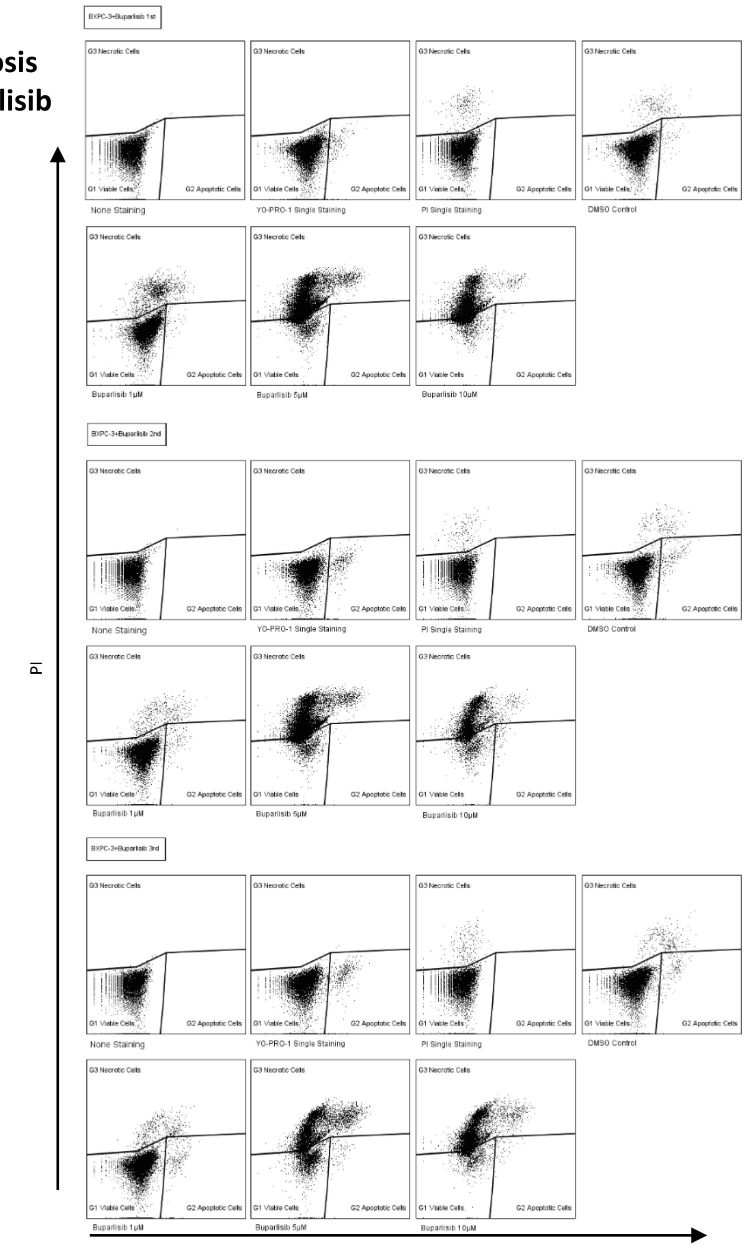

	1st		2nd		3rd		Mean	SD	P-Value (vs. Control)
Concentration	G2(YP⁺/PI⁻)	G3(PI⁺)	G2(YP⁺/PI⁻)	G3(PI⁺)	G2(YP⁺/PI⁻)	G3(PI⁺)	G2+G3		G2+G3
Control	0.55	2.23	1.86	1.92	1.68	3.07	3.77	0.80	
1μM	0.56	14.2	1.35	4.63	2.52	5.68	9.65	3.73	0.52
5μM	0.04	88.6	1.58	65.9	1.09	75.2	77.47	8.68	<0.001
10μM	0.19	85.7	1.08	81.3	0.73	87.7	85.57	2.48	<0.001

CAPAN-1

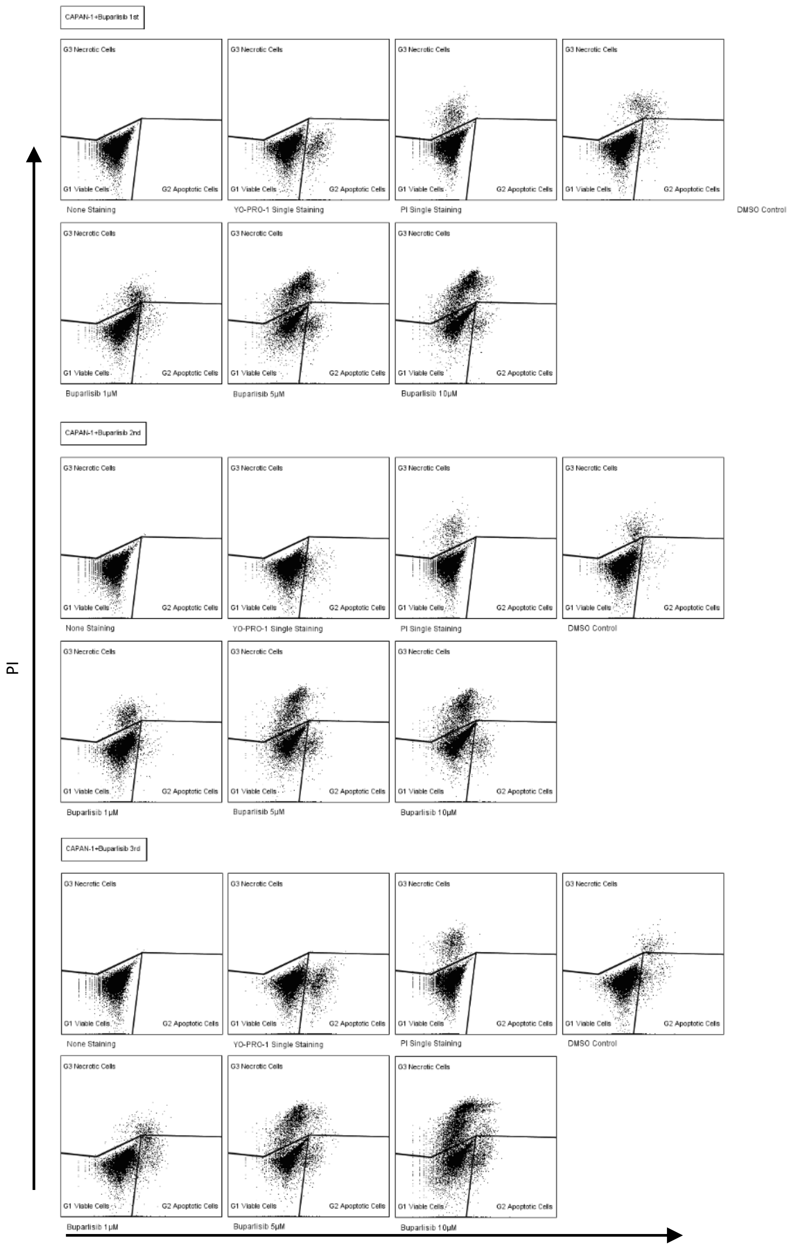

	1st		2nd		3rd		Mean	SD	P-Value (vs. Control)
Concentration	G2(YP⁺/PI⁻)	G3(PI⁺)	G2(YP⁺/PI⁻)	G3(PI⁺)	G2(YP⁺/PI⁻)	G3(PI⁺)	G2+G3		G2+G3
Control	1.67	6.88	1.55	4.76	5.01	9.13	9.67	3.29	
1μM	2.13	8.63	1.99	9.67	5.79	7.12	11.78	0.88	0.74
5μM	6.62	32.0	5.18	25.6	4.46	29.9	34.59	3.20	0.004
10μM	3.49	34.6	4.50	27.5	4.79	37.3	37.39	4.15	0.005

Supplementary Figure S8 – Apoptosis/Necrosis Dot Plot – Buparlisib (3)

COLO357

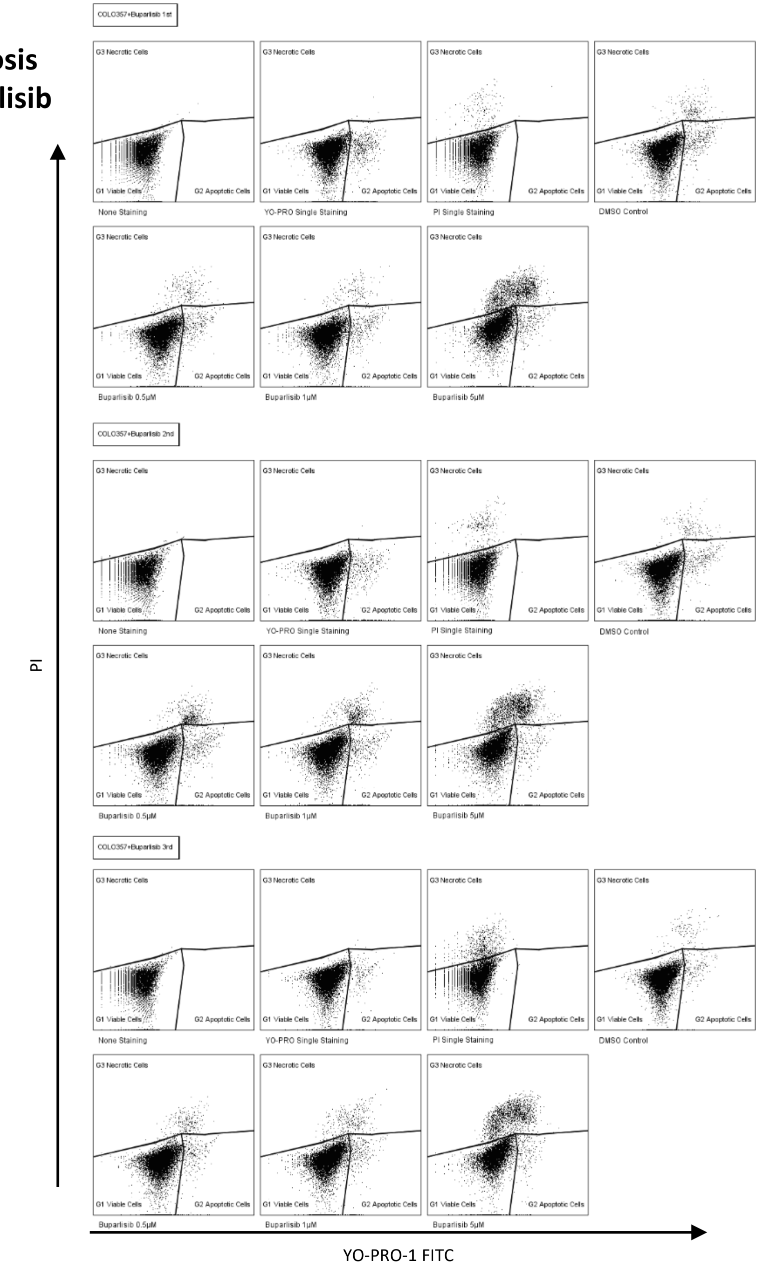

	1st		2nd		3rd		Mean	SD	P-Value (vs. Control)
Concentration	G2(YP⁺/PI⁻)	G3(PI⁺)	G2(YP⁺/PI⁻)	G3(PI⁺)	G2(YP⁺/PI⁻)	G3(PI⁺)	G2+G3		G2+G3
Control	4.00	2.20	3.31	1.64	1.59	1.04	4.59	1.48	
0.5µM	3.53	2.38	4.92	4.53	2.89	2.72	6.99	1.74	0.24
1µM	3.89	1.74	3.71	5.49	2.86	3.44	7.04	1.55	0.24
5µM	3.24	19.6	2.51	21.0	1.97	19.4	22.57	0.89	<0.001

PANC-1

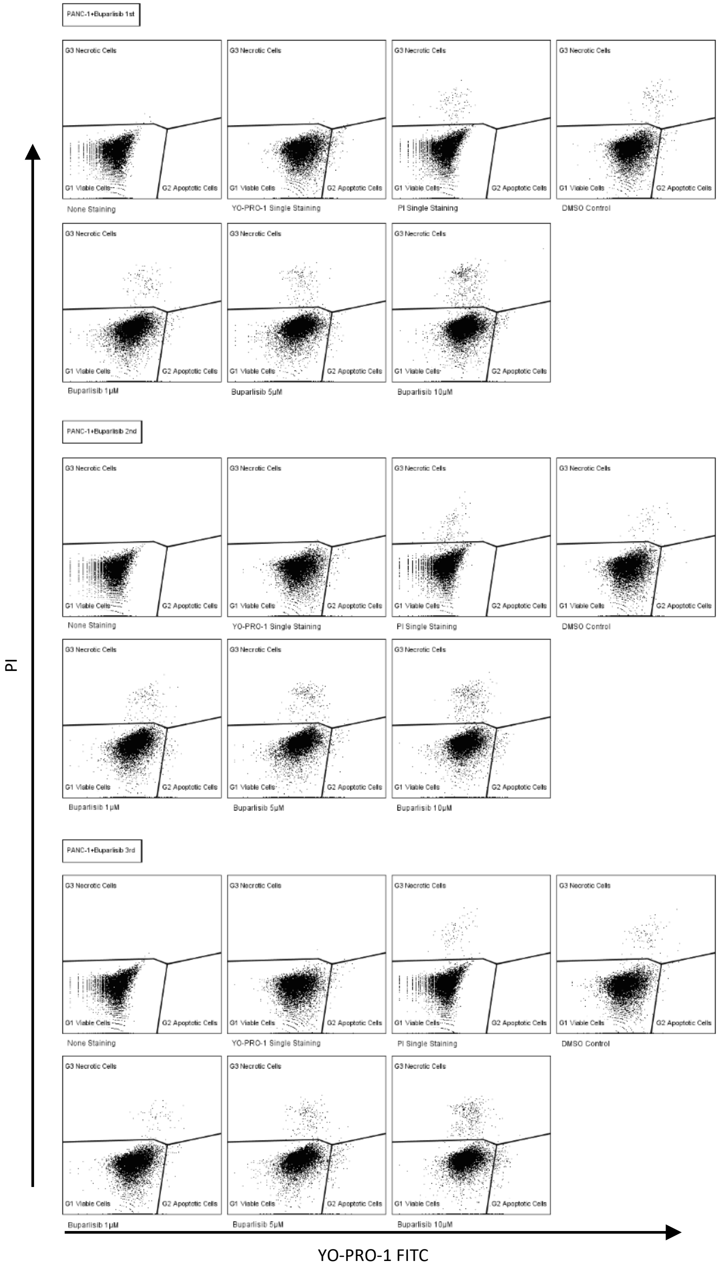

	1st		2nd		3rd		Mean	SD	P-Value (vs. Control)
Concentration	G2(YP⁺/PI⁻)	G3(PI⁺)	G2(YP⁺/PI⁻)	G3(PI⁺)	G2(YP⁺/PI⁻)	G3(PI⁺)	G2+G3		G2+G3
Control	0.60	1.15	0.56	0.58	0.18	1.08	1.38	0.26	
1µM	0.46	1.25	1.16	1.60	0.33	0.66	1.82	0.73	0.81
5µM	0.55	1.66	1.25	2.54	1.50	2.11	3.20	0.71	0.04
10µM	0.78	3.47	1.52	3.76	1.86	3.77	5.05	0.59	<0.001

Supplementary Figure S8 – Apoptosis/Necrosis Dot Plot – Buparlisib (4)

PATU8902

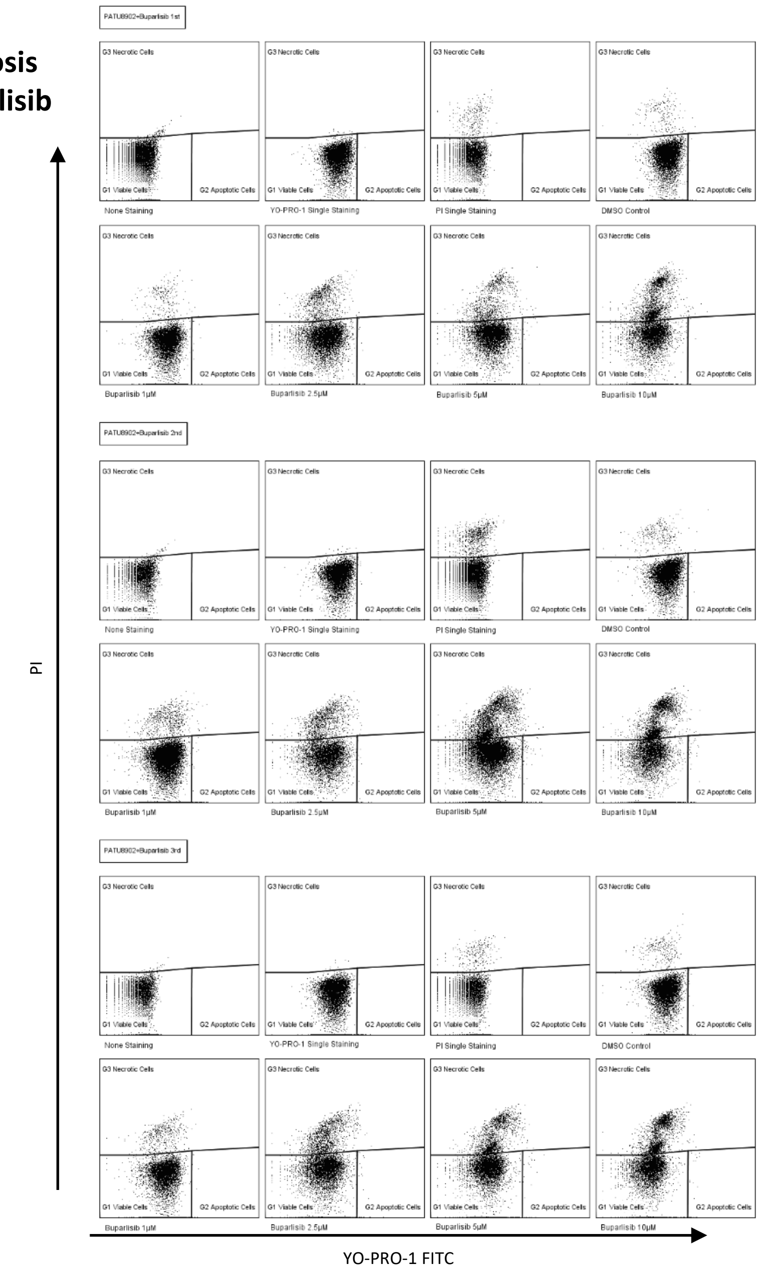

	1st		2nd		3rd		Mean	SD	P-Value (vs. Control)
Concentration	G2(YP+/PI-)	G3(PI+)	G2(YP+/PI-)	G3(PI+)	G2(YP+/PI-)	G3(PI+)	G2+G3		G2+G3
Control	0.32	1.96	0.49	2.81	0.24	2.21	2.68	0.45	
1μM	0.13	2.20	0.31	3.61	0.14	4.36	3.58	0.92	0.99
2.5μM	0.12	8.53	0.28	12.5	0.32	14.2	11.98	2.46	0.01
5μM	0.20	9.97	0.37	17.4	0.23	19.4	15.86	4.09	0.001
10μM	0.11	25.1	0.33	30.1	0.19	23.5	26.44	2.89	<0.001

PATU8988S

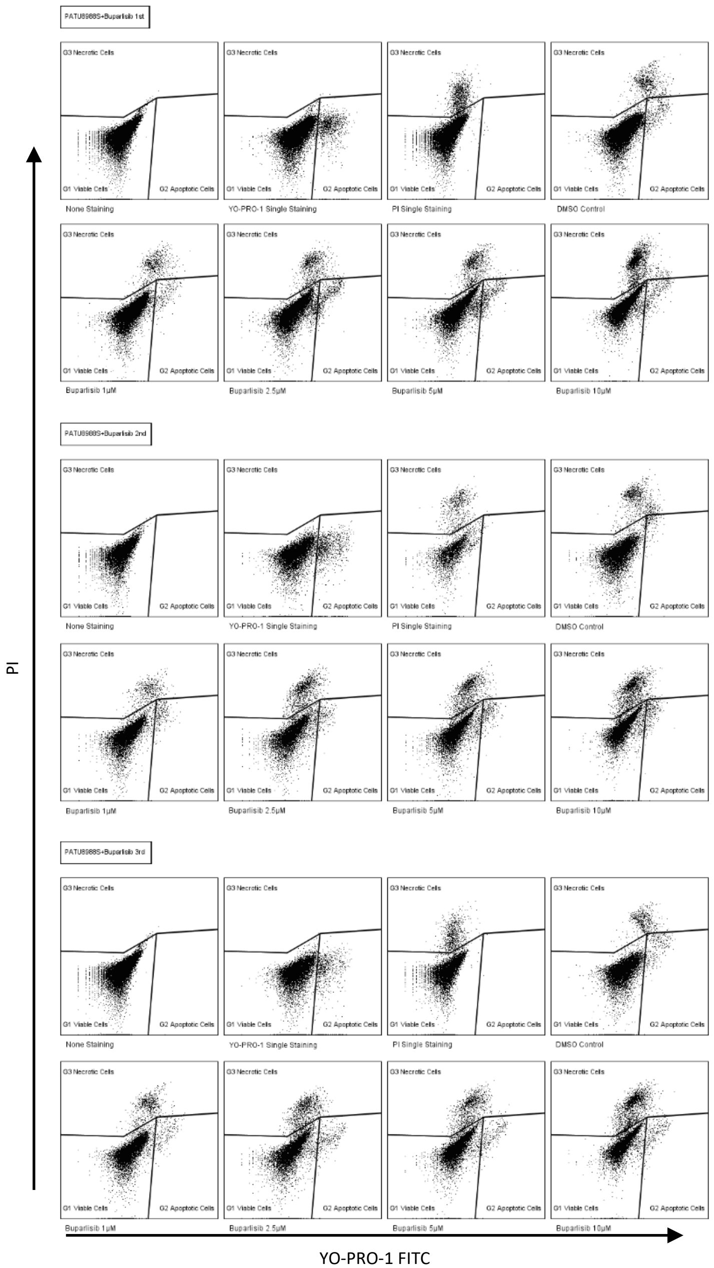

	1st		2nd		3rd		Mean	SD	P-Value (vs. Control)
Concentration	G2(YP+/PI-)	G3(PI+)	G2(YP+/PI-)	G3(PI+)	G2(YP+/PI-)	G3(PI+)	G2+G3		G2+G3
Control	4.93	8.70	2.45	9.28	3.64	5.27	11.42	1.94	
1μM	3.56	9.06	2.91	5.31	3.32	12.4	12.19	3.08	>0.99
2.5μM	5.30	11.3	2.11	13.9	2.82	8.19	14.54	2.51	0.55
5μM	3.70	12.2	2.93	14.2	2.96	13.4	16.46	0.51	0.19
10μM	3.57	27.5	3.42	19.8	3.09	23.3	26.89	3.22	<0.001

Supplementary Figure S8 – Apoptosis/Necrosis Dot Plot – Buparlisib (5)

PATU8988T

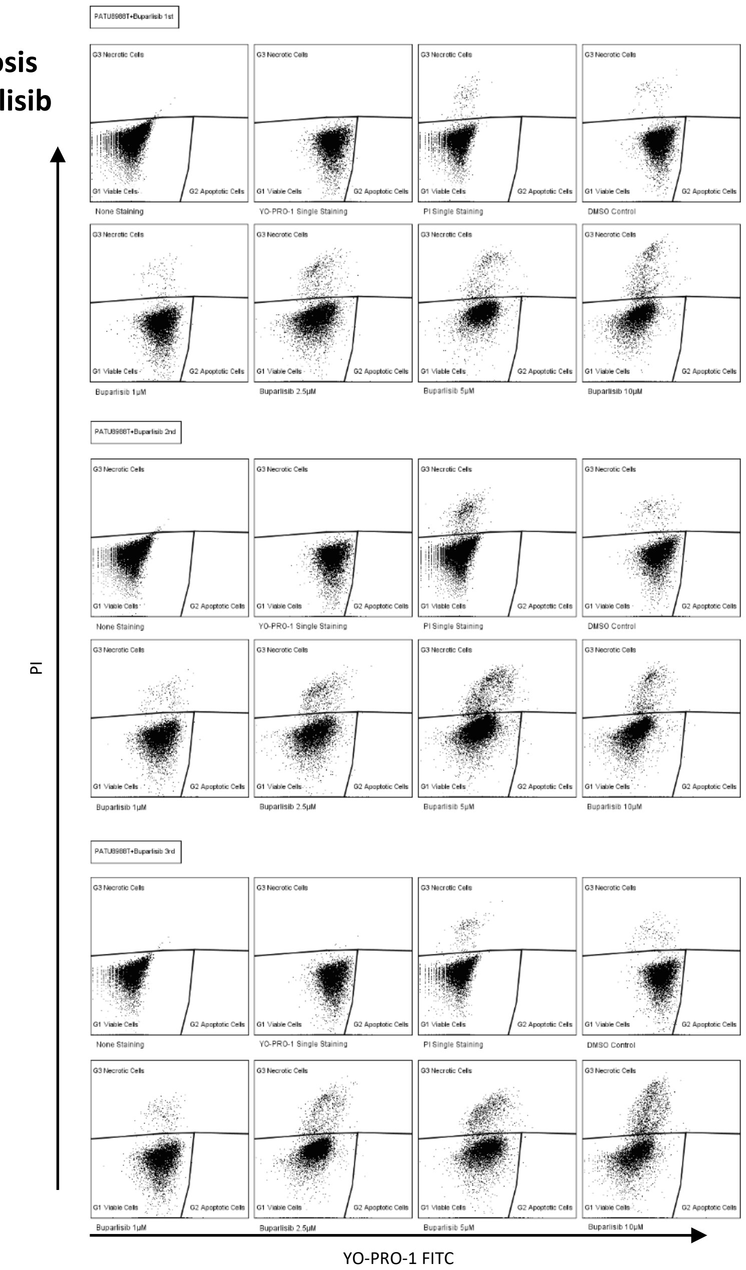

	1st		2nd		3rd		Mean	SD	P-Value (vs. Control)
Concentration	G2(YP⁺/PI⁻)	G3(PI⁺)	G2(YP⁺/PI⁻)	G3(PI⁺)	G2(YP⁺/PI⁻)	G3(PI⁺)	G2+G3		G2+G3
Control	0.63	0.99	0.62	2.39	1.79	1.79	2.74	0.82	
1μM	0.17	1.13	0.34	2.00	0.37	2.62	2.21	0.70	>0.99
2.5μM	0.15	6.53	0.24	8.25	0.20	8.89	8.09	1.02	0.13
5μM	0.12	6.35	0.26	10.7	0.48	13.1	10.34	2.94	0.03
10μM	0.053	11.2	0.12	9.48	0.025	18.7	13.19	3.97	0.004

SU.86.86

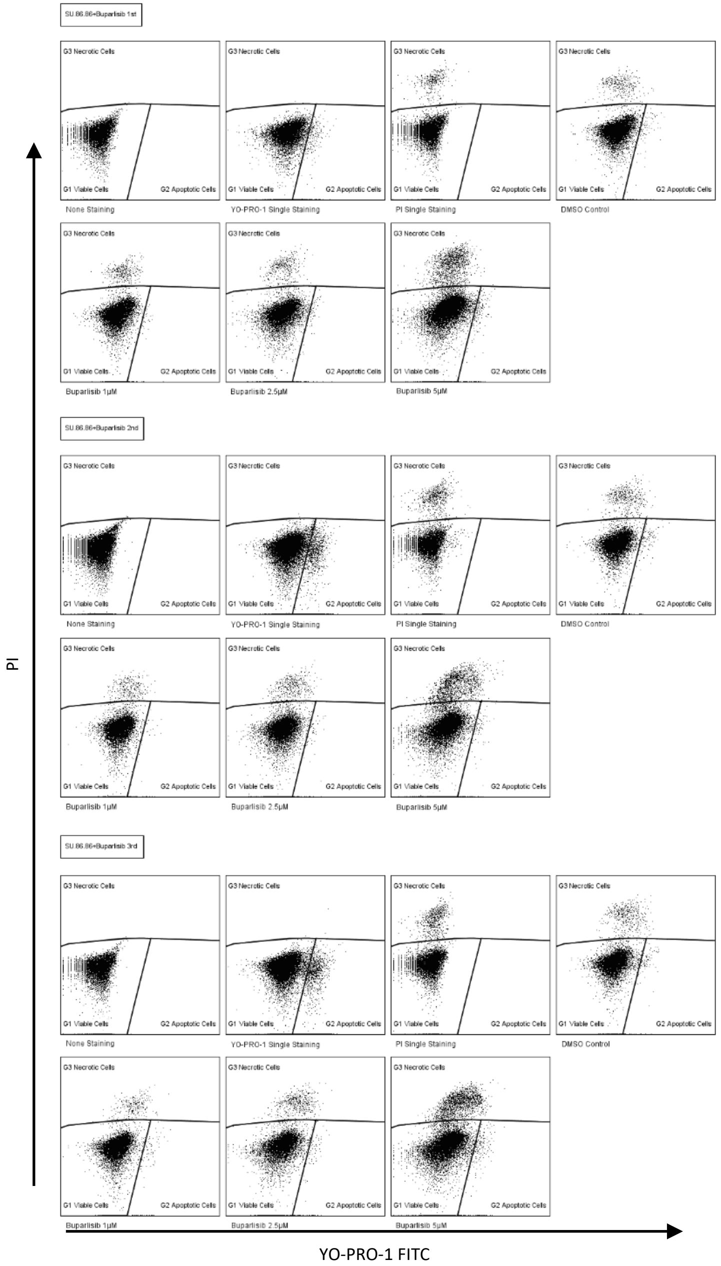

	1st		2nd		3rd		Mean	SD	P-Value (vs. Control)
Concentration	G2(YP⁺/PI⁻)	G3(PI⁺)	G2(YP⁺/PI⁻)	G3(PI⁺)	G2(YP⁺/PI⁻)	G3(PI⁺)	G2+G3		G2+G3
Control	1.75	4.01	1.8	5.89	1.62	5.69	6.92	0.83	
1μM	0.70	4.26	0.77	3.28	1.15	3.08	4.41	0.39	0.15
2.5μM	1.27	4.76	0.97	4.96	1.31	6.13	6.47	0.69	0.96
5μM	1.43	9.14	0.86	12.1	1.91	13.7	13.05	2.06	0.002

Supplementary Figure S8 – Apoptosis/Necrosis Dot Plot – Buparlisib (6)

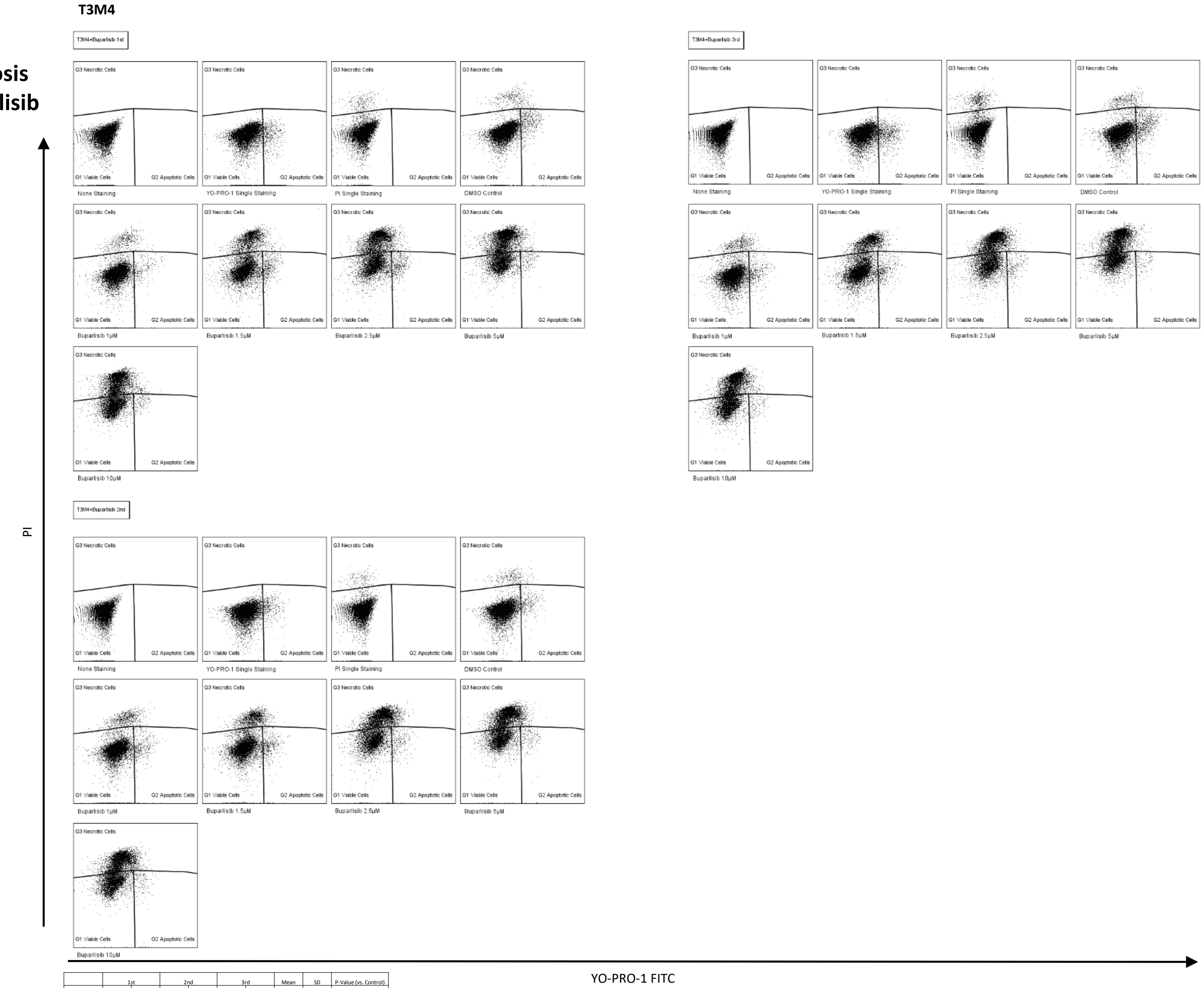

	1st		2nd		3rd		Mean	SD	P-Value (vs. Control)
Concentration	G2(YP⁺/PI⁻)	G3(PI⁺)	G2(YP⁺/PI⁻)	G3(PI⁺)	G2(YP⁺/PI⁻)	G3(PI⁺)	G2+G3		G2+G3
Control	5.23	3.56	3.04	2.63	7.66	3.78	8.63	2.36	
1μM	2.86	5.12	4.15	6.63	3.74	5.37	9.29	1.15	>0.99
1.5μM	4.63	21.9	4.27	15.5	4.29	22.6	24.40	3.27	<0.001
2.5μM	4.74	41.3	3.51	43.3	3.04	46.6	47.50	1.55	<0.001
5μM	2.13	55.1	2.32	50.4	1.56	54.0	55.17	1.86	<0.001
10μM	2.30	47.6	2.32	51.0	1.92	49.4	51.51	1.40	<0.001

Figure S8. Apoptosis/necrosis dot plot in ten PDAC cell lines after 72h Buparlisib exposure. The cell population strategy is based on the manufacturer's recommendation with minor improvements. G1: Viable cells; G2: Apoptotic cells; G3: Necrotic cells.
Source: https://www.thermofisher.com/ (accessed on 14. Oct. 2021)
