# Supplementary material for: The Inhibitory Response to PI3K/AKT Pathway Inhibitors MK-2206 and Buparlisib Is Related to Genetic Differences in Pancreatic Ductal Adenocarcinoma Cell Lines
Source: Int J Mol Sci. 2022 Apr 13;23(8):4295. doi: 10.3390/ijms23084295 (PMC9029322; doi:10.3390/ijms23084295)
Supplement: Supplementary file 1 [file ijms-23-04295-s001.zip › Supplementary Tables.pdf]

Table S1 - Cell Viability MK-2206 (to 100%Control)

AsPc-1

Concentration	1st	2nd	3rd	Mean	SD	P-value (vs. Control)
	Proliferation					
Concentration	1st	2nd	3rd	Mean	SD	P-value (vs. Control)
Control	100.00%	100.00%	100.00%	100.00%	0.00%	
1μM	62.86%	93.64%	72.03%	76.18%	15.80%	0.05
5μM	45.71%	68.21%	59.32%	57.75%	11.33%	0.002
10μM	21.43%	10.40%	15.68%	15.84%	5.52%	<0.001
	Metabolic activity					
Concentration	1st	2nd	3rd	Mean	SD	P-value (vs. Control)
Control	100.00%	100.00%	100.00%	100.00%	0.00%	
1μM	86.91%	94.75%	92.02%	91.23%	3.98%	0.04
5μM	89.76%	89.36%	80.16%	86.43%	5.43%	0.004
10μM	31.22%	35.06%	34.94%	33.74%	2.18%	<0.001
	Biomass					
Concentration	1st	2nd	3rd	Mean	SD	P-value (vs. Control)
Control	100.00%	100.00%	100.00%	100.00%	0.00%	
1μM	66.25%	87.05%	83.65%	78.98%	11.16%	0.08
5μM	43.80%	71.54%	62.03%	59.12%	14.10%	0.003
10μM	18.32%	4.33%	22.79%	15.15%	9.63%	<0.001

BxPc-3

Concentration	1st	2nd	3rd	Mean	SD	P-value (vs. Control)
	Proliferation					
Concentration	1st	2nd	3rd	Mean	SD	P-value (vs. Control)
Control	100.00%	100.00%	100.00%	100.00%	0.00%	
1μM	67.20%	62.85%	73.13%	67.73%	5.16%	0.005
5μM	33.12%	62.01%	60.20%	51.78%	16.18%	<0.001
10μM	13.83%	8.38%	17.01%	13.07%	4.36%	<0.001
	Metabolic activity					
Concentration	1st	2nd	3rd	Mean	SD	P-value (vs. Control)
Control	100.00%	100.00%	100.00%	100.00%	0.00%	
1μM	99.66%	83.07%	94.81%	92.51%	8.53%	0.64
5μM	71.68%	79.54%	87.73%	79.65%	8.03%	0.06
10μM	20.50%	10.80%	38.40%	23.23%	14.00%	<0.001
	Biomass					
Concentration	1st	2nd	3rd	Mean	SD	P-value (vs. Control)
Control	100.00%	100.00%	100.00%	100.00%	0.00%	
1μM	72.75%	62.56%	68.64%	67.98%	5.13%	<0.001
5μM	35.45%	38.83%	37.60%	37.29%	1.71%	<0.001
10μM	20.02%	9.71%	15.15%	14.96%	5.16%	<0.001

Capan-1

Concentration	1st	2nd	3rd	Mean	SD	P-value (vs. Control)
	Proliferation					
Concentration	1st	2nd	3rd	Mean	SD	P-value (vs. Control)
Control	100.00%	100.00%	100.00%	100.00%	0.00%	
1μM	94.12%	66.00%	96.88%	85.67%	17.09%	0.21
5μM	41.18%	36.00%	49.17%	42.12%	6.63%	<0.001
10μM	15.69%	10.00%	15.00%	13.56%	3.11%	<0.001
	Metabolic activity					
Concentration	1st	2nd	3rd	Mean	SD	P-value (vs. Control)
Control	100.00%	100.00%	100.00%	100.00%	0.00%	
1μM	93.70%	93.92%	86.78%	91.47%	4.06%	0.18
5μM	60.76%	61.32%	68.51%	63.53%	4.32%	<0.001
10μM	41.22%	48.06%	45.94%	45.07%	3.50%	<0.001
	Biomass					
Concentration	1st	2nd	3rd	Mean	SD	P-value (vs. Control)
Control	100.00%	100.00%	100.00%	100.00%	0.00%	
1μM	97.86%	80.42%	96.95%	91.74%	9.82%	0.24
5μM	68.71%	73.64%	78.81%	73.72%	5.05%	0.001
10μM	11.64%	15.12%	14.14%	13.63%	1.79%	<0.001

Colo357

Concentration	1st	2nd	3rd	4th	5th	Mean	SD	P-value (vs. Control)
	Proliferation							
Concentration	1st	2nd	3rd	4th	5th	Mean	SD	P-value (vs. Control)
Control	100.00%	100.00%	100.00%	100.00%	100.00%	100.00%	0.00%	
1μM	74.43%	85.39%	100.44%	81.05%	80.70%	84.40%	9.78%	0.02
5μM	51.53%	46.07%	61.84%	77.02%	62.81%	59.85%	11.90%	<0.001
10μM	49.62%	47.19%	41.67%	42.34%	34.39%	43.04%	5.87%	<0.001
	Metabolic activity							
Concentration	1st	2nd	3rd	4th	5th	Mean	SD	P-value (vs. Control)
Control	100.00%	100.00%	100.00%	100.00%	100.00%	100.00%	0.00%	
1μM	124.04%	101.23%	108.63%	102.41%	82.94%	103.85%	14.80%	0.92
5μM	52.80%	69.86%	79.38%	63.75%	68.21%	66.80%	9.68%	0.005
10μM	54.86%	33.18%	60.54%	66.95%	46.55%	52.42%	13.11%	<0.001
	Biomass							
Concentration	1st	2nd	3rd	4th		Mean	SD	P-value (vs. Control)
Control	100.00%	100.00%	100.00%	100.00%		100.00%	0.00%	
1μM	87.05%	94.81%	89.31%	60.01%		82.80%	15.54%	0.17
5μM	71.54%	60.24%	79.77%	37.69%		62.31%	18.26%	0.002
10μM	18.32%	45.17%	37.07%	23.64%		31.05%	12.28%	<0.001

Panc-1

Concentration	1st	2nd	3rd	Mean	SD	P-value (vs. Control)
	Proliferation					
Concentration	1st	2nd	3rd	Mean	SD	P-value (vs. Control)
Control	100.00%	100.00%	100.00%	100.00%	0.00%	
1μM	71.65%	62.96%	59.67%	64.76%	6.19%	<0.001
5μM	65.35%	53.70%	53.33%	57.46%	6.84%	<0.001
10μM	26.77%	27.78%	20.67%	25.07%	3.85%	<0.001
	Metabolic activity					
Concentration	1st	2nd	3rd	Mean	SD	P-value (vs. Control)
Control	100.00%	100.00%	100.00%	100.00%	0.00%	
1μM	80.15%	73.64%	88.84%	80.88%	7.63%	0.01
5μM	71.62%	65.79%	83.97%	73.79%	9.28%	0.002
10μM	48.76%	49.25%	43.66%	47.22%	3.10%	<0.001
	Biomass					
Concentration	1st	2nd	3rd	Mean	SD	P-value (vs. Control)
Control	100.00%	100.00%	100.00%	100.00%	0.00%	
1μM	63.16%	66.58%	61.29%	63.68%	2.68%	<0.001
5μM	56.93%	55.81%	56.24%	56.33%	0.57%	<0.001
10μM	24.52%	37.13%	29.22%	30.29%	6.37%	<0.001

PaTu8902

Concentration	1st	2nd	3rd	4th	5th	Mean	SD	P-value (vs. Control)
	Proliferation							
Concentration	1st	2nd	3rd	4th		Mean	SD	P-value (vs. Control)
Control	100.00%	100.00%	100.00%	100.00%		100.00%	0.00%	
1μM	69.66%	78.00%	78.85%	71.00%		74.38%	4.72%	0.004
5μM	59.10%	62.00%	39.42%	56.61%		54.28%	10.15%	<0.001
10μM	24.27%	15.00%	10.58%	41.96%		22.95%	13.90%	<0.001
	Metabolic activity							
Concentration	1st	2nd	3rd	4th		Mean	SD	P-value (vs. Control)
Control	100.00%	100.00%	100.00%	100.00%		100.00%	0.00%	
1μM	89.77%	83.76%	81.42%	77.92%		83.22%	4.98%	0.69
5μM	71.12%	71.00%	73.68%	74.62%		72.61%	1.83%	0.05
10μM	21.78%	42.64%	40.15%	29.58%		33.54%	9.67%	<0.001
	Biomass							
Concentration	1st	2nd	3rd	4th	5th	Mean	SD	P-value (vs. Control)
Control	100.00%	100.00%	100.00%	100.00%	100.00%	100.00%	0.00%	
1μM	63.26%	61.97%	54.82%	69.43%	56.63%	61.22%	5.79%	<0.001
5μM	58.76%	50.78%	24.17%	49.86%	53.47%	47.41%	13.44%	<0.001
10μM	23.45%	37.28%	19.93%	47.25%	45.01%	34.58%	12.40%	<0.001

PaTu8988S

	Proliferation							
Concentration	1st	2nd	3rd	4th		Mean	SD	P-value (vs. Control)
Control	100.00%	100.00%	100.00%	100.00%		100.00%	0.00%	
1μM	86.44%	84.30%	81.52%	79.07%		82.83%	3.22%	<0.001
5μM	33.90%	43.60%	51.09%	43.02%		42.90%	7.04%	<0.001
10μM	8.47%	7.75%	17.39%	18.60%		13.05%	5.74%	<0.001
	Metabolic activity							
Concentration	1st	2nd	3rd			Mean	SD	P-value (vs. Control)
Control	100.00%	100.00%	100.00%			100.00%	0.00%	
1μM	92.35%	91.65%	88.54%			90.85%	2.03%	0.62
5μM	63.25%	80.71%	58.50%			67.49%	11.70%	0.005
10μM	49.68%	38.29%	34.60%			40.86%	7.86%	<0.001
	Biomass							
Concentration	1st	2nd	3rd	4th		Mean	SD	P-value (vs. Control)
Control	100.00%	100.00%	100.00%	100.00%		100.00%	0.00%	
1μM	56.92%	55.07%	50.78%	58.30%		55.27%	3.27%	<0.001
5μM	45.04%	51.31%	40.51%	42.52%		44.85%	4.69%	<0.001
10μM	30.52%	43.07%	33.45%	31.89%		34.73%	5.69%	<0.001

PaTu8988T

	Proliferation							
Concentration	1st	2nd	3rd	4th	5th	Mean	SD	P-value (vs. Control)
Control	100.00%	100.00%	100.00%	100.00%	100.00%	100.00%	0.00%	
1μM	70.20%	64.75%	96.81%	66.67%	64.46%	72.58%	13.74%	<0.001
5μM	60.78%	53.24%	43.43%	57.14%	56.20%	54.16%	6.57%	<0.001
10μM	35.29%	28.78%	15.54%	11.90%	12.40%	20.78%	10.62%	<0.001
	Metabolic activity							
Concentration	1st	2nd	3rd	4th		Mean	SD	P-value (vs. Control)
Control	100.00%	100.00%	100.00%	100.00%		100.00%	0.00%	
1μM	85.79%	72.69%	98.65%	82.78%		84.98%	10.70%	0.06
5μM	73.00%	69.56%	89.81%	65.67%		74.51%	10.63%	0.002
10μM	52.96%	55.88%	51.30%	40.80%		50.24%	6.57%	<0.001
	Biomass							
Concentration	1st	2nd	3rd	4th	5th	Mean	SD	P-value (vs. Control)
Control	100.00%	100.00%	100.00%	100.00%	100.00%	100.00%	0.00%	
1μM	59.14%	61.16%	73.84%	59.19%	71.41%	64.95%	7.11%	<0.001
5μM	56.77%	53.55%	61.35%	55.61%	65.04%	58.46%	4.66%	<0.001
10μM	20.63%	39.60%	47.70%	27.52%	47.21%	36.53%	12.06%	<0.001

SU.86.86

	Proliferation							
Concentration	1st	2nd	3rd	4th	5th	Mean	SD	P-value (vs. Control)
Control	100.00%	100.00%	100.00%	100.00%	100.00%	100.00%	0.00%	
1μM	91.57%	80.00%	80.10%	80.00%	71.29%	80.59%	7.21%	<0.001
5μM	71.69%	72.73%	70.19%	66.09%	61.39%	68.42%	4.67%	<0.001
7.5μM			59.13%	52.61%	51.49%	54.41%	4.13%	<0.001
10μM	20.48%	5.00%	14.46%	15.22%	9.90%	13.01%	5.84%	<0.001
	Metabolic activity							
Concentration	1st	2nd	3rd	4th	5th	Mean	SD	P-value (vs. Control)
Control	100.00%	100.00%	100.00%	100.00%	100.00%	100.00%	0.00%	
1μM	102.01%	98.05%	81.92%	100.61%	81.87%	92.89%	10.14%	0.93
5μM	84.95%	101.47%	58.47%	110.86%	85.97%	88.34%	19.93%	0.73
7.5μM			54.54%	94.65%	46.62%	65.27%	25.75%	0.06
10μM	50.15%	10.91%	18.13%	77.48%	40.74%	39.48%	26.61%	<0.001
	Biomass							
Concentration	1st	2nd	3rd	4th	5th	Mean	SD	P-value (vs. Control)
Control	100.00%	100.00%	100.00%	100.00%	100.00%	100.00%	0.00%	
1μM	78.15%	71.65%	78.63%	95.54%	93.02%	83.40%	10.35%	0.11
5μM	63.68%	65.03%	67.01%	76.37%	81.30%	70.68%	7.74%	0.003
7.5μM			49.24%	72.45%	56.11%	59.27%	11.92%	<0.001
10μM	26.61%	1.57%	15.03%	46.55%	41.61%	26.27%	18.60%	<0.001

T3M4

	Proliferation						
Concentration	1st	2nd	3rd	4th	Mean	SD	P-value (vs. Control)
Control	100.00%	100.00%	100.00%	100.00%	100.00%	0.00%	
1μM	94.23%	93.75%	84.29%	79.37%	87.91%	7.30%	0.001
5μM	50.00%	55.21%	44.29%	56.08%	51.40%	5.44%	0.02
10μM	6.73%	15.10%	3.21%	15.61%	10.16%	6.17%	<0.001
	Metabolic activity						
Concentration	1st	2nd	3rd	4th	Mean	SD	P-value (vs. Control)
Control	100.00%	100.00%	100.00%	100.00%	100.00%	0.00%	
1μM	109.20%	110.61%	105.82%	91.65%	104.32%	8.68%	0.87
5μM	68.14%	88.89%	71.93%	89.89%	79.71%	11.29%	0.03
10μM	19.18%	39.12%	35.41%	52.80%	36.63%	13.83%	<0.001
	Biomass						
Concentration	1st	2nd	3rd	4th	Mean	SD	P-value (vs. Control)
Control	100.00%	100.00%	100.00%	100.00%	100.00%	0.00%	
1μM	80.93%	86.33%	93.09%	84.65%	86.25%	5.09%	0.08
5μM	51.84%	42.17%	69.17%	72.90%	59.02%	14.50%	<0.001
10μM	7.16%	9.14%	17.96%	11.95%	11.55%	4.70%	<0.001

Table S2 - IC50 MK-2206 (μM)

	Proliferation	Metabolic Activity	Cell Biomass
**AsPc-1**	4.412	8.307	4.511
**BxPc-3**	2.943	7.233	2.341
**Capan-1**	3.685	8.322	6.410
**Colo357**	7.508	10.06	6.025
**Panc-1**	3.622	11.66	3.828
**PaTu8902**	4.057	11.12	3.021
**PaTu8988S**	3.531	7.964	2.024
**PaTu8988T**	3.547	12.15	5.111
**SU.86.86**	6.659	8.892	7.340
**T3M4**	4.975	8.204	5.541

Table S3 - Cell Viability Buparlisib (to 100%Control)

AsPc-1

	Proliferation							
Concentration	1st	2nd	3rd	4th	5th	Mean	SD	P-value (vs. Control)
Control	100.00%	100.00%	100.00%	100.00%	100.00%	100.00%	0.00%	
1μM	70.27%	78.57%	65.94%	47.06%	60.00%	64.37%	11.81%	<0.001
2.5μM			17.19%	7.35%	19.23%	14.59%	6.35%	<0.001
5μM	12.16%	14.29%	5.00%	2.94%	3.94%	7.67%	5.18%	<0.001
10μM	4.05%	9.18%	0.50%	0.74%	0.89%	3.07%	3.71%	<0.001
	Metabolic activity							
Concentration	1st	2nd	3rd	4th	5th	Mean	SD	P-value (vs. Control)
Control	100.00%	100.00%	100.00%	100.00%	100.00%	100.00%	0.00%	
1μM	88.00%	74.91%	106.05%	81.83%	82.46%	86.65%	11.80%	0.06
2.5μM			48.03%	36.02%	37.70%	40.58%	6.50%	<0.001
5μM	17.90%	19.00%	23.41%	15.65%	19.20%	19.03%	2.82%	<0.001
10μM	9.72%	10.38%	10.61%	11.72%	12.61%	11.01%	1.15%	<0.001
	Biomass							
Concentration	1st	2nd	3rd	4th	5th	Mean	SD	P-value (vs. Control)
Control	100.00%	100.00%	100.00%	100.00%	100.00%	100.00%	0.00%	
1μM	54.89%	48.39%	54.40%	54.78%	61.83%	54.86%	4.76%	<0.001
2.5μM			14.04%	10.72%	10.94%	11.90%	1.86%	<0.001
5μM	4.55%	5.03%	1.99%	5.44%	8.38%	5.08%	2.28%	<0.001
10μM	1.76%	2.44%	1.17%	2.60%	1.86%	1.97%	0.57%	<0.001

BxPc-3

	Proliferation					
Concentration	1st	2nd	3rd	Mean	SD	P-value (vs. Control)
Control	100.00%	100.00%	100.00%	100.00%	0.00%	
0.5μM	81.08%	79.52%	80.84%	80.48%	0.84%	<0.001
1μM	48.28%	33.77%	43.33%	41.79%	7.38%	<0.001
5μM	4.35%	1.47%	3.69%	3.17%	1.51%	<0.001
10μM	1.45%	2.65%	0.31%	1.47%	1.17%	<0.001
	Metabolic activity					
Concentration	1st	2nd	3rd	Mean	SD	P-value (vs. Control)
Control	100.00%	100.00%	100.00%	100.00%	0.00%	
0.5μM	84.24%	94.92%	61.08%	80.08%	17.30%	0.03
1μM	28.55%	58.59%	51.56%	46.23%	15.71%	<0.001
5μM	7.77%	9.87%	8.94%	8.86%	1.05%	<0.001
10μM	5.66%	6.35%	7.57%	6.53%	0.97%	<0.001
	Biomass					
Concentration	1st	2nd	3rd	Mean	SD	P-value (vs. Control)
Control	100.00%	100.00%	100.00%	100.00%	0.00%	
0.5μM	56.95%	47.23%	51.80%	51.99%	4.86%	<0.001
1μM	37.15%	54.18%	30.41%	40.58%	12.25%	<0.001
5μM	2.27%	2.09%	2.34%	2.23%	0.13%	<0.001
10μM	1.29%	0.97%	1.95%	1.40%	0.50%	<0.001

Capan-1

	Proliferation							
Concentration	1st	2nd	3rd			Mean	SD	P-value (vs. Control)
Control	100.00%	100.00%	100.00%			100.00%	0.00%	
1μM	45.45%	61.33%	43.57%			50.12%	9.76%	<0.001
5μM	3.03%	10.00%	4.29%			5.77%	3.71%	<0.001
10μM	4.55%	10.00%	5.00%			6.52%	3.03%	<0.001
	Metabolic activity							
	1st	2nd	3rd			Mean	SD	P-value (vs. Control)
Control	100.00%	100.00%	100.00%			100.00%	0.00%	
1μM	71.50%	85.94%	74.26%			77.23%	7.67%	0.01
5μM	28.67%	27.50%	15.53%			23.90%	7.27%	<0.001
10μM	17.81%	32.13%	14.87%			21.60%	9.23%	<0.001
	Biomass							
	1st	2nd	3rd	4th	5th	Mean	SD	P-value (vs. Control)
Control	100.00%	100.00%	100.00%	100.00%	100.00%	100.00%	0.00%	
1μM	48.97%	57.98%	62.35%	45.57%	48.76%	52.73%	7.09%	<0.001
5μM	11.40%	9.46%	8.54%	2.74%	2.52%	6.93%	4.06%	<0.001
10μM	7.72%	2.83%	6.17%	2.35%	0.79%	3.97%	2.87%	<0.001

Colo357

	Proliferation					Mean	SD	P-value (vs. Control)
Concentration	1st	2nd	3rd	4th	5th	Mean	SD	P-value (vs. Control)
Control	100.00%	100.00%	100.00%	100.00%	100.00%	100.00%	0.00%	
0.5μM	51.24%	50.56%	63.60%	47.58%	43.16%	51.23%	7.61%	<0.001
1μM	34.73%	44.94%	26.75%	8.87%	8.77%	24.81%	15.96%	<0.001
5μM	3.05%	7.30%	5.26%	3.63%	2.46%	4.34%	1.96%	<0.001
	Metabolic activity							
Concentration	1st	2nd	3rd	4th		Mean	SD	P-value (vs. Control)
Control	100.00%	100.00%	100.00%	100.00%		100.00%	0.00%	
0.5μM	81.75%	94.74%	78.76%	62.65%		79.48%	13.19%	0.01
1μM	61.87%	34.88%	44.71%	35.45%		44.23%	12.60%	<0.001
5μM	18.14%	12.65%	4.14%	10.86%		11.45%	5.77%	<0.001
	Biomass							
Concentration	1st	2nd	3rd	4th		Mean	SD	P-value (vs. Control)
Control	100.00%	100.00%	100.00%	100.00%		100.00%	0.00%	
0.5μM	74.72%	100.63%	87.67%	69.95%		83.24%	13.80%	0.11
1μM	66.80%	42.78%	32.50%	23.19%		41.32%	18.78%	<0.001
5μM	5.71%	6.57%	4.49%	3.28%		5.01%	1.44%	<0.001

Panc-1

	Proliferation			Mean	SD	P-value (vs. Control)
Concentration	1st	2nd	3rd	Mean	SD	P-value (vs. Control)
Control	100.00%	100.00%	100.00%	100.00%	0.00%	
1μM	66.04%	64.74%	62.82%	64.53%	1.62%	<0.001
5μM	7.55%	20.53%	10.90%	12.99%	6.74%	<0.001
10μM	9.43%	12.11%	11.54%	11.03%	1.41%	<0.001
	Metabolic activity					
Concentration	1st	2nd	3rd	Mean	SD	P-value (vs. Control)
Control	100.00%	100.00%	100.00%	100.00%	0.00%	
1μM	85.20%	74.31%	61.35%	73.62%	11.94%	0.02
5μM	43.41%	30.90%	22.58%	32.30%	10.49%	<0.001
10μM	33.17%	25.17%	17.93%	25.42%	7.62%	<0.001
	Biomass					
Concentration	1st	2nd	3rd	Mean	SD	P-value (vs. Control)
Control	100.00%	100.00%	100.00%	100.00%	0.00%	
1μM	95.74%	92.15%	49.86%	79.25%	25.52%	0.24
5μM	24.24%	32.46%	13.06%	23.25%	9.74%	<0.001
10μM	11.87%	23.10%	8.62%	14.53%	7.60%	<0.001

PaTu8902

	Proliferation					Mean	SD	P-value (vs. Control)
Concentration	1st	2nd	3rd	4th	5th	Mean	SD	P-value (vs. Control)
Control	100.00%	100.00%	100.00%	100.00%	100.00%	100.00%	0.00%	
0.5μM	60.45%	65.00%	67.31%	63.82%	62.50%	63.82%	2.58%	<0.001
1μM	34.99%	42.00%	52.88%	26.32%	47.32%	40.70%	10.41%	<0.001
2.5μM	6.74%	29.00%	9.62%	5.26%	13.39%	12.80%	9.57%	<0.001
	Metabolic activity							
Concentration	1st	2nd	3rd			Mean	SD	P-value (vs. Control)
Control	100.00%	100.00%	100.00%			100.00%	0.00%	
0.5μM	91.43%	82.15%	86.30%			86.63%	4.65%	0.34
1μM	78.44%	77.17%	86.46%			80.69%	5.04%	0.13
2.5μM	56.69%	20.46%	52.82%			43.32%	19.89%	<0.001
	Biomass							
Concentration	1st	2nd	3rd			Mean	SD	P-value (vs. Control)
Control	100.00%	100.00%	100.00%			100.00%	0.00%	
0.5μM	56.56%	77.20%	76.40%			70.05%	11.69%	0.02
1μM	36.02%	61.74%	67.25%			55.00%	16.67%	0.002
2.5μM	11.63%	11.22%	11.89%			11.58%	0.34%	<0.001

PaTu8988S

Concentration	1st	2nd	3rd	4th	Mean	SD	P-value (vs. Control)
	Proliferation						
Concentration	1st	2nd	3rd	4th	Mean	SD	P-value (vs. Control)
Control	100.00%	100.00%	100.00%		100.00%	0.00%	
0.5μM	86.44%	87.10%	88.70%		87.41%	1.16%	0.001
1μM	52.54%	62.37%	53.26%		56.06%	5.48%	<0.001
2.5μM	8.47%	8.60%	8.70%		8.59%	0.12%	<0.001
	Metabolic activity						
Concentration	1st	2nd	3rd	4th	Mean	SD	P-value (vs. Control)
Control	100.00%	100.00%	100.00%	100.00%	100.00%	0.00%	
0.5μM	80.82%	79.03%	86.91%	79.51%	81.57%	3.64%	0.003
1μM	56.90%	45.58%	67.97%	49.56%	55.00%	9.83%	<0.001
2.5μM	42.91%	28.10%	37.23%	41.21%	37.36%	6.62%	<0.001
	Biomass						
Concentration	1st	2nd	3rd	4th	Mean	SD	P-value (vs. Control)
Control	100.00%	100.00%	100.00%	100.00%	100.00%	0.00%	
0.5μM	74.33%	81.28%	70.55%	80.76%	76.73%	5.19%	0.005
1μM	43.89%	73.99%	60.91%	59.40%	59.55%	12.32%	<0.001
2.5μM	33.38%	44.88%	22.66%	25.21%	31.53%	10.00%	<0.001

PaTu8988T

Concentration	1st	2nd	3rd	4th	Mean	SD	P-value (vs. Control)
	Proliferation						
Concentration	1st	2nd	3rd	4th	Mean	SD	P-value (vs. Control)
Control	100.00%	100.00%	100.00%	100.00%	100.00%	0.00%	
0.5μM	45.32%	45.82%	52.69%	56.20%	50.01%	5.32%	<0.001
1μM	22.55%	20.86%	22.71%	38.32%	26.11%	8.18%	<0.001
2.5μM	13.73%	16.55%	12.75%	22.75%	16.45%	4.50%	<0.001
	Metabolic activity						
Concentration	1st	2nd	3rd	4th	Mean	SD	P-value (vs. Control)
Control	100.00%	100.00%	100.00%	100.00%	100.00%	0.00%	
0.5μM	83.33%	88.54%	79.98%	81.62%	83.37%	3.71%	0.14
1μM	31.34%	69.39%	49.55%	72.75%	55.76%	19.23%	<0.001
2.5μM	25.23%	22.12%	29.14%	29.62%	26.53%	3.54%	<0.001
	Biomass						
Concentration	1st	2nd	3rd	4th	Mean	SD	P-value (vs. Control)
Control	100.00%	100.00%	100.00%	100.00%	100.00%	0.00%	
0.5μM	71.71%	82.33%	59.19%	71.41%	71.16%	9.46%	<0.001
1μM	56.31%	54.43%	65.23%	55.61%	57.90%	4.95%	<0.001
2.5μM	21.03%	22.34%	22.90%	27.52%	23.45%	2.83%	<0.001

SU.86.86

Concentration	1st	2nd	3rd	4th	5th	Mean	SD	P-value (vs. Control)
	Proliferation							
Concentration	1st	2nd	3rd	4th	5th	Mean	SD	P-value (vs. Control)
Control	100.00%	100.00%	100.00%	100.00%	100.00%	100.00%	0.00%	
1μM	81.33%	79.89%	72.06%	81.08%	93.75%	81.62%	7.78%	<0.001
2.5μM			40.69%	58.56%	57.69%	52.31%	10.08%	<0.001
5μM	14.00%	16.85%	16.18%	30.63%	19.23%	19.38%	6.56%	<0.001
10μM	12.00%	14.67%	15.20%	15.77%	9.62%	13.45%	2.58%	<0.001
	Metabolic activity							
Concentration	1st	2nd	3rd	4th	5th	Mean	SD	P-value (vs. Control)
Control	100.00%	100.00%	100.00%	100.00%	100.00%	100.00%	0.00%	
1μM	89.48%	105.41%	85.84%	85.92%	87.26%	90.78%	8.31%	0.04
2.5μM			56.61%	62.07%	75.45%	64.71%	9.69%	<0.001
5μM	46.69%	60.65%	39.47%	42.31%	38.66%	45.56%	9.00%	<0.001
10μM	39.32%	40.85%	23.10%	28.64%	14.77%	29.34%	11.00%	<0.001
	Biomass							
Concentration	1st	2nd	3rd	4th	5th	Mean	SD	P-value (vs. Control)
Control	100.00%	100.00%	100.00%	100.00%	100.00%	100.00%	0.00%	
1μM	52.98%	71.59%	68.68%	69.51%	84.75%	69.50%	11.30%	<0.001
2.5μM			30.60%	43.05%	44.32%	39.32%	7.58%	<0.001
5μM	16.95%	15.12%	15.73%	12.03%	13.94%	14.75%	1.87%	<0.001
10μM	16.23%	14.81%	12.62%	15.31%	9.63%	13.72%	2.64%	<0.001

T3M4

	Proliferation					
Concentration	1st	2nd	3rd	Mean	SD	P-value (vs. Control)
Control	100.00%	100.00%	100.00%	100.00%	0.00%	
0.5µM	46.15%	41.67%	51.85%	46.56%	5.10%	<0.001
1µM	22.60%	27.08%	29.10%	26.26%	3.33%	<0.001
2.5µM	1.92%	2.08%	2.91%	2.30%	0.53%	<0.001
	Metabolic activity					
Concentration	1st	2nd	3rd	Mean	SD	P-value (vs. Control)
Control	100.00%	100.00%	100.00%	100.00%	0.00%	
0.5µM	63.74%	66.68%	70.45%	66.96%	3.36%	0.006
1µM	30.78%	26.28%	59.70%	38.92%	18.14%	<0.001
2.5µM	3.57%	4.23%	7.74%	5.18%	2.24%	<0.001
	Biomass					
Concentration	1st	2nd	3rd	Mean	SD	P-value (vs. Control)
Control	100.00%	100.00%	100.00%	100.00%	0.00%	
0.5µM	51.20%	64.81%	59.89%	58.63%	6.89%	<0.001
1µM	26.72%	16.09%	32.77%	25.19%	8.44%	<0.001
2.5µM	4.74%	3.43%	5.06%	4.41%	0.86%	<0.001

Table S4 - IC50 Buparlisib (μM)

	Proliferation	Metabolic Activity	Cell Biomass
**AsPc-1**	1.282	2.284	1.087
**BxPc-3**	0.8797	0.9969	0.6022
**Capan-1**	0.9985	2.473	1.068
**Colo357**	0.5104	0.9622	0.8822
**Panc-1**	1.512	2.723	2.419
**PaTu8902**	0.7478	2.168	0.9625
**PaTu8988S**	1.081	1.451	1.344
**PaTu8988T**	0.4824	1.240	1.159
**SU.86.86**	2.469	4.098	1.774
**T3M4**	0.4741	0.7471	0.5916

Table S5 - Apoptosis/Necrosis MK-2206 (%)

AsPc-1

	1st		2nd		3rd		Mean	SD	P-Value (vs. Control)
Concentration	G2(YP+/PI-)	G3(PI+)	G2(YP+/PI-)	G3(PI+)	G2(YP+/PI-)	G3(PI+)	G2+G3		
Control	0.63	4.30	3.45	5.06	1.89	2.49	5.94	1.83	
1μM	0.73	5.17	3.15	8.91	2.47	3.38	7.94	2.92	>0.99
5μM	0.41	9.49	1.62	9.27	2.03	7.85	10.22	0.47	0.42
10μM	0.11	12.6	1.01	11.2	1.73	11.4	12.68	0.38	0.01

BxPc-3

	1st		2nd		3rd		Mean	SD	P-Value (vs. Control)
Concentration	G2(YP+/PI-)	G3(PI+)	G2(YP+/PI-)	G3(PI+)	G2(YP+/PI-)	G3(PI+)	G2+G3		
Control	2.25	1.66	1.12	1.82	1.41	1.55	3.27	0.45	
1μM	2.87	4.61	1.72	3.60	1.68	3.40	5.96	1.08	0.02
5μM	1.02	3.81	1.61	3.66	1.34	3.60	5.01	0.19	0.34
10μM	1.45	4.95	1.05	3.70	0.94	4.05	5.38	0.73	0.21

Capan-1

	1st		2nd		3rd		Mean	SD	P-Value (vs. Control)
Concentration	G2(YP+/PI-)	G3(PI+)	G2(YP+/PI-)	G3(PI+)	G2(YP+/PI-)	G3(PI+)	G2+G3		
Control	6.60	5.70	1.75	4.94	1.68	4.34	8.34	2.82	
1μM	7.55	7.42	3.88	3.90	3.61	6.70	11.02	2.98	0.76
5μM	7.62	9.46	2.54	4.05	3.72	7.64	11.68	4.29	0.64
10μM	4.51	11.3	2.52	9.40	2.24	6.27	12.08	2.98	0.56

Colo357

	1st		2nd		3rd		4th		5th		Mean	SD	P-Value (vs. Control)
Concentration	G2(YP+/PI-)	G3(PI+)	G2(YP+/PI-)	G3(PI+)	G2(YP+/PI-)	G3(PI+)	G2(YP+/PI-)	G3(PI+)	G2(YP+/PI-)	G3(PI+)	G2+G3		
Control	3.05	2.17	2.13	1.42	2.87	1.11	1.24	0.90	1.70	1.00	3.52	1.07	
1μM	1.58	1.6	3.46	2.45	2.35	1.01	1.22	1.69	3.31	2.17	4.17	1.26	0.84
5μM	3.81	1.16	2.72	3.84	3.21	1.39	1.67	2.05	3.69	3.65	5.44	1.32	0.15
10μM	1.73	3.32	1.44	5.91	1.66	2.82	1.30	4.77	4.09	5.10	6.43	1.69	0.02

Panc-1

	1st		2nd		3rd		Mean	SD	P-Value (vs. Control)
Concentration	G2(YP+/PI-)	G3(PI+)	G2(YP+/PI-)	G3(PI+)	G2(YP+/PI-)	G3(PI+)	G2+G3		
Control	0.76	1.27	1.1	0.55	0.34	1.12	1.71	0.24	
1μM	0.35	1.03	0.27	0.86	0.92	1.35	1.59	0.49	>0.99
5μM	0.36	0.99	0.36	2.31	0.98	2.73	2.58	0.97	0.65
10μM	0.58	4.97	0.42	2.78	0.83	1.59	3.72	1.33	0.11

PaTu8902

	1st		2nd		3rd		Mean	SD	P-Value (vs. Control)
Concentration	G2(YP+/PI-)	G3(PI+)	G2(YP+/PI-)	G3(PI+)	G2(YP+/PI-)	G3(PI+)	G2+G3		
Control	2.42	2.41	0.48	2.77	0.37	0.90	3.12	1.46	
1μM	0.22	1.31	0.29	1.52	0.21	0.91	1.49	0.28	0.15
5μM	0.18	1.49	0.12	1.33	0.12	0.88	1.37	0.28	0.12
10μM	0.18	1.54	0.11	1.11	0.087	0.92	1.32	0.30	0.11

PaTu8988S

	1st		2nd		3rd		Mean	SD	P-Value (vs. Control)
Concentration	G2(YP+/PI-)	G3(PI+)	G2(YP+/PI-)	G3(PI+)	G2(YP+/PI-)	G3(PI+)	G2+G3		
Control	5.01	12.7	4.82	17.7	3.4	13.9	19.18	2.37	
1μM	3.41	9.78	1.90	10.5	2.73	10.7	13.01	0.44	0.42
5μM	1.86	6.84	0.52	10.1	1.63	13.7	11.55	2.79	0.09
10μM	1.13	8.14	0.46	8.75	0.97	11.5	10.32	1.52	0.04

PaTu8988T

	1st		2nd		3rd		Mean	SD	P-Value (vs. Control)
Concentration	G2(YP+/PI-)	G3(PI+)	G2(YP+/PI-)	G3(PI+)	G2(YP+/PI-)	G3(PI+)	G2+G3		
Control	0.10	0.94	0.260	3.92	0.010	1.19	2.14	1.45	
1μM	0.11	1.24	0.086	3.03	0.005	0.96	1.81	0.94	>0.99
5μM	0.07	1.14	0.029	2.06	0.005	0.78	1.36	0.54	>0.99
10μM	0.02	0.98	0.048	4.73	0.000	0.94	2.24	1.80	>0.99

SU.86.86

	1st		2nd		3rd		Mean	SD	P-Value (vs. Control)
Concentration	G2(YP+/PI-)	G3(PI+)	G2(YP+/PI-)	G3(PI+)	G2(YP+/PI-)	G3(PI+)	G2+G3		
Control	1.40	2.73	1.98	4.49	1.47	4.11	3.78	0.76	
1μM	0.41	3.3	1.42	4.19	3.22	10.80	6.10	3.35	0.68
7.5μM	0.31	4.14	1.87	6.29	1.99	6.37	5.60	1.03	0.87
10μM	0.68	5.24	1.71	7.18	1.29	4.92	5.78	1.00	0.86

T3M4

	1st		2nd		3rd		Mean	SD	P-Value (vs. Control)
Concentration	G2(YP+/PI-)	G3(PI+)	G2(YP+/PI-)	G3(PI+)	G2(YP+/PI-)	G3(PI+)	G2+G3		
Control	8.02	2.66	6.00	1.85	8.58	3.96	10.36	1.93	
1μM	7.21	3.62	6.80	2.03	7.55	5.05	10.75	1.54	0.99
5μM	4.30	3.89	4.26	2.80	3.21	3.28	7.25	0.71	0.18
10μM	4.04	7.97	2.66	7.57	2.91	5.01	10.05	1.67	>0.99

G2: Apoptosis
G3: Necrosis
G2+G3: Cell Deaths

Table S6 - Apoptosis/Necrosis Buparlisib (%)

AsPc-1

	1st		2nd		3rd		4th		5th		Mean	SD	P-Value (vs. Control)
Concentration	G2(YP+/PI-)	G3(PI+)	G2(YP+/PI-)	G3(PI+)	G2(YP+/PI-)	G3(PI+)	G2(YP+/PI-)	G3(PI+)	G2(YP+/PI-)	G3(PI+)	G2+G3		
Control	0.51	4.53	3.26	5.64	1.60	3.05	1.63	5.47	1.08	3.63	6.08	1.67	
1μM	0.66	11.5	3.13	14.9	2.66	9.9	2.72	8.60	2.23	6.35	12.53	3.08	0.15
2.5μM	1.13	37.8	2.39	36.7	6.20	32.3	2.77	46.4	2.94	39.8	41.69	4.04	<0.001
5μM	0.42	48.8	0.86	67.0	4.53	54.8	1.87	66.8	1.79	64.7	62.31	7.33	<0.001

BxPc-3

	1st		2nd		3rd		Mean	SD	P-Value (vs. Control)
Concentration	G2(YP+/PI-)	G3(PI+)	G2(YP+/PI-)	G3(PI+)	G2(YP+/PI-)	G3(PI+)	G2+G3		
Control	0.55	2.23	1.86	1.92	1.68	3.07	3.77	0.80	
1μM	0.56	14.2	1.35	4.63	2.52	5.68	9.65	3.73	0.52
5μM	0.04	88.6	1.58	65.9	1.09	75.2	77.47	8.68	<0.001
10μM	0.19	85.7	1.08	81.3	0.73	87.7	85.57	2.48	<0.001

Capan-1

	1st		2nd		3rd		Mean	SD	P-Value (vs. Control)
Concentration	G2(YP+/PI-)	G3(PI+)	G2(YP+/PI-)	G3(PI+)	G2(YP+/PI-)	G3(PI+)	G2+G3		
Control	1.67	6.88	1.55	4.76	5.01	9.13	9.67	3.29	
1μM	2.13	8.63	1.99	9.67	5.79	7.12	11.78	0.88	0.74
5μM	6.62	32.0	5.18	25.6	4.46	29.9	34.59	3.20	0.004
10μM	3.49	34.6	4.50	27.5	4.79	37.3	37.39	4.15	0.005

Colo357

	1st		2nd		3rd		Mean	SD	P-Value (vs. Control)
Concentration	G2(YP+/PI-)	G3(PI+)	G2(YP+/PI-)	G3(PI+)	G2(YP+/PI-)	G3(PI+)	G2+G3		
Control	4.00	2.20	3.31	1.64	1.59	1.04	4.59	1.48	
0.5μM	3.53	2.38	4.92	4.53	2.89	2.72	6.99	1.74	0.24
1μM	3.89	1.74	3.71	5.49	2.86	3.44	7.04	1.55	0.24
5μM	3.24	19.6	2.51	21.0	1.97	19.4	22.57	0.89	<0.001

Panc-1

	1st		2nd		3rd		Mean	SD	P-Value (vs. Control)
Concentration	G2(YP+/PI-)	G3(PI+)	G2(YP+/PI-)	G3(PI+)	G2(YP+/PI-)	G3(PI+)	G2+G3		
Control	0.60	1.15	0.56	0.58	0.18	1.08	1.38	0.26	
1μM	0.46	1.25	1.16	1.60	0.33	0.66	1.82	0.73	0.81
5μM	0.55	1.66	1.25	2.54	1.50	2.11	3.20	0.71	0.04
10μM	0.78	3.47	1.52	3.76	1.86	3.77	5.05	0.59	<0.001

PaTu8902

	1st		2nd		3rd		Mean	SD	P-Value (vs. Control)
Concentration	G2(YP+/PI-)	G3(PI+)	G2(YP+/PI-)	G3(PI+)	G2(YP+/PI-)	G3(PI+)	G2+G3		
Control	0.32	1.96	0.49	2.81	0.24	2.21	2.68	0.45	
1μM	0.13	2.20	0.31	3.61	0.14	4.36	3.58	0.92	0.99
2.5μM	0.12	8.53	0.28	12.5	0.32	14.2	11.98	2.46	0.01
5μM	0.20	9.97	0.37	17.4	0.23	19.4	15.86	4.09	0.001
10μM	0.11	25.1	0.33	30.1	0.19	23.5	26.44	2.89	<0.001

PaTu8988S

	1st		2nd		3rd		Mean	SD	P-Value (vs. Control)
Concentration	G2(YP+/PI-)	G3(PI+)	G2(YP+/PI-)	G3(PI+)	G2(YP+/PI-)	G3(PI+)	G2+G3		
Control	4.93	8.70	2.45	9.28	3.64	5.27	11.42	1.94	
1μM	3.56	9.06	2.91	5.31	3.32	12.4	12.19	3.08	>0.99
2.5μM	5.30	11.3	2.11	13.9	2.82	8.19	14.54	2.51	0.55
5μM	3.70	12.2	2.93	14.2	2.96	13.4	16.46	0.51	0.19
10μM	3.57	27.5	3.42	19.8	3.09	23.3	26.89	3.22	<0.001

PaTu8988T

	1st		2nd		3rd		Mean	SD	P-Value (vs. Control)
Concentration	G2(YP+/PI-)	G3(PI+)	G2(YP+/PI-)	G3(PI+)	G2(YP+/PI-)	G3(PI+)	G2+G3		
Control	0.63	0.99	0.62	2.39	1.79	1.79	2.74	0.82	
1μM	0.17	1.13	0.34	2.00	0.37	2.62	2.21	0.70	>0.99
2.5μM	0.15	6.53	0.24	8.25	0.20	8.89	8.09	1.02	0.13
5μM	0.12	6.35	0.26	10.7	0.48	13.1	10.34	2.94	0.03
10μM	0.053	11.2	0.12	9.48	0.025	18.7	13.19	3.97	0.004

SU.86.86

	1st		2nd		3rd		Mean	SD	P-Value (vs. Control)
Concentration	G2(YP+/PI-)	G3(PI+)	G2(YP+/PI-)	G3(PI+)	G2(YP+/PI-)	G3(PI+)	G2+G3		
Control	1.75	4.01	1.8	5.89	1.62	5.69	6.92	0.83	
1μM	0.70	4.26	0.77	3.28	1.15	3.08	4.41	0.39	0.15
2.5μM	1.27	4.76	0.97	4.96	1.31	6.13	6.47	0.69	0.96
5μM	1.43	9.14	0.86	12.1	1.91	13.7	13.05	2.06	0.002

T3M4

	1st		2nd		3rd		Mean	SD	P-Value (vs. Control)
Concentration	G2(YP+/PI-)	G3(PI+)	G2(YP+/PI-)	G3(PI+)	G2(YP+/PI-)	G3(PI+)	G2+G3		
Control	5.23	3.56	3.04	2.63	7.66	3.78	8.63	2.36	
1μM	2.86	5.12	4.15	6.63	3.74	5.37	9.29	1.15	>0.99
1.5μM	4.63	21.9	4.27	15.5	4.29	22.6	24.40	3.27	<0.001
2.5μM	4.74	41.3	3.51	43.3	3.04	46.6	47.50	1.55	<0.001
5μM	2.13	55.1	2.32	50.4	1.56	54.0	55.17	1.86	<0.001
10μM	2.30	47.6	2.32	51.0	1.92	49.4	51.51	1.40	<0.001

G2: Apoptosis
G3: Necrosis
G2+G3: Cell Deaths

Table S7 - Target Gene Expression (Log2(TPM+1))

	MK-2206			Buparlisib			
	AKT1	*AKT2*	*AKT3*	*PIK3CA*	*PIK3CB*	*PIK3CG*	*PIK3CD*
**AsPc-1**	6.25	4.32	0.00	4.57	5.86	0.00	0.26
**BxPc-3**	7.38	5.41	2.79	4.28	4.87	0.85	2.00
**Capan-1**	7.18	5.52	2.72	3.98	4.33	3.74	1.73
**Colo357**	5.66	5.27	1.56	3.68	4.53	0.00	0.89
**Panc-1**	8.20	9.68	4.23	4.97	5.97	0.00	3.54
**SU.86.86**	7.05	7.32	4.75	4.23	4.44	1.08	3.13
**PaTu8988S**	7.26	4.80	0.07	4.47	5.28	0.00	0.24
**PaTu8988T**	6.94	4.66	5.67	4.42	4.61	0.10	3.48
**PaTu8902**	7.16	4.77	5.76	4.37	4.94	0.00	3.11
**T3M4**	6.23	4.91	2.45	4.29	3.73	0.00	1.73
**Control**	5.13	5.13	1.52	1.52	3.10	0.12	1.08

Table S8 – MK-2206 Target Gene Variants in PDAC Cell Lines

Cell line	#Chromesome	Start	End	Reference	Observed	Zygosities	Variant Confidance	Variant Allele Frequency	Reading Depth	Gene	Base Change	Animo Acin Change	Variant Type
BxPc-3	chr14	105241573	105241573	G	A	het	473.77	34.7	72	*AKT1*	NM_001014431.1:c.436-29C>T	-	intron_variant
PaTu8902	chr14	105259122	105259122	G	A	het	277.77	78.6	14	*AKT1*	NM_001014431.1:c.-79-63C>T	-	intron_variant
PaTu8988T	chr14	105259122	105259122	G	A	het	365.77	54.2	24	*AKT1*	NM_001014431.1:c.-79-63C>T	-	intron_variant
PaTu8988S	chr14	105259122	105259122	G	A	het	91.77	29.4	17	*AKT1*	NM_001014431.1:c.-79-63C>T	-	intron_variant
SU.86.86	chr19	40739770	40739770	G	A	het	737.77	23.6	254	*AKT2*	NM_001626.5:c.*9C>T	-	3_prime_UTR_variant
SU.86.86	chr19	40748010	40748010	G	A	het	2690.77	83.1	124	*AKT2*	NM_001626.5:c.442-34C>T	-	intron_variant
Panc-1	chr1	243709052	243709052	T	C	het	125.77	45.5	11	*AKT3*	NM_005465.4:c.1164-153A>G	-	intron_variant
PaTu8988S	chr1	243716481	243716494	C	CA	het	22.64	66.7	3	*AKT3*	NM_005465.4:c.949-237dupT	-	intron_variant
PaTu8988S	chr1	243736210	243736225	C	CT	het	22.1	41.7	12	*AKT3*	NM_005465.4:c.819+17dupA	-	splice_region_variant & intron_variant

Table S9 - Buparlisib Target Gene Variants in PDAC Cell Lines

Cell line	#Chromesome	Start	End	Reference	Observed	Zygosities	Variant Confidance	Variant Allele Frequency	Reading Depth	Gene	Base Change	Animo Acin Change	Variant Type
Colo357	chr3	178916504	178916504	C	G	het	48.77	30	10	*PIK3CA*	NM_006218.2:c.-76-34C>G	-	intron_variant
Colo357	chr3	178922374	178922374	C	G	het	249.77	22.7	88	*PIK3CA*	NM_006218.2:c.1143C>G	NM_006218.2:p.Pro381Pro	splice_region_variant & synonymous_variant
Colo357	chr3	178937400	178937400	A	G	het	461.77	28.9	114	*PIK3CA*	NM_006218.2:c.1788A>G	NM_006218.2:p.Glu596Glu	synonymous_variant
Colo357	chr3	178942268	178942273	TATAT	A	hom	367.74	100	24	*PIK3CA*	NM_006218.2:c.2295-220_2295-216delTATATinsA	-	intron_variant
PaTu8988T	chr3	178942268	178942273	TATAT	A	het	19.25	66.7	3	*PIK3CA*	NM_006218.2:c.2295-220_2295-216delTATATinsA	-	intron_variant
T3M4	chr3	178917762	178917775	CTT	C	het	16.85	44.4	9	*PIK3CA*	NM_006218.2:c.562+87_562+88delTT	-	intron_variant
AsPc-1	chr3	138430856	138430861	AAAG	A	het	15.39	66.7	6	*PIK3CB*	NM_006219.2:c.1399+191_1399+193delCTT	-	upstream_gene_variant
AsPc-1	chr3	138456486	138456507	AAT	A	het	40.47	25	12	*PIK3CB*	NM_006219.2:c.801+61_801+62delAT	-	intron_variant
Panc-1	chr3	138456486	138456507	AAT	A	het	11.77	18.2	11	*PIK3CB*	NM_006219.2:c.801+61_801+62delAT	-	intron_variant
Capan-1	chr3	138383723	138383723	G	A	het	132.77	55.6	9	*PIK3CB*	NM_006219.2:c.2672+155C>T	-	intron_variant
Colo357	chr3	138423497	138423497	T	C	het	28.77	28.6	7	*PIK3CB*	NM_006219.2:c.1531-162A>G	-	intron_variant
Colo357	chr3	138425942	138425942	T	C	het	446.77	70.4	27	*PIK3CB*	NM_006219.2:c.1530+59A>G	-	intron_variant
Colo357	chr3	138456506	138456506	T	C	het	179.77	64.7	17	*PIK3CB*	NM_006219.2:c.801+43A>G	-	intron_variant
SU.86.86	chr3	138474414	138474414	G	A	hom	83.28	100	3	*PIK3CB*	NM_006219.2:c.397+182C>T	-	intron_variant
Capan-1	chr7	106520052	106520052	C	G	het	1756.77	52.9	153	*PIK3CG*	NM_001282426.1:c.2480C>G	NM_001282426.1:p.Thr827Arg	missense_variant
Colo357	chr1	9779801	9779801	T	C	het	12.06	66.7	3	*PIK3CD*	NM_005026.3:c.1243-178T>C	-	intron_variant
PaTu8902	chr1	9770690	9770713	CAGAGAG	C	het	36.47	26.7	15	*PIK3CD*	NM_005026.3:c.141+54_141+59delGAGAGA	-	intron_variant

Table S10 - *KRAS* Variants in PDAC Cell Lines

Cell line	#Chromesome	Start	End	Reference	Observed	Zygosities	Variant Confidance	Variant Allele Frequency	Reading Depth	Gene	Base Change	Animo Acin Change	Variant Type
AsPc-1	chr12	25398284	25398284	C	T	hom	2219.77	100	90	*KRAS*	NM_033360.2:c.35G>A	NM_033360.2:p.Gly12Asp	missense_variant
Capan-1	chr12	25398284	25398284	C	A	hom	1774.77	97.1	70	*KRAS*	NM_033360.2:c.35G>T	NM_033360.2:p.Gly12Val	missense_variant
Colo357	chr12	25398284	25398284	C	T	het	403.77	23.8	126	*KRAS*	NM_033360.2:c.35G>A	NM_033360.2:p.Gly12Asp	missense_variant
Panc-1	chr12	25398284	25398284	C	T	het	2675.77	62.1	203	*KRAS*	NM_033360.2:c.35G>A	NM_033360.2:p.Gly12Asp	missense_variant
PaTu8902	chr12	25398284	25398284	C	A	hom	1052.77	100	42	*KRAS*	NM_033360.2:c.35G>T	NM_033360.2:p.Gly12Val	missense_variant
PaTu8988T	chr12	25398284	25398284	C	A	hom	1212.77	98	49	*KRAS*	NM_033360.2:c.35G>T	NM_033360.2:p.Gly12Val	missense_variant
PaTu8988S	chr12	25398284	25398284	C	A	hom	1541.77	96.9	65	*KRAS*	NM_033360.2:c.35G>T	NM_033360.2:p.Gly12Val	missense_variant
SU.86.86	chr12	25398284	25398284	C	T	het	6018.77	83.7	319	*KRAS*	NM_033360.2:c.35G>A	NM_033360.2:p.Gly12Asp	missense_variant
T3M4	chr12	25380275	25380275	A	C	het	709.77	32.6	129	*KRAS*	NM_033360.2:c.183A>C	NM_033360.2:p.Gln61His	missense_variant

Table S11 - *TP53* Variants in PDAC Cell Lines

Cell line	#Chromesome	Start	End	Reference	Observed	Zygosities	Variant Confidance	Variant Allele Frequency	Reading Depth	Gene	Base Change	Animo Acin Change	Variant Type
AsPc-1	chr17	7578526	7578530	CA	C	hom	3365.73	96.4	110	*TP53*	NM_000546.4:c.403delT	NM_000546.4:p.Cys135fs	frameshift_variant
Panc-1	chr17	7577120	7577120	C	T	hom	1977.77	98.8	81	*TP53*	NM_000546.4:c.818G>A	NM_000546.4:p.Arg273His	missense_variant
BxPc-3	chr17	7578190	7578190	T	C	hom	2653.77	99	103	*TP53*	NM_000546.4:c.659A>G	NM_000546.4:p.Tyr220Cys	missense_variant
Capan-1	chr17	7578454	7578454	G	A	hom	2094.77	100	83	*TP53*	NM_000546.4:c.476C>T	NM_000546.4:p.Ala159Val	missense_variant
Colo357	chr17	7579419	7579424	AG	A	hom	4167.73	100	130	*TP53*	NM_000546.4:c.267delC	NM_000546.4:p.Ser90fs	frameshift_variant
PaTu8902	chr17	7577094	7577094	G	A	hom	2250.77	100	79	*TP53*	NM_000546.4:c.844C>T	NM_000546.4:p.Arg282Trp	missense_variant
PaTu8988T	chr17	7577094	7577094	G	A	hom	1503.77	100	57	*TP53*	NM_000546.4:c.844C>T	NM_000546.4:p.Arg282Trp	missense_variant
PaTu8988S	chr17	7577094	7577094	G	A	hom	2498.77	100	97	*TP53*	NM_000546.4:c.844C>T	NM_000546.4:p.Arg282Trp	missense_variant
SU.86.86	chr17	7573948	7573948	C	A	hom	665.77	100	24	*TP53*	NM_000546.4:c.1079G>T	NM_000546.4:p.Gly360Val	missense_variant
SU.86.86	chr17	7577548	7577548	C	T	hom	1415.77	100	54	*TP53*	NM_000546.4:c.733G>A	NM_000546.4:p.Gly245Ser	missense_variant
T3M4	chr17	7578190	7578190	T	C	hom	2061.77	100	78	*TP53*	NM_000546.4:c.659A>G	NM_000546.4:p.Tyr220Cys	missense_variant

Table S12 - Gene expression *KRAS* (Log2(TPM+1))

	KRAS Expression
**AsPc-1**	4.79
**BxPc-3**	4.53
**Capan-1**	4.40
**Colo357**	4.16
**Panc-1**	6.11
**PaTu8902**	4.51
**PaTu8988S**	4.65
**PaTu8988T**	4.46
**SU.86.86**	7.09
**T3M4**	5.79
**Control**	2.14

Table S13 - Gene expression *TP53* (Log2(TPM+1))

	***TP53* Expression**
**AsPc-1**	1.24
**BxPc-3**	5.42
**Capan-1**	4.39
**Colo357**	2.13
**Panc-1**	5.29
**PaTu8902**	5.37
**PaTu8988S**	5.35
**PaTu8988T**	5.34
**SU.86.86**	4.61
**T3M4**	5.26
**Control**	2.83
